# The autophagic protein p62 is a target of reactive aldehydes in human and murine cholestatic liver disease

**DOI:** 10.1371/journal.pone.0276879

**Published:** 2022-11-15

**Authors:** Colin T. Shearn, Aimee L. Anderson, Michael W. Devereux, David J. Orlicky, Cole Michel, Dennis R. Petersen, Colin G. Miller, Sanjiv Harpavat, Edward E. Schmidt, Ronald J. Sokol

**Affiliations:** 1 Department of Pediatrics, Pediatric Liver Center, Section of Pediatric Gastroenterology, Hepatology and Nutrition, and Children’s Hospital Colorado, Aurora, CO, United States of America; 2 Department of Pathology, School of Medicine, University of Colorado Anschutz Medical Campus, Aurora, CO, United States of America; 3 Pharmaceutical Sciences, School of Pharmacy, University of Colorado Anschutz Medical Campus, Aurora, CO, United States of America; 4 Department of Microbiology & Cell Biology, Montana State University, Bozeman, MT, United States of America; 5 Department of Pediatrics, Baylor College of Medicine and Texas Children’s Hospital, Houston, TX, United States of America; 6 Laboratory of Redox Biology, Departments of Pharmacology and Physiology, Hungarian Veterinary Medical University, Budapest, Hungary; Texas A&M University, UNITED STATES

## Abstract

Inflammatory cholestatic liver diseases, including Primary Sclerosing Cholangitis (PSC), are characterized by periportal inflammation with progression to cirrhosis. The objective of this study was to examine interactions between oxidative stress and autophagy in cholestasis. Using hepatic tissue from male acute cholestatic (bile duct ligated) as well as chronic cholestatic (Mdr2^KO^) mice, localization of oxidative stress, the antioxidant response and induction of autophagy were analyzed and compared to human PSC liver. Concurrently, the ability of reactive aldehydes to post-translationally modify the autophagosome marker p62 was assessed in PSC liver tissue and in cell culture. Expression of autophagy markers was upregulated in human and mouse cholestatic liver. Whereas mRNA expression of *Atg12*, *Lamp1*, *Sqstm1* and *Map1lc3* was increased in acute cholestasis in mice, it was either suppressed or not significantly changed in chronic cholestasis. In human and murine cholestasis, periportal hepatocytes showed increased IHC staining of ubiquitin, 4-HNE, p62, and selected antioxidant proteins. Increased p62 staining colocalized with accumulation of 4-HNE-modified proteins in periportal parenchymal cells as well as with periportal macrophages in both human and mouse liver. Mechanistically, p62 was identified as a direct target of lipid aldehyde adduction in PSC hepatic tissue and *in vitro* cell culture. *In vitro* LS-MS/MS analysis of 4-HNE treated recombinant p62 identified carbonylation of His^123^, Cys^128^, His^174^, His^181^, Lys^238^, Cys^290^, His^340^, Lys^341^ and His^385^. These data indicate that dysregulation of autophagy and oxidative stress/protein damage are present in the same periportal hepatocyte compartment of both human and murine cholestasis. Thus, our results suggest that both increased expression as well as ineffective autophagic degradation of oxidatively-modified proteins contributes to injury in periportal parenchymal cells and that direct modification of p62 by reactive aldehydes may contribute to autophagic dysfunction.

## Introduction

Cholestatic liver disease accounts for approximately 9.2% of adult and 43.1% of pediatric liver transplants in the United States [1–5]. Primary sclerosing cholangitis (PSC) is a progressive biliary disease of unknown etiology that affects up to 16.2 per 100,000 people in the US with no current FDA-approved effective therapies [6, 7]. Long-term disease progression leads to biliary obstruction, repeated bouts of cholangitis, and secondary biliary cirrhosis with a median time of survival following diagnosis of 12–18 years [8]. Currently, the only validated treatment for end-stage PSC is liver transplantation and even then, PSC is known to recur in 20–40% of patients [9]. Furthermore, unique to PSC, in the absence of liver transplant, patients carry a 10–20% lifetime risk of cholangiocarcinoma. The pathogenesis of PSC is poorly understood.

Autophagy is a normal and highly regulated mechanism in cells that functions to remove and recycle damaged components by proteolysis. These components can be normal proteins/lipids which would be recycled during starvation or damaged proteins/organelles as a result of disease processes. The autophagic machinery consists of numerous proteins including sequestosome 1 (Sqstm1, p62) which functions as a platform for autophagosome assembly and autophagy related (Atg) genes [10]. During chronic liver disease, dysregulation of autophagy may result in the cellular accumulation of damaged proteins and organelles leading to cellular dysfunction and reactive inflammation. Autophagy has recently been shown to play a significant role in cholestatic liver injury. Increased concentrations of retained bile acids suppress autophagic processing in hepatocytes [11, 12]. In human primary biliary cholangitis (PBC) and PSC, autophagy is upregulated and expression of p62 and LC3 is increased in biliary epithelial cells as well as periportal hepatocytes that surround damaged bile ducts [13–15]. In mouse models, autophagy is upregulated following bile duct ligation [12, 16] and mice deficient in Atg5 or Atg7 demonstrated increased accumulation of p62 that correlated with more severe intrahepatic cholestasis, thus suggesting that defective autophagy amplifies cholestatic liver injury [17].

A hallmark of chronic inflammatory diseases is an increased production of reactive oxygen species leading to increased lipid peroxidation of cellular membranes. The resultant accumulation of highly reactive lipid aldehydes can modify proteins through interactions with critical cysteine, lysine and histidine residues, thus impairing function [18]. During cholestatic liver injury, periportal hepatocytes undergo hepatocyte to cholangiocyte transdifferentiation [19, 20]. We have shown that carbonylation and oxidative injury is increased in periportal hepatocytes in liver from patients diagnosed with PSC [21, 22]. Recent evidence indicates that cellular antioxidant responses and autophagy are intrinsically linked. The transcription factor Nuclear factor erythroid 2-related factor 2 (Nrf2) regulates cellular antioxidant responses. In its inactive state, Nrf2 is sequestered in the cytosol by the Kelch Like ECH Associated Protein 1 (Keap1) [23]. Upon activation, Keap1 dissociates from Nrf2 and can physically interact with p62 promoting autophagy [24, 25].

The objective of this study was to examine the relationship between oxidative stress and autophagy in cholestasis by examining the colocalization of markers of oxidative injury and autophagy in hepatic tissue procured from human patients diagnosed with end stage PSC and in the bile duct ligation (BDL; acute) and *Abcb4*^KO^ (Mdr2^KO^; Chronic) models of murine obstructive cholestasis. We found that hepatic expression of autophagic proteins is increased in human PSC and in both acute and chronic cholestatic mice. BDL resulted in increased IHC staining of protein-4-HNE, histone H2Ax, and antioxidant proteins (Carbonyl reductase 3, Glutathione S-transferase mu). Furthermore, increased expression of the autophagic regulator p62 colocalized with proteins that have been post-translationally modified by reactive aldehydes. Importantly, colocalization was present in hepatocytes that were immediately adjacent to periportal areas of ongoing hepatic inflammation and fibrosis in both acute and chronic murine cholestasis.

## Methods

### Human sample procurement

For human studies, paraffin embedded and fresh frozen hepatic tissue explants from end-stage PSC patients (Table 1, N = 10; 5 females, 5 males, ages 25–62 yrs) that were procured during liver transplantation and control human livers (N = 6; 6 males, ages 37–62 yrs) were obtained from the University of Minnesota Liver Tissue Cell Distribution Center NIH Contract #HHSN276201200017C, as previously described [22, 26].

**Table 1:** Serum biochemical parameters of human PSC patients.

Number	Disease	SEX	Age	Tissue	Paraffin	MELD	Pro Throm INR	Total Bilirubin (mg/dl)	Creatinine (mg/dl)	AST (U/L)	Alk Phos (U/L)	Albumin (g/dl)	Comorbidity
1	PSC	FEMALE	41		Y	17	1.35	2.9	1.1	49	226	3.2	Crohn’s
2	PSC	MALE	42	Y	Y	ND	ND	ND	ND	ND	ND	ND	ND
3	PSC	MALE	25	Y		23	2.25	8.2	0.84	173	261	2.9	UC
4	PSC	FEMALE	51		Y	16	1.37	4.9	0.73	105	453	2.9	UC
5	PSC	MALE	62	Y		19	1.29	17.1	1.47	50	1006	3.5	UC
6	PSC	MALE	32	Y		14	1.05	5.1	0.8	86	874	3.9	UC
7	PSC	MALE	58		Y	17	1.14	10	0.75	129	548	2.8	UC
8	PSC	FEMALE	51	Y		ND	ND	ND	ND	ND	ND	ND	ND
9	PSC	FEMALE	39	Y		ND	ND	ND	ND	ND	ND	ND	ND
10	PSC	FEMALE	27		Y	21	1.64	9.5	0.73	66	369	2.2	UC

Data was obtained from the University of Minnesota Liver Tissue Cell Distribution Center, NIH Contract #HHSN276201200017C. MELD (Model for End Stage Liver Disease), INR (International Normalized Ratio), AST (Aspartate Aminotransferase), Alk Phos (Alkaline Phosphatase).

### Human ethics statement

Informed consent was obtained from each patient, and the study protocol conformed to the Ethical Guidelines of the 1975 Declaration of Helsinki. The research protocol was reviewed and approved by the Ethic Committees of the National Institutes of Health, and the regional committees for medical and health research ethics at the University of Minnesota. No donor organs were obtained from executed prisoners or other institutionalized individuals.

### Murine sample procurement

For murine studies, male C57BL6/J (WT) mice (8-10wks old; N = 5/group) were subjected to sham or BDL surgery as previously described [27]. After 3 days of BDL or sham surgery, mice were anesthetized and blood and liver removed [28, 29], plasma was separated by centrifugation at 5000 rpm for 5 min at 4°C and was assayed for alanine aminotransferase (ALT), aspartate amino transferase (AST), alkaline phosphatase activity and total bilirubin [30]. Whole livers were excised and weighed. Caudate and median lobes were removed, fixed in 10% neutral buffered formalin and embedded in paraffin for histological and immunohistochemical (IHC) analyses and the remaining hepatic tissue flash frozen in liquid nitrogen for Western blotting and qPCR as previously described [28]. For Mdr2^KO^ studies, liver samples were obtained from a previous study (male C57BL/6J and Mdr2^KO^ mice (10–12 weeks old; N = 6/group) [28]. All animal protocols were approved by the Institutional Animal Care and Use Committee of the University of Colorado (IACUC protocol#00000879) and were performed in accordance with published National Institutes of Health guidelines. No animals were removed from the study due to adverse events. All studies involving animal experiments conformed with the Animal Research: Reporting of In Vivo Experiments (ARRIVE) guidelines [31].

### Histological evaluation

Formalin fixed slides were stained using Picrosirius red or immunohistochemically evaluated using antibodies directed against 4-HNE (#6555 [32]), Nrf2 (ab31163, Abcam, Waltham, MA), GSTμ (ab53942, Abcam), phospho Serine 129 gamma H2Ax (9718, Cell Signaling Technologies, Danvers, MA), Ubiquitin (ab7254, Abcam), Heme Oxygenase 1 (SPA-895, Enzo Lifesciences, Framingham, NY), p62 (NBP1-48320, NBP1-48320AF594, NBP1-48320AF647, Novus Biologicals, Denver, CO), Cytokeratin 7 (CK7, ab181598, Abcam), LC3 (14600-1-ap Proteintech, Rosemont, IL), LC3b (#2775, Cell Signaling Technologies), LAMP1 (bs-1970R, Bioss Antibodies Inc, Woburn, MA), LAMP2 (NBP300-591, Novus Biologicals), CD68 (M0814, Agilent Technologies, Santa Clara, CA), F4/80 (MCA497, Biorad, Hercules, CA) as previously described [22, 26, 28, 32]. For light microscopy, staining was visualized using 3, 3′-diaminobenzidine (DAB) (SK4105, Vector laboratories, Newark, CA) and either a horse anti-rabbit (MP-7401, Vector laboratories) or a goat anti-rat (MP-7444, Vector Laboratories) secondary antibody. For fluorescent microscopy, staining was visualized using the following secondary antibodies: A Donkey anti-Rat IgG (H+L) Alexa Fluor 488 conjugate, a Goat anti-Rabbit, Alexa Fluor™ 647 conjugate or a Goat anti-Mouse Alexa Fluor® 594 conjugate (Invitrogen, Thermofisher, Waltham, MA). Immunohistochemical staining of CK7 was quantified with the Slidebook program version 6.0 (Intelligent Imaging Innovations, Denver, Colorado).

### Western blotting

Western blots were performed on liver homogenate using 20μg of protein per lane as previously described [32]. The GAPDH antibody was from Millipore (Billerica, MA) and all other antibodies not previously mentioned were obtained from Cell Signaling.

### Quantitative PCR

qPCR was performed using TaqMan probes from Applied Biosystems (Foster City, CA) as previously described [30].

### Cell culture experiments

Biotin hydrazide modification and streptavidin purification of carbonylated proteins: HepG2 (HB-8065, ATCC Manassas, VA) and RAW264.7 (TIB71, ATCC) cells were maintained at 50 to 80% confluence in RPMI 1640 medium supplemented with 10% fetal bovine serum, 100 mM HEPES, 100 IU/ml penicillin, and 100 g/ml streptomycin. Before each experiment, cells were plated at a density of 10^6^ cells per well in RPMI 1640 medium plus serum and allowed to adhere overnight. The following day, the cells were washed twice in serum-free RPMI 1640 medium and treated with 100μM 4-HNE in serum-free medium for 60 minutes. Cells were then lysed in 50 mM HEPES, 100 mM NaCl, 2 mM EDTA, 0.5% Triton-X100 and 5 mM biotin hydrazide and allowed to incubate for 60 min at room temperature on a rotating mixer. To remove excess biotin, lysates were dialyzed (Slidalyzer 3500mw cutoff Pierce Thermo Fisher) overnight at 4°C. Biotinylated proteins were purified by incubating with streptavidin agarose beads (Thermo Fisher) on a rotary mixer at 4°C O/N. The following morning, beads were washed 6X2 min in ice cold PBS, boiled for 5min in 5X SDS PAGE buffer, run on 15% SDS PAGE gels and transferred onto Polyvinylidine fluoride membranes for Western blotting.

### Statistical analysis

The data are presented as mean ± standard deviation (STDEV). Comparisons between two groups were performed by Student’s t-test. Statistical significance was set at P<0.05. Prism 5 for Windows (GraphPad Software, San Diego, CA) was used to perform all statistical tests.

## Results

### Evidence of autophagy in PSC liver

**Fig 1:**
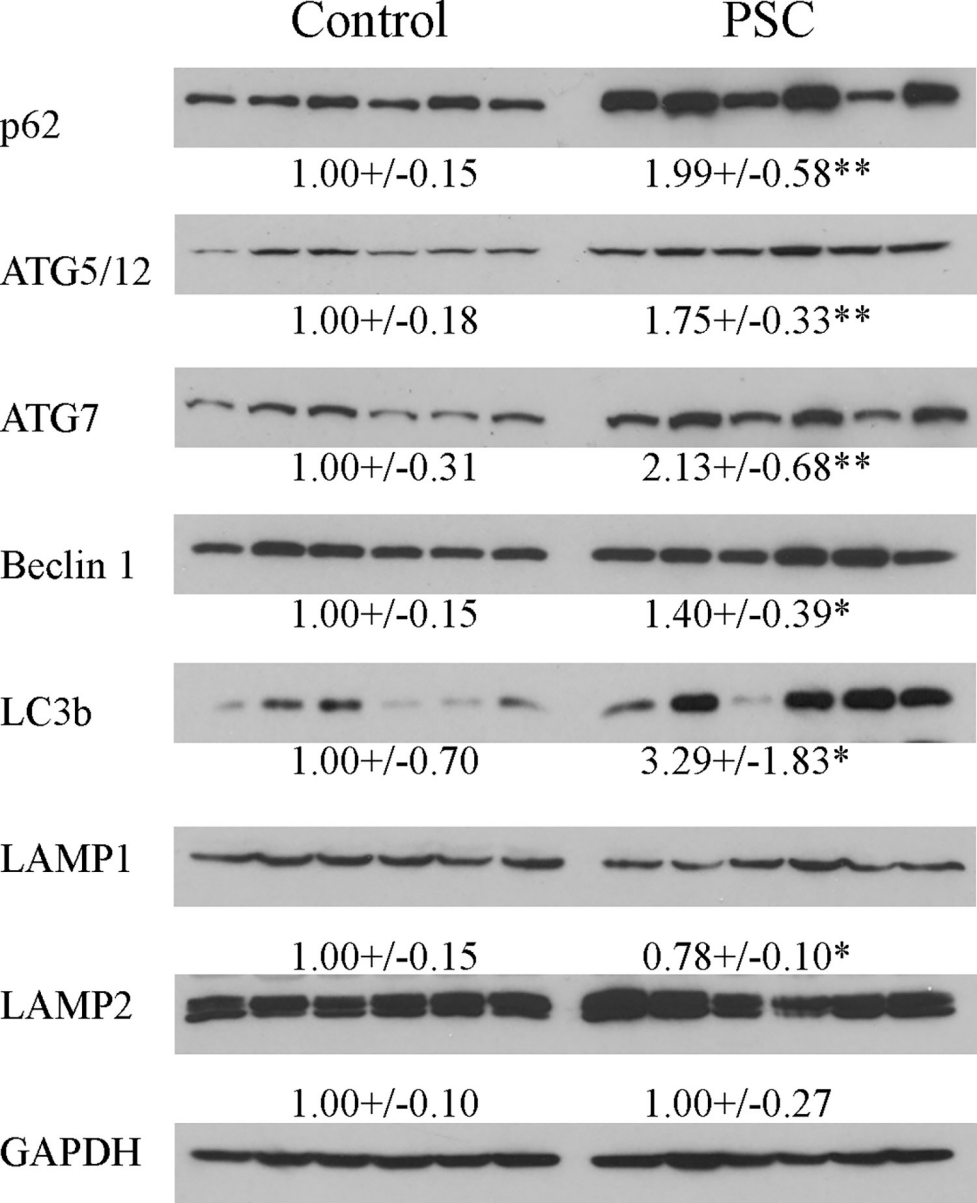
Increased expression of autophagic proteins in PSC. Western blotting of p62, ATG5/12, ATG7, Beclin1, LC3B, LAMP1 and LAMP2 in liver extracts prepared from patients with PSC and Controls. Blots were normalized to GAPDH expression. Data are presented as Mean ± STDEV and were statistically analyzed using Student’s t-test, *p<0.05, **p<0.01, (N = 6/group).

We have previously reported increased oxidative stress and dysregulation of Nrf2 target genes in human PSC liver and in the Mdr2^KO^ mouse model of chronic cholestasis [22, 26]. In those studies, elevated markers of oxidative stress as well as induction of antioxidant proteins was present primarily in parenchymal cells that were adjacent to areas of ongoing inflammation and fibrosis. Recent reports have linked the NRF2 antioxidant response to activation of autophagy [24, 25]. Previous reports have shown that there is a defect in autophagy in PSC and that expression of the autophagic proteins p62 and LC3 is increased in PSC [13, 14] as well as in BDL mice [12, 33]. First, to verify and further define cholestasis-dependent changes in autophagy proteins in our PSC samples, expression of p62, Beclin1, LC3a/b, ATG5/12, ATG7, LAMP1 and LAMP2 was evaluated by Western blotting using lysates prepared from PSC liver tissue (**Fig 1**). Compared to normal human liver, with the exception of LAMP1 which was decreased and LAMP2 which showed no difference, protein expression was significantly increased in all other autophagic proteins that were examined in PSC tissue.

### Evidence of autophagy in acute and chronic murine cholestasis

We next sought to examine the expression of autophagy in BDL mice, an acute cholestasis model. C57BL6/J mice (8–10 weeks old) were subjected to either sham or BDL surgery and after 3 days mice were sacrificed, and liver injury was assessed. Serum aspartate aminotransferase (AST), alanine aminotransferase (ALT), alkaline phosphatase, total bilirubin and serum bile acids were all significantly increased by BDL (**S1 Fig**). To determine extent of necrosis, fibrosis and the ductular reaction, tissue sections were stained with hematoxylin and eosin, picrosirius red (PSR) and immunohistochemically for cytokeratin 7 expression (**S2 Fig**). BDL induced necrosis on average in approximately 10% of hepatocytes, however there were no significant differences in PSR or cytokeratin 7 staining in BDL vs. sham mouse liver (**S3A–S3C Fig**). The lack of increased PSR staining in our BDL samples may have been secondary to the short-term nature of the BDL, unlike prior reports that BDL induces fibrosis [34]. Therefore, to further examine the effects of 3-day BDL on fibrosis pathways, mRNA expression of *Timp1* and *Col1a1* was assessed by qPCR (**S3D Fig**) which showed that both *Timp1* and *Col1a1* mRNA were significantly increased in the BDL group supporting induction of fibrogenesis genes.

**Fig 2:**
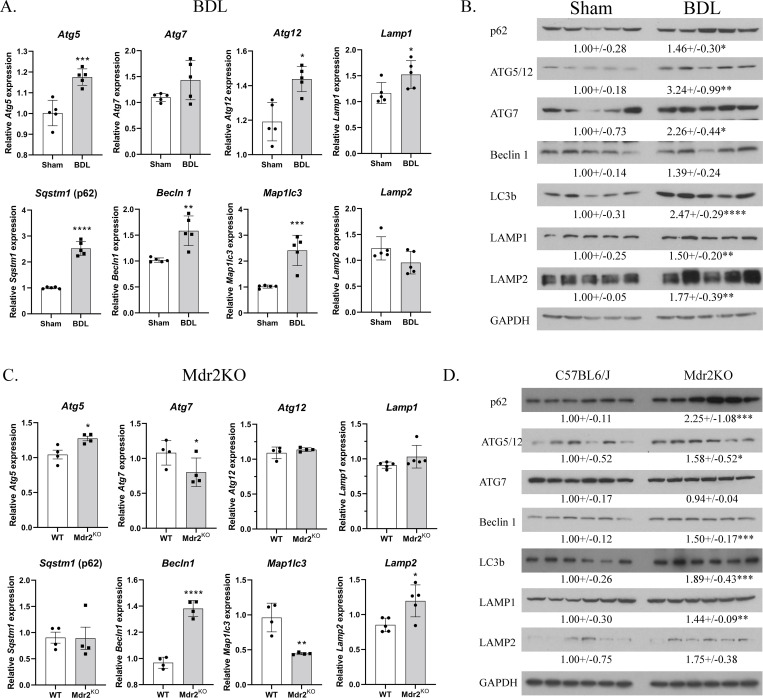
Increased expression of autophagic proteins in acute murine cholestasis. **A**. qPCR analysis of mRNA for autophagic proteins in liver tissue isolated from 3-day sham and BDL adult mice (N = 5/group). Expression was normalized against HPRT. **B**. Western analysis of autophagic proteins in liver tissue isolated from BDL mice (N = 5/group). Blots were normalized to GAPDH expression. **C**. qPCR analysis of mRNA for autophagic proteins in liver tissue isolated from 10-week old Mdr2^KO^ mice (N = 4/group). **D**. Western analysis of autophagic proteins in liver tissue isolated from 10 week on Mdr2^KO^ mice (N = 6/group). Blots were normalized to GAPDH expression Data are presented as Mean ± STDEV and were statistically analyzed using a Student’s t-test, *p<0.05, **p<0.01, ***P<0.001, ****p<0.0001, N = 6/group.

In murine BDL models of acute cholestatic injury, autophagy has been shown to be induced by upregulation of p62, Beclin 1 and LC3 expression [12, 33]. We next sought to more extensively characterize proteins that contribute to autophagy in the 3-day BDL model. Both *mRNA expression (****Fig 2A****) and protein expression (****Fig 2B****) of Atg5*, *Atg7*, *Atg12*, *Beclin 1*, *p62*, *LC3b*, and LAMP1 were significantly increased in BDL liver compared to sham controls. Interestingly, mRNA expression of LAMP2 was not different but protein expression was significantly increased. These data support both increased mRNA expression and accumulation of autophagosomal proteins in acute murine cholestasis.

In mice, BDL results in a severe acute cholestatic injury with high mortality that precludes evaluating chronic cholestasis in this model. In contrast, Abcb4^KO^ (Mdr2^KO^) mice develop a more gradual cholestatic injury and periductal liver fibrosis mirroring human PSC beginning at 3 weeks of life and progressing to florid hepatobiliary inflammation by 8–10 weeks [28, 35, 36]. Therefore, Mdr2^KO^ mice present a useful model to investigate mechanisms of chronic cholestatic liver injury. We have previously reported that expression of Nrf2 targets in Mdr2^KO^ mice was zonally dysregulated which corresponded to areas with evidence of oxidative stress [28]. However, autophagic processes have not been well characterized in Mdr2^KO^ mice. Using liver samples from our previous study, qPCR analysis showed that, of the autophagy genes that were upregulated in BDL mice, in Mdr2^KO^ mice only *Becln1*, *Atg5* and *Lamp2* expression was increased whereas expression of *Atg7* and *Map1lc3* was significantly decreased (**Fig 2C**). All other genes were not significantly different. Examining protein expression, p62, ATG5/12, Beclin 1, LC3b, LAMP1 and LAMP2 were all significantly increased but ATG7 was suppressed (**Fig 2D**). These data indicate that the accumulation of autophagosomal proteins in chronic murine cholestasis was accompanied by downregulation of mRNA expression of many of these genes.

### Spatial expression of autophagic proteins during cholestasis

**Fig 3:**
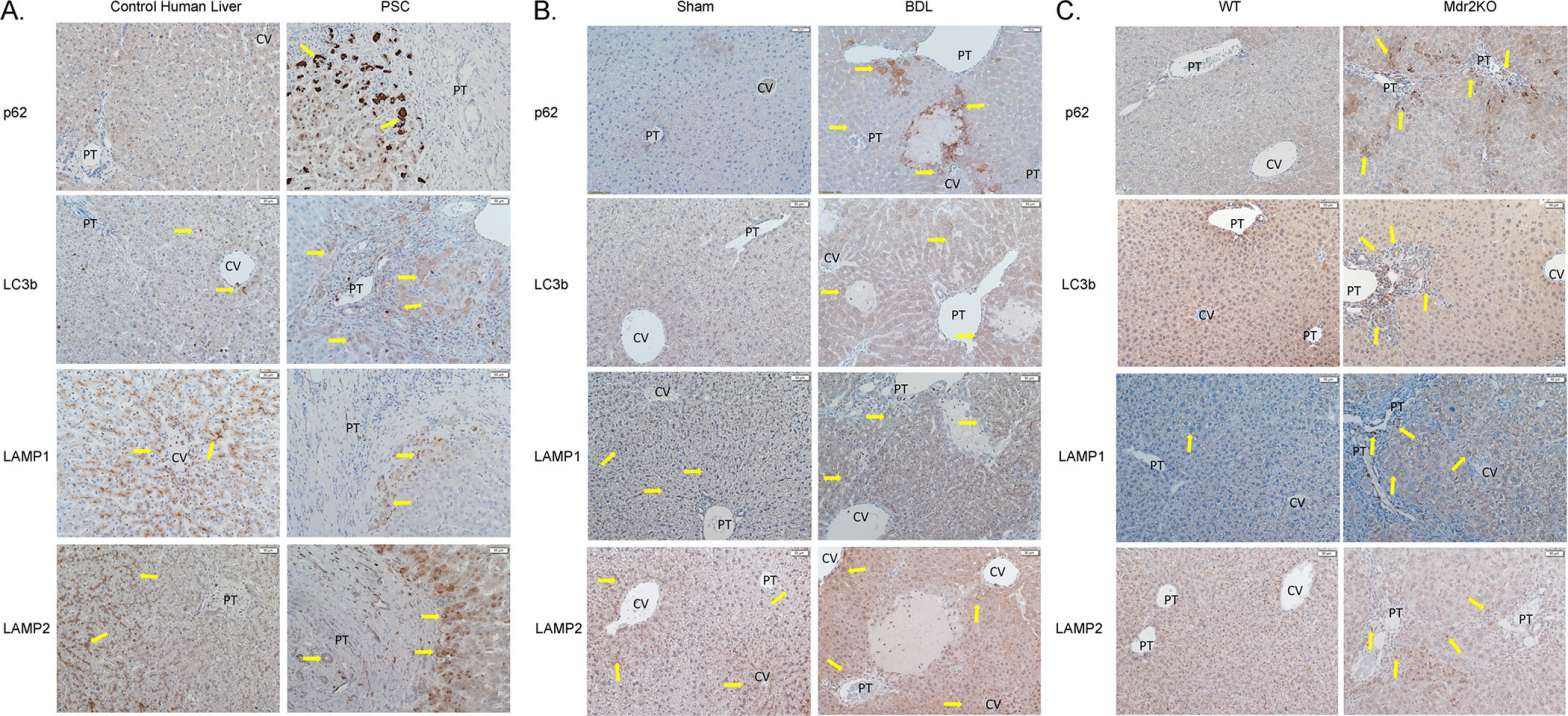
Increased accumulation of p62, LC3b, LAMP1 and LAMP2 in PSC and BDL mice is located primarily in parenchymal cells that surround the portal triad. Tissue sections from normal human liver, PSC, sham and BDL mice were examined for p62, LC3b, LAMP1 and LAMP2 expression. **A**. Human control and PSC. **B**. Sham and BDL. N = 3-5/group, 200X, CV-central vein, PT-portal triad. Arrows indicate increased staining. **C**. 10-week old WT and Mdr2^KO^ (200X). N = 3-5/group, 200X, CV-central vein, PT-portal triad. Arrows indicate increased staining.

To further understand the spatial relationships of autophagic proteins in both human and murine cholestasis, immunohistochemistry was performed using anti-p62, LC3b, LAMP1 and LAMP2 monoclonal/polyclonal antibodies. In human control tissue, weak staining of p62 was present in scattered periportal and centrilobular hepatocytes. In human PSC liver tissue, p62 staining was increased compared to control liver in periportal hepatocytes and in some inflammatory cells surrounding areas of fibrosis (**Fig 3A**, arrows). In control human tissue, LC3b expression was not evident in hepatocytes but was present in scattered inflammatory cells (arrows). In PSC liver, LC3b expression was increased in periportal hepatocytes (arrows). In human control tissue, LAMP1 expression was predominantly centrilobular with weak periportal staining. In PSC liver, LAMP1 expression was increased in hepatocytes surrounding areas of active fibrosis (arrows). LAMP2 expression was primarily around the central vein in control human tissue while in PSC liver, expression increased in periportal hepatocytes (arrows) surrounding fibrotic tissue. Examining mouse liver, weak p62 staining was evident in centrilobular hepatocytes in sham treated mouse liver (**Fig 3B**). Following 3-day BDL, prominent p62 staining was observed in hepatocytes surrounding areas of hepatocellular necrosis as well as around the central vein (arrows). LC3b was increased in periportal hepatocytes as well as hepatocytes surrounding areas of necrosis (arrows). In sham mice, faint LAMP1 staining was present in centrilobular hepatocytes with some expression in inflammatory cells (arrows). Following BDL, LAMP1 panlobular staining of hepatocytes was prominent (arrows). In sham liver, LAMP2 staining was present in centrilobular hepatocytes whereas following BDL, LAMP2 was increased in hepatocytes throughout the lobule (arrows). In the Mdr2^KO^ model of chronic cholestasis, p62 expression was prominent in periportal hepatocytes and cholangiocytes as well as in inflammatory cells (**Fig 3C** arrows). Interestingly, in comparison to p62 staining, LC3b expression was apparent in far fewer hepatocytes but was evident in cholangiocytes as well as in periportal inflammatory cells (**Fig 3C** arrows). Increased expression of both LAMP1 and LAMP2 was evident in periportal hepatocytes (**Fig 3C** arrows).

**Fig 4:**
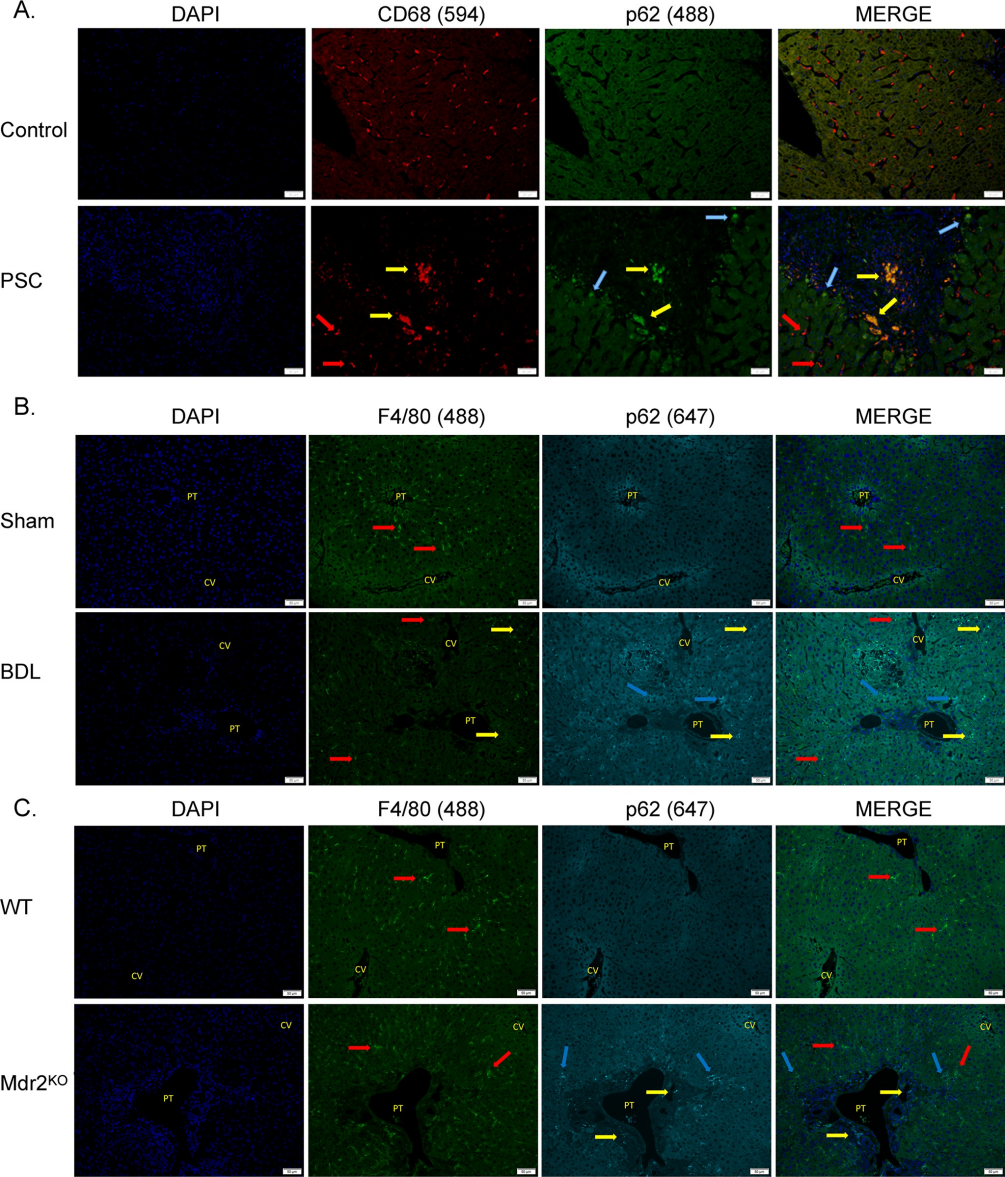
Colocalization of p62 in macrophages during cholestasis. **A.** Colocalization of p62 and CD68+ macrophages in human PSC. Paraffin embedded formalin fixed tissue sections from normal human PSC liver were analyzed immunohistochemically using a rabbit polyclonal antibody directed against p62, a mouse monoclonal antibody directed against the macrophage marker CD68 followed by FITC 488 conjugated anti-rabbit and Alexa Fluor 594-conjugated anti-mouse secondary antibodies. Figures are representative of hepatic tissue isolated from three Control and four PSC patients respectively, (200X). **B-C.** Colocalization of p62 and F4/80 positive macrophages in murine cholestasis. Paraffin embedded formalin fixed tissue sections from **B.** Sham, BDL liver **C.** WT, Mdr2^KO^ liver were analyzed immunohistochemically using a rabbit polyclonal antibody directed against p62, a rat polyclonal antibody directed against the macrophage marker F4/80 followed by FITC 647 conjugated anti-rabbit and Alexa Fluor 488-conjugated anti-rat secondary antibodies. Slides were examined using fluorescent microscopy (Blue arrows = p62 positive hepatocytes, red arrows = macrophages, yellow arrows-colocalization), nuclei were visualized by DAPI. Figures are representative of hepatic tissue from three each of sham, WT and the BDL, Mdr2^KO^ animals, respectively (200X).

To gain further insight into the specific type of inflammatory cells in PSC liver that accumulated p62 (Fig 3A), fluorescent IHC was performed using rabbit polyclonal anti-p62 and mouse monoclonal anti-CD68 antibodies (staining macrophages). In control human liver, scattered CD68 positive cells (red) were evident across the hepatic lobule but p62 staining (green) was not present in these cells. (**Fig 4A**). In contrast in PSC liver, colocalization revealed 2 distinct subpopulations of CD68 positive macrophages. Macrophages that were located within the sinusoids away from the portal tracts stained only with CD68 (red arrows) and did not exhibit p62 staining (CD68^+^/p62^-^). Within the portal triad, colocalization of p62 and CD68 (CD68^+^/p62^+^, yellow arrows) was identified in a subset of infiltrating macrophages. To determine if colocalization was similarly present in the mouse models, p62 and F4/80 staining of macrophages was examined. In both the BDL and Mdr2^KO^ groups, 2 distinct macrophage populations were also observed (**Fig 4B and 4C**). Macrophages located away from the portal triad stained only with F4/80 (F4/80^+^/p62^-^; red arrows) contrasting with scattered macrophages within the portal tracts that were positive for both stains (F4/80^+^/p62^+^ macrophages; yellow arrows).

### Oxidative injury in BDL mice

**Fig 5:**
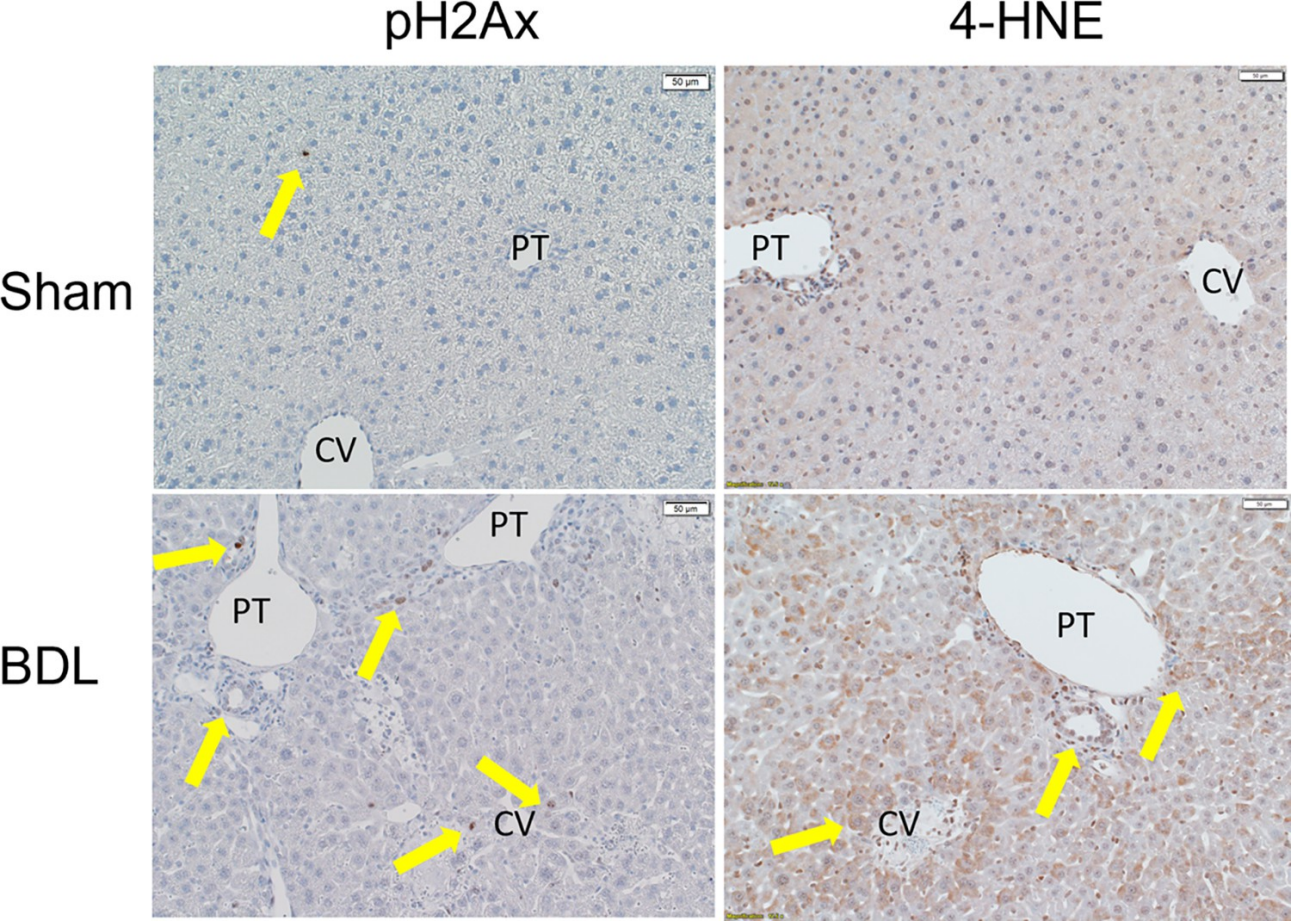
Increased periportal oxidative injury in acute cholestasis. Tissue sections from sham and BDL mice were probed immunohistochemically for pH2Ax and 4-HNE. N = 4/group, 200X, PT-portal triad, CV-Central vein. Arrows indicate area of increased staining.

Oxidative stress is a key mediator of hepatic injury during cholestasis. Accumulation of products of lipid peroxidation has been shown to occur as early as 2 days post BDL [37]. We have shown that increased periportal expression of antioxidant response proteins are present in human PSC liver [22, 26]. Furthermore, accumulation of proteins that are post-translationally modified by 4-HNE correlates with increased expression of antioxidant proteins in Mdr2^KO^ mice [28]. Concurrently, in Mdr2^KO^ mice, staining for phosphorylated Serine 129 γHistone 2Ax (H2Ax, a marker of oxidative DNA damage [38]) was increased in periportal hepatocytes and inflammatory cells. Thus, there is compelling evidence of oxidative modification of lipids, proteins and DNA in human and mouse cholestasis. We next sought to determine the spatial representation in the liver lobule of evidence of oxidative injury during cholestasis. We analyzed oxidative DNA damage and protein carbonylation in tissue sections from sham and 3-day BDL mice by staining for H2Ax and 4-HNE (**Fig 5**). Following 3-day BDL, a significant increase in H2Ax-positive hepatocytes and inflammatory cells were observed within and surrounding areas of hepatocellular necrosis as well as in cholangiocytes (**Fig 5 (arrows), S3E Fig**). We then assessed the percent H2Ax+ nuclei with respect to cell type (inflammatory cells, hepatocytes and cholangiocytes). In the BDL livers, 44% of the total H2Ax positive cells were macrophages that were predominantly in the portal triad or around areas of necrosis, 36% were hepatocytes (periportal and surrounding areas of necrosis) and 20% were cholangiocytes. Examining protein carbonylation, only weak 4-HNE staining was present in hepatocytes around the central vein in Sham liver. Following 3-day BDL, 4-HNE positive hepatocytes were abundant around areas of active necrosis as well as in the layer of hepatocytes surrounding the portal triad. Taken together, these data indicate increased oxidative injury following BDL in areas of necrosis and surrounding the portal tracts.

### Nrf2 expression in cholestasis

**Fig 6:**
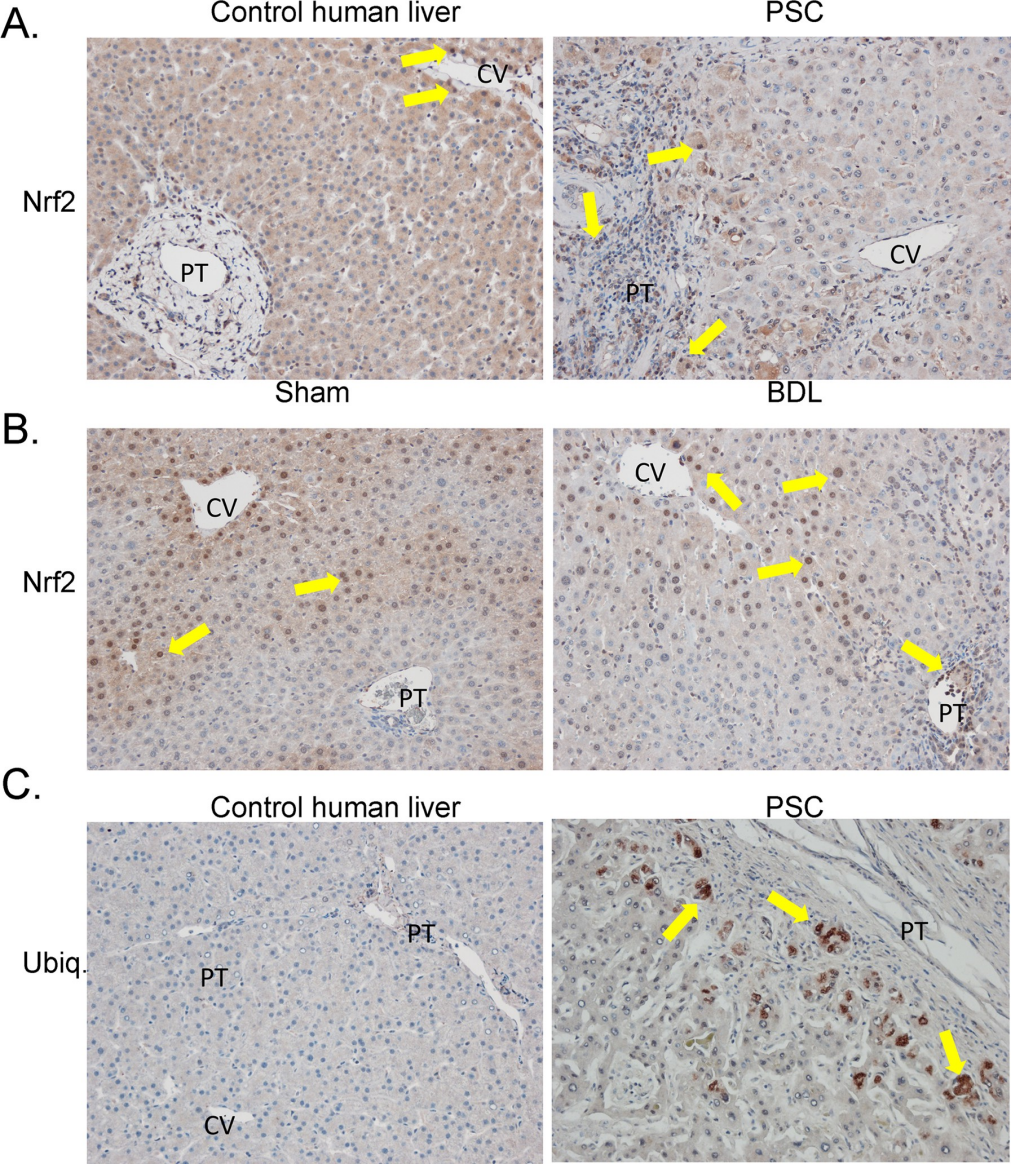
Increased periportal nuclear localization of Nrf2 in human and murine cholestasis. Tissue sections were examined for Nrf2 nuclear localization **A**. Normal human liver, PSC (200X). **B**. Sham mice and BDL mice (100X). **C**. Accumulation of ubiquitinated proteins in parenchymal cells surrounding the injured portal triad in PSC. n = 3-4/group, (200X). CV-central vein, PT-portal triad. Arrows indicate increased periportal staining.

Previous reports have shown that Nrf2 is upregulated following BDL and that constitutive activation of Nrf2 is protective of liver injury [37, 39, 40]. To determine the effects of cholestasis on Nrf2, localization of Nrf2 was examined by immunohistochemistry in human PSC and murine BDL livers (**Fig 6A and 6B**). In normal human liver, Nrf2 expression was primarily in the cytosol with some positively stained nuclei of hepatocytes. In PSC, cytosolic staining was notably decreased across the lobule and nuclear staining was evident. In normal mice, Nrf2 staining was present in hepatocytes around the central vein with some nuclear localization present (**Fig 6A**, Arrows). In BDL mice, cytosolic staining decreased in centrilobular hepatocytes with a zone-specific increase in nuclear staining of periportal hepatocytes. Combined, these data support activation of Nrf2 with translocation to the nucleus in periportal hepatocytes in both murine and human cholestatic liver disease. Recent reports have shown that there is an accumulation of ubiquitinated proteins in the liver following BDL [12]. We hypothesized that a similar process may be occurring in human PSC. Compared to control human liver, in PSC there was increased staining of ubiquitinated proteins in periportal hepatocytes that surrounded areas of increased inflammation and fibrosis (**Fig 6C**). Taken together, these data demonstrate increased hepatic Nrf2 activation and accumulation of ubiquitylated proteins in periportal hepatocytes in both human and murine cholestasis.

### Impact of acute cholestasis on Nrf2 antioxidant responses

**Fig 7:**
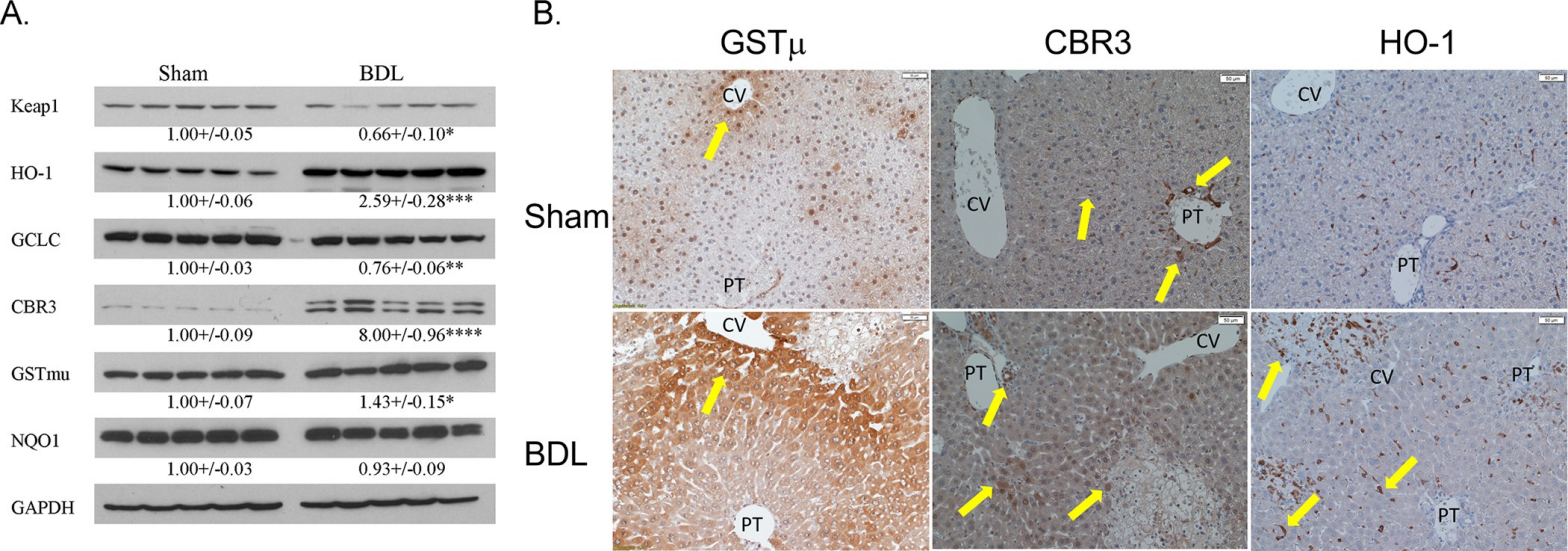
Increased periportal oxidative stress and upregulation of Nrf2 antioxidant proteins in acute cholestasis. **A**. Western analysis of selected Nrf2 target proteins (HO-1, GCLC, CBR3, GSTμ, NQO1) in liver tissue isolated from sham and BDL mice. Blots were normalized to GAPDH expression. Data are presented as Mean ± SEM and were statistically analyzed using a student’s t-test, *p<0.05, **p<0.01, ***P<0.001, ****p<0.0001, N = 5/group. **B**. Tissue sections from sham and BDL mice were probed immunohistochemically for GSTμ, Cbr3 and HO-1. N = 4/group, 200X, PT-portal triad, CV-Central vein. Arrows indicate area of increased staining.

We have previously shown that expression of Nrf2-regulated antioxidant genes, including glutamate-cysteine ligase catalytic subunit (GCLC), heme oxygenase (HO-1), and glutathione S-transferase μ (GSTμ), were suppressed in human chronic cholestasis in end-stage human PSC liver samples [22]. Data in Fig 6 supports Nrf2 activation during cholestasis. To determine if oxidative stress similarly impacted cellular antioxidant responses in an acute cholestasis model, the expression in 3-day BDL mouse liver of the Nrf2 regulatory and response proteins Kelch-like ECH-associated protein 1 (Keap1), GCLC, HO-1, GSTμ and carbonyl reductase 3 (Cbr3) was examined and quantified by Western blot analysis (**Fig 7A**). Following 3-day BDL, protein expression of HO-1, CBR3 and GSTμ were increased whereas Keap1 and GCLC decreased. To explore the spatial expression of Nrf2 proteins in liver during acute cholestasis, IHC was performed for GSTμ, CBR3, and HO-1 (**Fig 7B**). In sham controls, expression of GSTμ was evident in the nucleus and the cytosol of hepatocytes around the central vein. BDL resulted in stronger staining of GSTμ across the liver lobule. In sham controls, Cbr3 expression occurred primarily in cholangiocytes with some staining of macrophages (arrows). Following BDL, Cbr3 staining increased in scattered hepatocytes as well as well as in cholangiocytes (arrows) but not in macrophages. In hepatic macrophages, HO-1 is a canonical gene target of Nrf2. In sham controls, panlobular staining of HO-1 was present in macrophages within the hepatic sinusoids. Following BDL, staining of HO-1 positive inflammatory cells was increased within necrotic areas as well as in macrophages throughout the hepatic lobule. In summary, in contrast to Mdr2^KO^ which induces a periportal antioxidant response [28], BDL induces expression of antioxidant proteins primarily in the centrilobular region as well as in hepatocytes surrounding areas of necrosis.

### Colocalization of oxidative stress and autophagy

**Fig 8:**
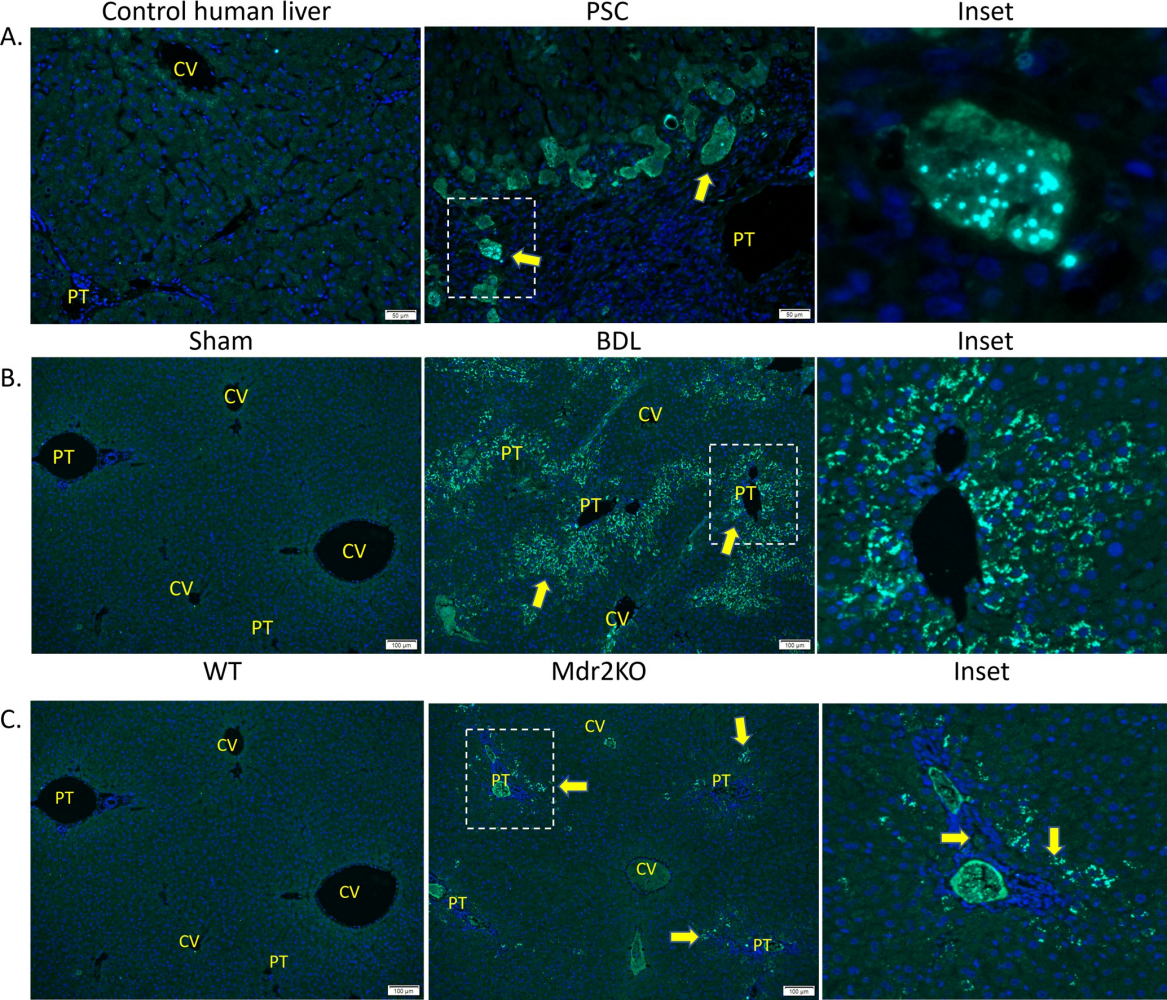
Colocalization of p62 and 4-HNE in liver in cholestasis **A.** Control and PSC liver (200X). **B.** Sham and BDL cholestatic liver (100X) C. WT and Mdr2^KO^ liver (100X). Arrows indicate periportal hepatocytes of increased staining/colocalization. n = 4/group, Blue-Dapi, Green-4-HNE, Cyan-p62. CV-central vein, PT-portal triad.

We have shown that both evidence of oxidative injury and autophagy are present in periportal hepatocytes during human and murine cholestasis. To determine if both processes are occurring in the same cell, fluorescent IHC was used (**Fig 8**). In normal human liver there was very little staining of either p62 or 4-HNE. In PSC cholestatic liver, however, increased staining of both p62 (cyan) and 4-HNE (green) were co-localized in hepatocytes surrounding the portal triad. This pattern of co-localization was also replicated in 3 day-BDL mice (**Fig 8**). Examining the human liver fluorescent microscopy at higher magnification, we observed that numerous autophagosomes accumulated in the hepatocytes which correspond with increased 4-HNE staining in PSC liver. Furthermore, there was no accumulation of p62 in hepatocytes that do not demonstrate significant 4-HNE staining. These observations support the conclusion that increased oxidative stress was associated with the induction (as evidenced by the upregulation of autophagic proteins) and the inhibition (as evidenced by the accumulation of p62) of autophagy during human and murine cholestatic liver disease.

### p62 is a target of oxidative modification through carbonylation in human PSC

**Fig 9:**
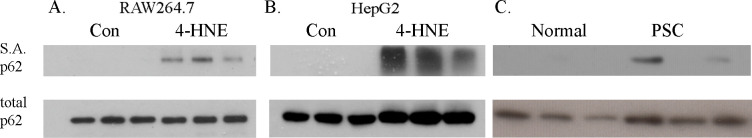
p62 is a target of reactive aldehydes in *in vitro* cell culture and human PSC. Cells were treated with 100μM 4-HNE for 60 min. Cells were lysed, modified proteins were biotinylated using biotin hydrazide and carbonylated proteins purified by streptavidin bead pulldown. Carbonylation of p62 was assessed by Western blotting as described in methods. **A**. RAW264.7, **B**. HepG2. **C**. Carbonylated proteins from control and PSC liver extracts were treated with biotin hydrazide (5mM/60 min) followed by streptavidin purification and p62 Western analysis. N = 3/condition.

Our in vivo studies demonstrate the accumulation of p62 in periportal hepatocytes and a select set of macrophages and suggest that during cholestasis there is a defective autophagy within hepatocytes as well as some macrophages surrounding portal tracts and areas of increased inflammation and fibrosis. Recent reports demonstrate carbonylation of autophagic proteins in primary neurons treated with 4-HNE [41]. To determine if p62 was similarly a target of carbonylation in hepatocytes and macrophages, cultured HepG2 and RAW264.7 cells were treated with 4-HNE (100μM, x60 min), cells were lysed and carbonylated proteins purified using biotin hydrazide/streptavidin purification [42]. Treatment of both RAW264.7 and HepG2 cells with 100μM 4-HNE for 1 hr resulted in increased carbonylation of p62 (**Fig 9A and 9B**). The results showed increased carbonylation of p62 in both cell types (**Fig 9A and 9B**), validating that p62 is a target of reactive aldehydes in cell culture. To determine if p62 is a target in vivo in human disease, carbonylated proteins were purified from normal and PSC liver tissue and p62 carbonylation assessed by Western blotting [28, 43]. p62 carbonylation was present in 2 of 3 PSC liver samples available but was absent in 3 samples of normal human liver (**Fig 9C**). These data show that carbonylation of autophagosomal proteins occurs in human cholestatic liver disease.

To further explore the mechanism of p62 carbonylation, recombinant p62 (amino acids 85–442) was treated with 4-HNE (1:10 molar ratio/60 min) and carbonylation of specific amino acids assessed using LC-MS/MS. From the LC-MS/MS analysis, 4-HNE modification was detected on His^123^, Cys^128^, His^174^, His^181^, Lys^238^, Cys^290^, His^340^, Lys^341^ and His^385^ (**Table 2**).

**Table 2:** 4-HNE modified peptides identified from tryptic digests of 4-HNE-treated recombinant p62.

Peptide	Mass	m/z	RT	PTM
NM(+15.99)VH(+156.12)PNVIC(+57.02)DGC(+57.02)NGPVVGTR	2367.113	790.0453	19.18	Oxidation (M); 4-hydroxynonenal (HNE); Carbamidomethylation
EAALYPH(+156.12)LPPEADPR	1830.952	611.3234	22.43	4-hydroxynonenal (HNE)
LAFPSPFGHLSEGFSH(+156.12)S	1971.973	494.0058	20.75	4-hydroxynonenal (HNE)
NMVH(+156.12)PNVIC(+57.02)DGC(+57.02)NGPVVGTR	2351.118	784.7124	20.17	4-hydroxynonenal (HNE); Carbamidomethylation
NM(+15.99)VH(+156.12)PNVIC(+57.02)DGCNGPVVGTR	2310.092	771.0379	19.91	Oxidation (M) 4-hydroxynonenal (HNE)
SSSQPSSC(+156.12)CSDPSKPGGN(+.98)VEGATQSLAEQM(+15.99)R	3297.444	825.3683	15.08	Oxidation (M) 4-hydroxynonenal (HNE)
AGEARPGPTAESASGPSEDPSVNFLK(+156.12)	2726.34	682.5891	16.11	4-hydroxynonenal (HNE)
NMVH(+156.12)PNVIC(+57.02)DGC(+57.02)N(+.98)GPVVGTR	2352.103	785.042	21.58	4-hydroxynonenal (HNE); Carbamidomethylation; Deamidation (NQ)
IALESEGRPEEQM(+15.99)ESDNC(+57.02)SGGDDDWTH(+156.12)LS	3435.436	859.86	16.19	Oxidation (M); Carbamidomethylation; 4-hydroxynonenal (HNE)
NM(+15.99)VHPNVIC(+57.02)DGC(+156.12)NGPVVGTR	2310.092	771.0362	20.3	Oxidation (M) 4-hydroxynonenal (HNE)
LAFPSPFGH(+156.12)LSEGFSHSR	2128.074	710.3661	23.84	4-hydroxynonenal (HNE)
IALESEGRPEEQMESDNCSGGDDDWTHLSSK(+138.10)EVDPSTGELQSLQM(+15.99)PESEGPSSLDPSQEGPTGLK	7083.15	1417.653	22.37	Schiff 4 HNE; Oxidation (M)
N(+.98)MVH(+156.12)PNVICDGCN(+.98)GPVVGTR	2239.044	747.3508	15.58	4-hydroxynonenal (HNE)
NM(+15.99)VHPNVIC(+156.12)DGC(+57.02)N(+.98)GPVVGTR	2311.076	771.3643	20.93	Oxidation (M) 4-hydroxynonenal (HNE)
LYPH(+156.12)LPPEADPR	1559.835	520.9527	19.99	4-hydroxynonenal (HNE)
NM(+15.99)VHPNVIC(+156.12)DGC(+57.02)NGPVVGTR	2310.092	771.0397	20.31	Oxidation (M) 4-hydroxynonenal (HNE)
EAALYPH(+156.12)LPPEAD	1577.798	789.9064	24.51	4-hydroxynonenal (HNE)
IALESEGRPEEQMESDN(+.98)C(+57.02)SGGDDDWTH(+156.12)LSS	3507.457	877.8684	16.03	Carbamidomethylation; 4-hydroxynonenal (HNE)
SSQ(+.98)PSSC(+57.02)C(+156.12)SDPSKPGGN(+.98)VEGATQSLAEQMR	3252.422	814.1088	17.07	Deamidation (NQ) 4-hydroxynonenal (HNE)

Twenty micrograms of recombinant p62 was treated with 4-HNE (molar ratio of 1:10) for 60 min, digested with trypsin, and analyzed using LC-MS/MS as previously described [28, 44]. 4-HNE adducted residues are denoted in red (N = 3). RT = Retention time, m/z = mass/charge ratio, PTM = post translational modification.

## Discussion

Recent experimental evidence has shown that reducing oxidative stress has protective effects on cholestatic liver injury [37, 45, 46]. In the present study, we examined the impact of cholestasis on expression of Nrf2-dependent antioxidant genes and induction of autophagy. We found that some but not all Nrf2 target genes are upregulated following BDL. Furthermore, we observed that autophagy and proteins that are post-translationally modified by reactive aldehydes do indeed colocalize in periportal hepatocytes during cholestasis. In addition, for the first time, we show that the autophagosomal regulatory protein p62 is carbonylated in human cholestasis supporting a mechanism of inhibition of autophagy by oxidative modification of key proteins through carbonylation.

In the liver, under normal conditions, autophagy regulates protein and organelle turnover. During BDL, there is induction of autophagy most likely as a protective response, as evidenced by increased expression of autophagic proteins and formation of autophagosomes [16]. Thus, inhibition of autophagy has the potential to further exacerbate BDL-injury and, conversely, autophagy induction by rapamycin reduces oxidative stress and injury in BDL livers. These data support the hypothesis that cholestasis induces autophagy, but it is not sufficient alone to ameliorate hepatic injury and repair damaged hepatocytes [16]. Recent studies have shown that this is in part due to suppression of autophagic flux by elevated concentrations of bile acids which result in reduced degradation of autophagosomes [11, 12]. Accumulation of p62 is linked to defective autophagy, as demonstrated in mice with genetic ablation of Atg5 (Atg5^KO^) or Atg7 (Atg7^KO^) and defective autophagy which have increased accumulation of p62 [17]. Our data support the notion that cholestasis induces expression of autophagic genes, but in contrast to the report of Kim et al. [12], we show that Beclin 1 expression is increased at both the mRNA and protein level. This indicates that in cholestatic hepatocytes, induction of autophagy is constant regardless of the ability of the cell to remove autophagosomes. Furthermore, mRNA expression of LAMP2 is not changed but there is increased protein accumulation (Western blotting) and staining in the BDL tissue. Recently, it has been suggested that during acute cholestasis, autophagy is activated but during chronic cholestasis autophagy is inhibited [47]. We found that in acute BDL mice, with the exception of LAMP2, there is upregulation mRNA expression of autophagic genes as well as accumulation of proteins supporting both processes. Concurrently there still is accumulation of 4-HNE modified proteins as well as DNA damage as evidenced by H2Ax staining suggesting that autophagic degradation or Nrf2 activation is either not sufficient or is inhibited.

In the Mdr2^KO^ model, mRNA expression of autophagic genes (with the exception of *Becln1*, *Atg5* and *Lamp2*) were either unchanged or were significantly suppressed whereas protein accumulation was still present. This supports the hypothesis that autophagy is initiated in early cholestasis, but mRNA expression is downregulated in chronic cholestasis. Furthermore, both the BDL group and Mdr2^KO^ group have accumulation of autophagic proteins and possess increased 4-HNE and p62 staining in hepatocytes in the periportal region supporting the notion that accumulation of 4HNE modified proteins contributes to initiation of autophagy or is a direct result of inhibition of autophagosomal processing.

Zonally, we found that in PSC, accumulation of autophagosomes occurs primarily in zone 1 adjacent to the portal triad and areas of active inflammation and fibrosis. As interesting, we found colocalization of CD68 positive macrophages with p62 in the periportal region but not in centrilobular macrophages supporting recent single cell RNA sequencing data identifying multiple populations of macrophages in human cholestatic liver disease [48]. While we find that autophagy is primarily upregulated in the periportal region in the Mdr2^KO^ model as well as in livers from PSC patients, staining for p62 in the periportal hepatocytes in the BDL mice (zone 1) is also accompanied by p62 around areas of confluent hepatocellular necrosis (bile infarcts) which are located in zones 2 and 3 of the lobule.

Oxidative stress and autophagy are intricately linked. During chronic inflammation, cysteines on Keap1 are oxidized resulting in dissociation from Nrf2 and Nrf2 activation. p62 competes with Nrf2 for binding to Keap1. Once Keap1 dissociates from Nrf2, it can then bind with p62 and induce autophagy. Our previous data support the activation of antioxidant responses in periportal hepatocytes during cholestatic injury and we now show that expression of both p62 and Nrf2 activation is also present in periportal hepatocytes during cholestasis. Furthermore, expression of the Nrf2 target Cbr3 is only mildly upregulated following BDL, and HO-1 staining is only present in inflammatory cells. Expression of GSTμ however, is dramatically upregulated. This finding, combined with previous reports, suggests that despite increased nuclear Nrf2 expression, the Nrf2 response is not sufficient to mitigate oxidative injury. Although data are lacking at early time points, studies have shown that at 14d post BDL, Nrf2 nuclear localization is increased but Nrf2 binding to the promoter of glutathione cysteinyl synthase (GCLC) is decreased resulting in reduced concentrations of glutathione which in theory would make hepatocytes more susceptible to oxidative injury [39]. Our data suggest that suppression of Nrf2 mediated GCLC expression is also occurring at early stages (3 days) of BDL-induced cholestasis. Furthermore, concurrent localization of carbonylated proteins in hepatocytes that accumulate p62 suggests that the mechanism of autophagy induction during cholestasis is in part due to Keap1-mediated activation of p62 even though Nrf2 is not activating all of its targets.

4-HNE specifically targets cysteine, lysine and histidine residues on proteins [18]. Previously we have determined that carbonylation of other proteins including Akt and PTEN is inhibitory towards enzymatic activity [42, 49]. In human as well as murine cholestatic tissue, using mass spectrometry and global carbonylomics, we have shown that a variety of vesicular trafficking and autophagic proteins including Rab GTPases as well as LAMP1/2, are carbonylated [28]. p62 possesses critical lysine residues that can either promote autophagosome formation and degradation or inhibit it through conjugation with ubiquitin [50, 51]. In our *in vitro* carbonylation analysis, we identified 4-HNE modification on Lys^238^ and Lys^341^ in p62. Interestingly, none of the lysine residues identified *in vitro* have been shown to be sites of ubiquitination but the recombinant protein that was used did not have the first 84 residues of p62. Although the impact of carbonylation of the specific amino acids of p62 are unknown, we postulate that modification of p62 by 4-HNE would in theory negatively impact autophagosomal processing and that the combined effect of inability to detoxify oxidative species (reduced Nrf2 responses) along with the inhibition of autophagic flux ultimately results in lipid peroxidation and accumulation of carbonylated proteins. This is supported by the lack of accumulation of p62 in hepatocytes that do not demonstrate significant 4-HNE staining. Importantly, experiments herein do not effectively answer whether formation and accumulation of carbonylated proteins induce autophagy or if the accumulation of carbonylated proteins is a result of inhibition of autophagy. Future studies will be necessary to specifically address the cell specific impact of carbonylation on autophagy and autophagolysosomal degradation. Overall, it can be concluded that increased oxidative stress and the resulting accumulation of carbonylated proteins may be a result of defective autophagosomal processing during human and murine cholestatic liver disease. The specific location of the affected hepatocytes (periportal hepatocytes) in human and murine cholestasis is of particular interest in that these cells have been shown to be capable of trans-differentiation into biliary epithelium that may be involved in the regeneration and healing of cholestatic bile duct injury [19, 20, 52], as seen in PSC. This raises the possibility that defective autophagy as a result of oxidative stress may render these cells incapable of transdifferentiation, thus perpetuating intrahepatic bile ductular injury, and that reduction of oxidative stress may promote autophagy of the periportal hepatocytes allowing for transdifferentiation and bile duct healing. Testing this hypothesis will require future experimental approaches.

## Supporting information

S1 Checklist (PDF)

S1 Fig Serum biochemical analysis of liver injury in WT and BDL treated mice. Aspartate aminotransferase (AST), alanine aminotransferase (ALT), alkaline phosphatase, total bilirubin and total serum bile acid concentrations. Data were analyzed statistically using a Student’s t-test and are presented as Mean ± STDEV, ***p<0.0001, n = 5/group.
(TIF)

S2 Fig Histochemical assessment of liver injury and ductular proliferation in C57BL/6 sham and 3-day BDL mice. **A.** Hematoxylin and Eosin (H&E) 200X. **B**. Picrosirius Red (PSR) 100X. **C**. Cytokeratin 7 (CK7) 200X. CV-central vein, PT-portal triad, NEC-necrosis, n = 4/group.
(TIF)

S3 Fig Quantification of necrosis, fibrosis, H2Ax positive nuclei and the ductular reaction (CK7), in liver tissue from C57BL/6 sham and 3-day BDL mice. **A**. Percent necrotic injury. **B**. Quantification of Picrosirius red staining (PSR). **C**. Percent cytokeratin 7 (CK7) positive cells. **D**. qPCR analysis of mRNA for fibrogenic genes *Timp1* and *Col1a1* in liver tissue from control and 3D BDL mouse liver (N = 4/group). **E.** H2Ax positive nuclei/100X field. Data were statistically analyzed using Student’s t-test and are presented as Mean ± STDEV. ****p<0.0001, n = 4/group.
(TIF)

S1 Raw images (PDF)

## Abbreviations

4-HNE: 4-hydroxynonenal
ALT: Alanine aminotransferase
AST: Aspartate aminotransferase
Atg: autophagy related
BDL: bile duct ligation
Cbr3: Carbonyl reductase 3
Gclc: Glutathione cysteinyl ligase catalytic subunit
GST: Glutathione S-transferase
HO-1: Heme oxygenase 1
Nrf2: Nuclear factor erythroid 2-related factor 2
PSR: picrosirius red, PSC; Primary Sclerosing Cholangitis
qPCR: quantitative PCR
