# Supplementary figures and images for: The autophagic protein p62 is a target of reactive aldehydes in human and murine cholestatic liver disease
Source: PLoS One. 2022 Nov 15;17(11):e0276879. doi: 10.1371/journal.pone.0276879 (PMC9665405; doi:10.1371/journal.pone.0276879)

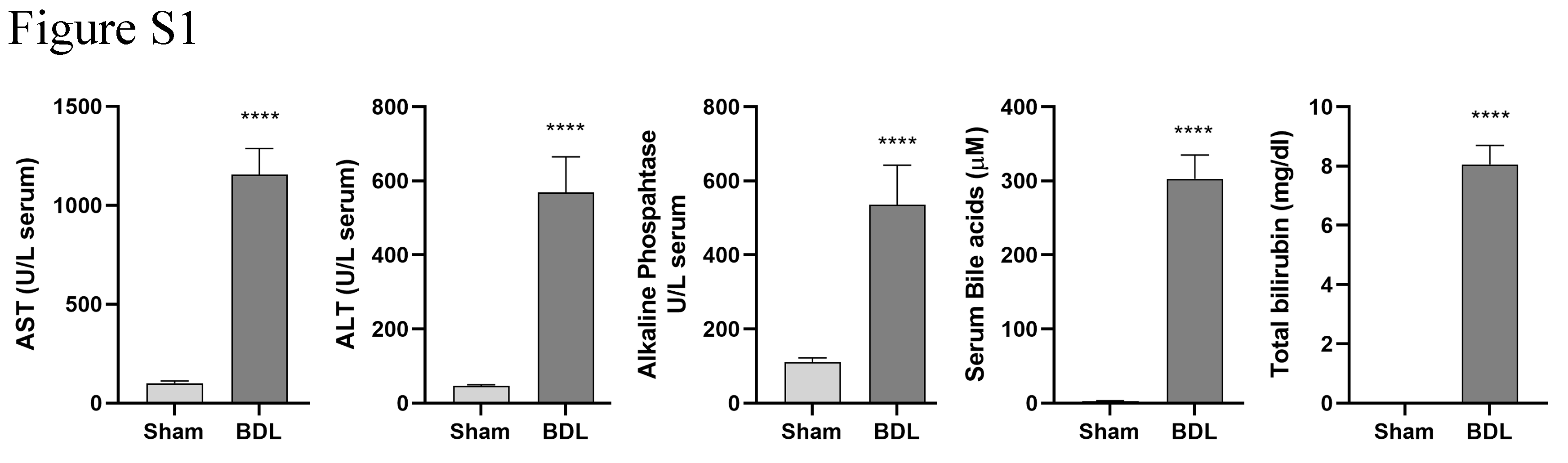

Supplement: S1 Fig — Aspartate aminotransferase (AST), alanine aminotransferase (ALT), alkaline phosphatase, total bilirubin and total serum bile acid concentrations. Data were analyzed statistically using a Student’s t-test and are presented as Mean ± STDEV, ***p<0.0001, n = 5/group. (TIF) [file pone.0276879.s002.tif]

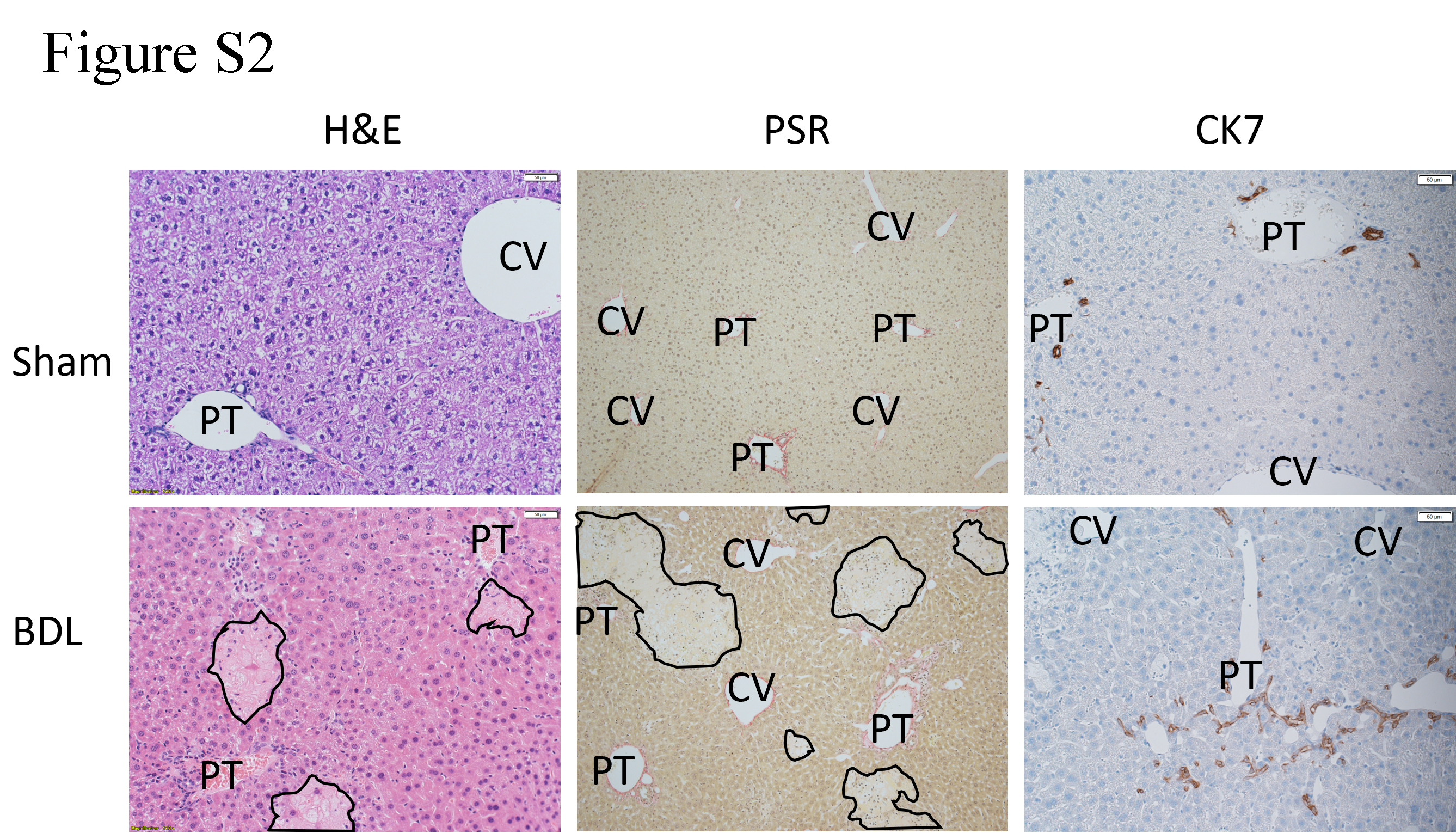

Supplement: S2 Fig — A. Hematoxylin and Eosin (H&E) 200X. B. Picrosirius Red (PSR) 100X. C. Cytokeratin 7 (CK7) 200X. CV-central vein, PT-portal triad, NEC-necrosis, n = 4/group. (TIF) [file pone.0276879.s003.tif]

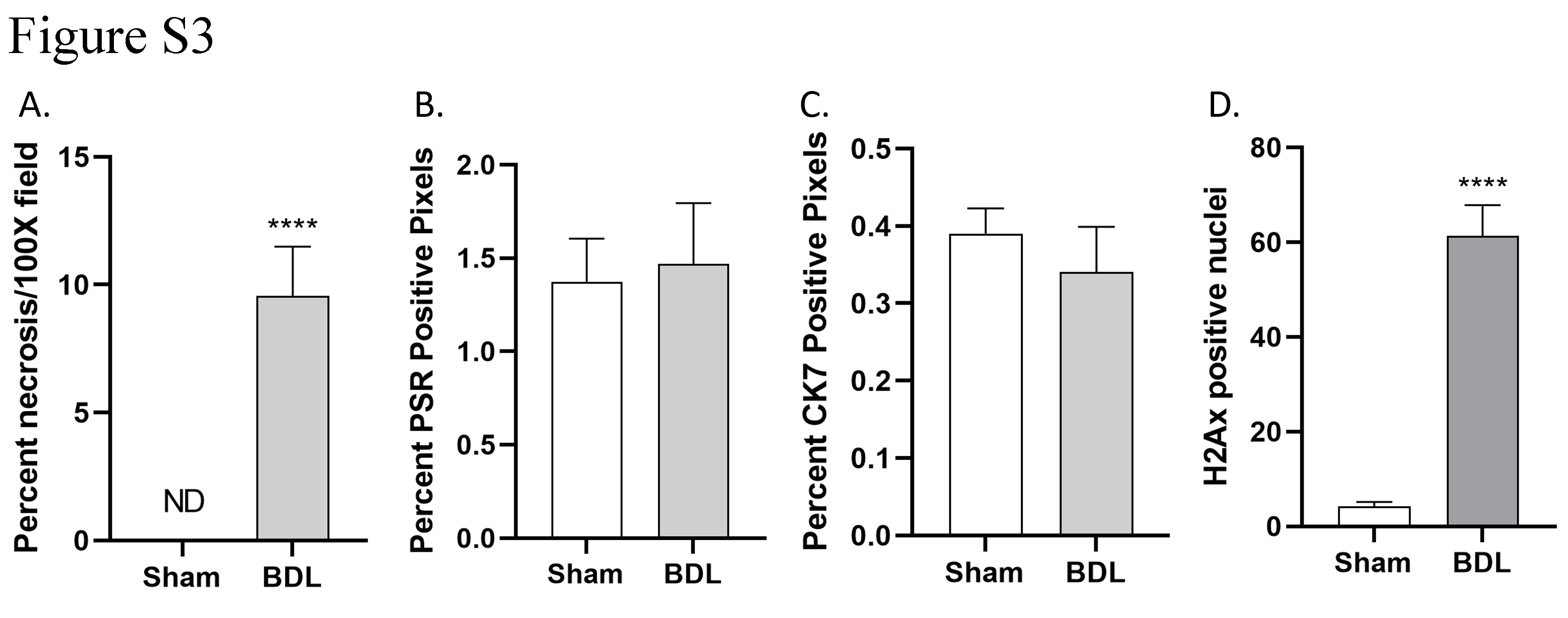

Supplement: S3 Fig — A. Percent necrotic injury. B. Quantification of Picrosirius red staining (PSR). C. Percent cytokeratin 7 (CK7) positive cells. D. qPCR analysis of mRNA for fibrogenic genes Timp1 and Col1a1 in liver tissue from control and 3D BDL mouse liver (N = 4/group). E. H2Ax positive nuclei/100X field. Data were statistically analyzed using Student’s t-test and are presented as Mean ± STDEV. ****p<0.0001, n = 4/group. (TIF) [file pone.0276879.s004.tif]
