# Supplementary material for: The autophagic protein p62 is a target of reactive aldehydes in human and murine cholestatic liver disease
Source: PLoS One. 2022 Nov 15;17(11):e0276879. doi: 10.1371/journal.pone.0276879 (PMC9665405; doi:10.1371/journal.pone.0276879)
Supplement: S1 Raw images — (PDF) [file pone.0276879.s005.pdf]

The autophagic protein p62 is a target of reactive aldehydes in human and murine cholestatic liver disease.

Colin T. Shearn[1], Aimee L. Anderson[1], Michael W. Devereux[1], David J. Orlicky[2], Cole Michel[3], Dennis R. Petersen[3], Colin G. Miller[4], Sanjiv Harpavat[5], E. Edward Schmidt[4,6], and Ronald J. Sokol[1].

Original western blotting images
All lanes were loaded as described in the attached experiments that precede each set of blots below
All blots used the same molecular weight standards (3 microliters per gel)

Exp 186 **4/4/22**

Purpose: Western analysis of LAMP1 and LAMP2 in human PSC

Procedures:

a. Took tissues previously lysed of PSC and Normal.
b. Quantify by BCA and Aliquot samples.

Gel load order for all gels

Gel 1 (LAMP1/GAPDH)

1. MW
2. Space
3. 870 Control
4. 991 Control
5. 1033 Control
6. 1278 Control
7. 1286 Control
8. 1293 Control
9. Space
10. 1128 PSC
11. 1199 PSC
12. 1210 PSC
13. 1307 PSC
14. 1315 PSC
15. 1339 PSC

Gel 2 LAMP2/GAPDH

1. MW
2. Space
3. 870 Control
4. 991 Control
5. 1033 Control
6. 1278 Control
7. 1286 Control
8. 1293 Control
9. Space
10. 1128 PSC
11. 1199 PSC
12. 1210 PSC
13. 1307 PSC
14. 1315 PSC
15. 1339 PSC

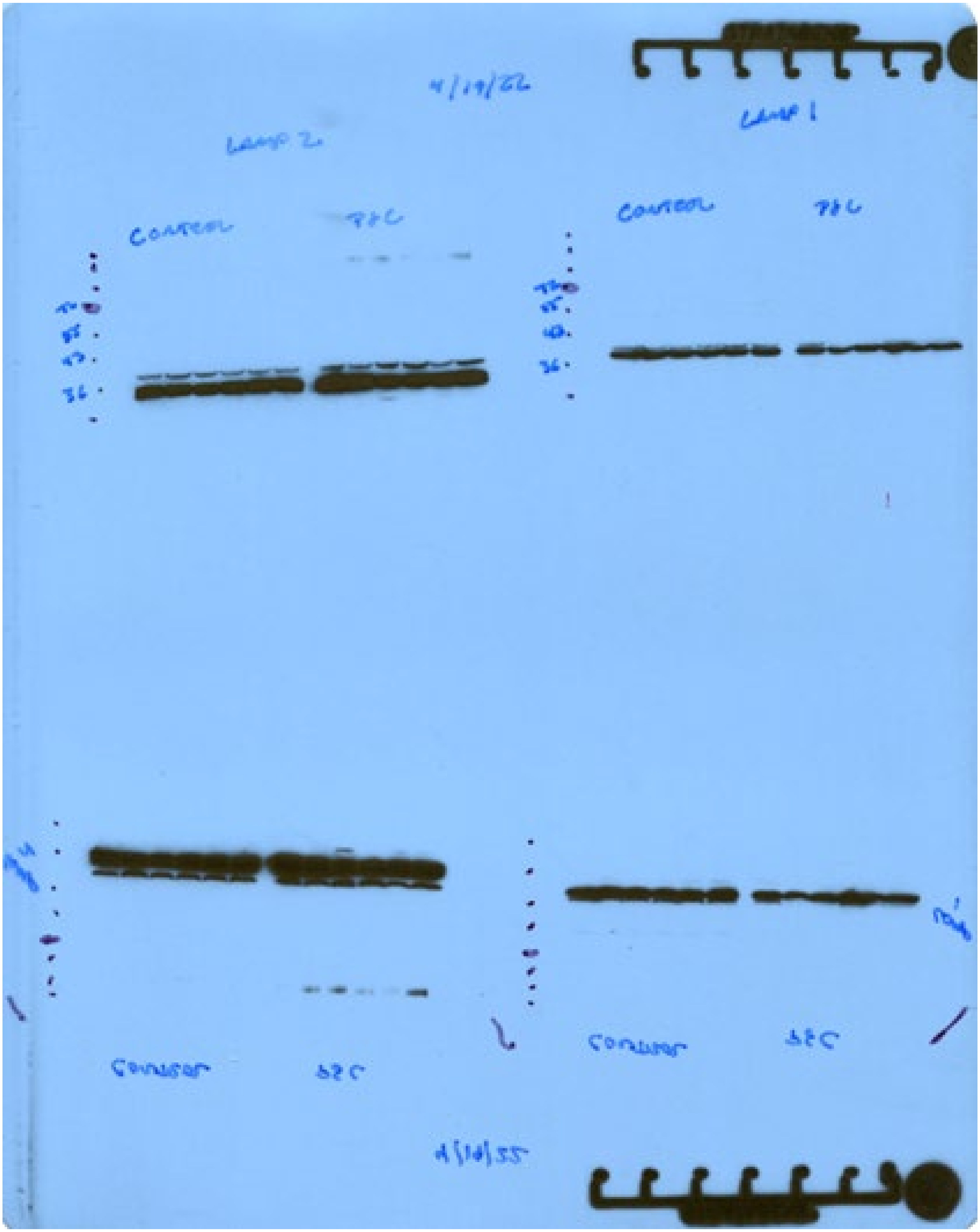
4/19/22
Lamp 2
Control
37C
Lamp 1
Control
37C
36

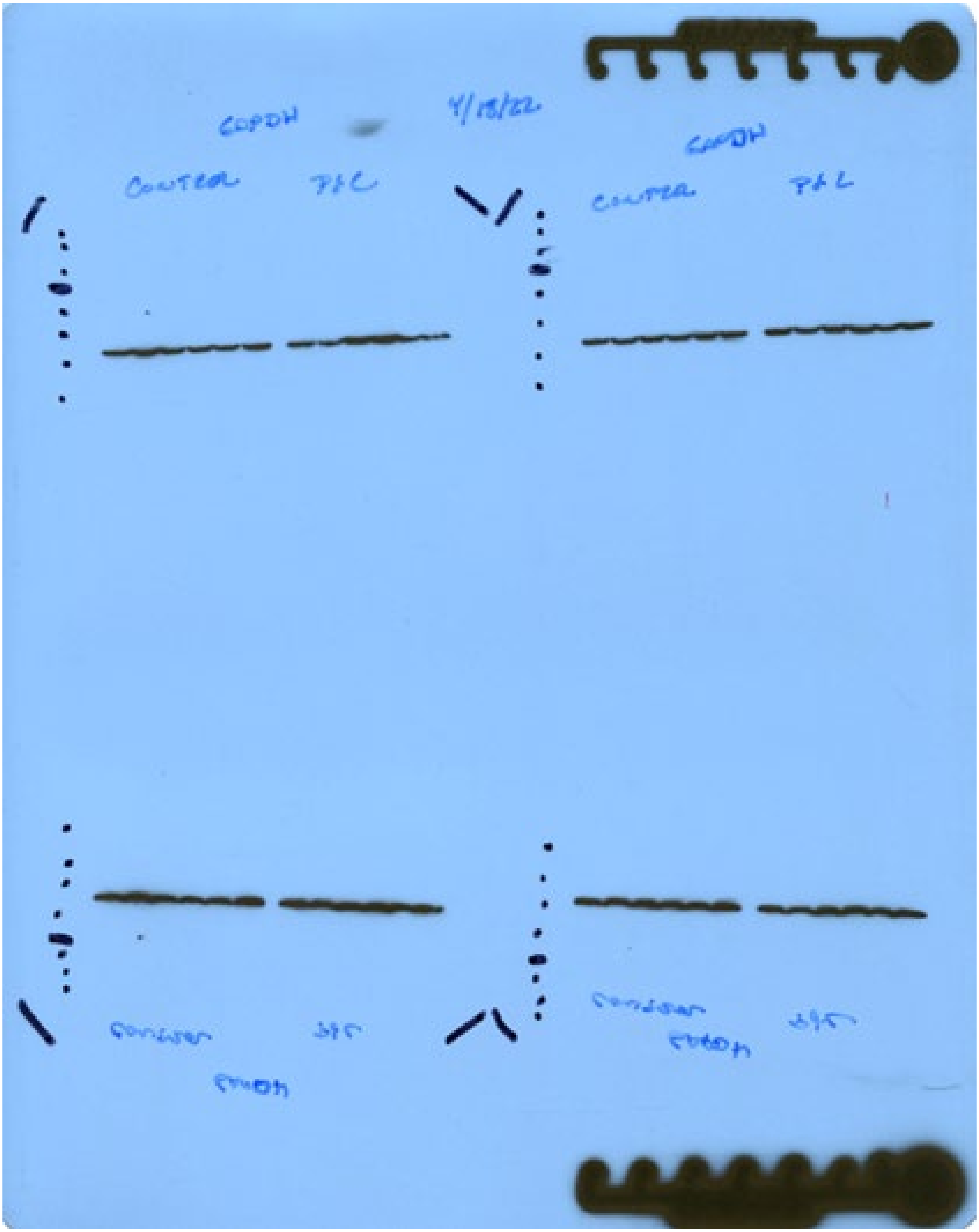

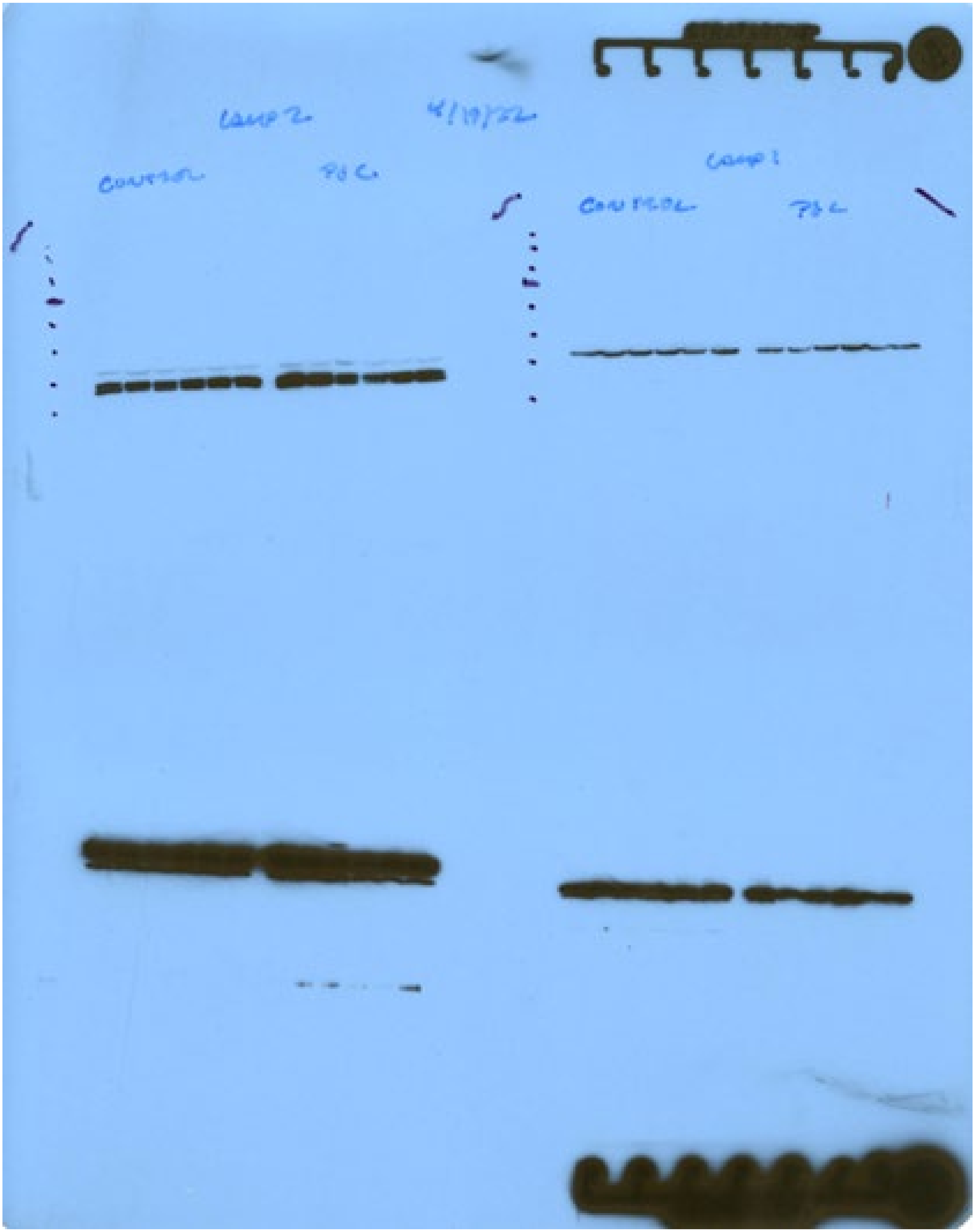
4/19/22
CONTROL
73C
CONTROL
73C

Exp 128 **10-08-21**

Purpose: Preparation of whole cell extracts normal and PSC tissue for Western analysis of autophagy

Procedures:

c. Took tissues previously lysed of PSC and Normal.
d. Quantify by BCA and Aliquot samples.

Exp 128 Normal and PSC WCE			CO-IP		WCE for WB			
	genotype	mg/ml	500μg		1000μg	PBS	5X SDS	tot vol
870	Normal	33.73	14.82		29.64	370.36	100	500.00
991	Normal	27.20	18.38		36.76	363.24	100	500.00
1033	Normal	24.00	20.83		41.67	358.33	100	500.00
1044	Normal	10.07	49.67		99.34	300.66	100	500.00
1278	Normal	22.53	22.19		44.38	355.62	100	500.00
1286	Normal	28.33	17.65		35.29	364.71	100	500.00
1293	Normal	15.80	31.65		63.29	336.71	100	500.00
1199	PSC	8.27	60.48		120.97	279.03	100	500.00
1270	PSC	8.87	56.39		112.78	287.22	100	500.00
1307	PSC	9.00	55.56		111.11	288.89	100	500.00
1315	PSC	7.67	65.22		130.43	269.57	100	500.00
1339	PSC	8.60	58.14		116.28	283.72	100	500.00
1358	PSC	9.73	51.37		102.74	297.26	100	500.00

1. Prepare samples and examine expression of p62 (Sqstm1), ATG5, ATG7, ATG12, LC3, Beclin
1.

Gel load order for all gels

1. MW
2. Space
3. 870 Control
4. 991 Control
5. 1033 Control
6. 1278 Control
7. 1286 Control
8. 1293 Control
9. Space
10. 1128 PSC
11. 1199 PSC
12. 1210 PSC
13. 1307 PSC
14. 1315 PSC
15. 1339 PSC

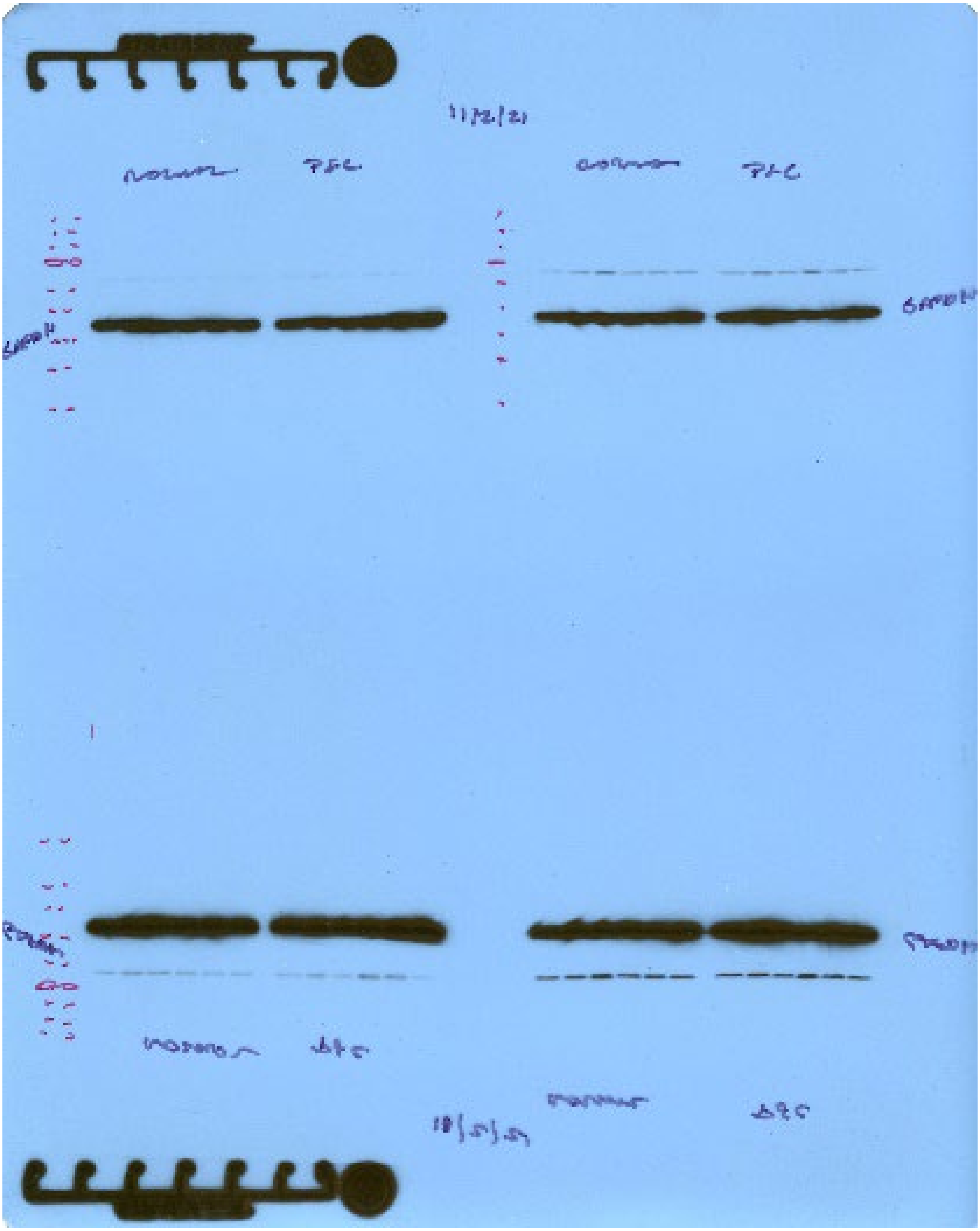

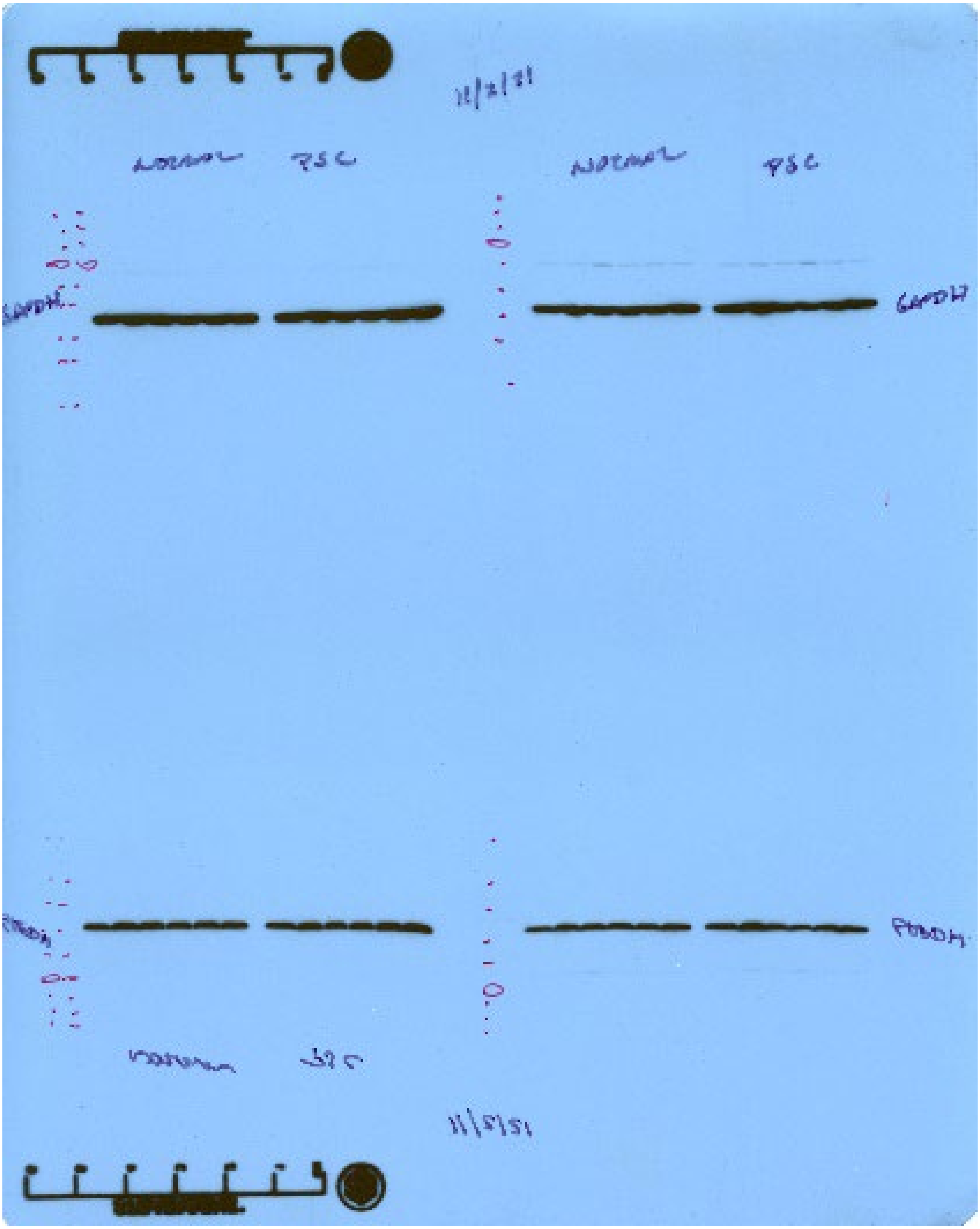
NORMAL
PSC
NORMAL
PSC
GAPDH
GAPDH

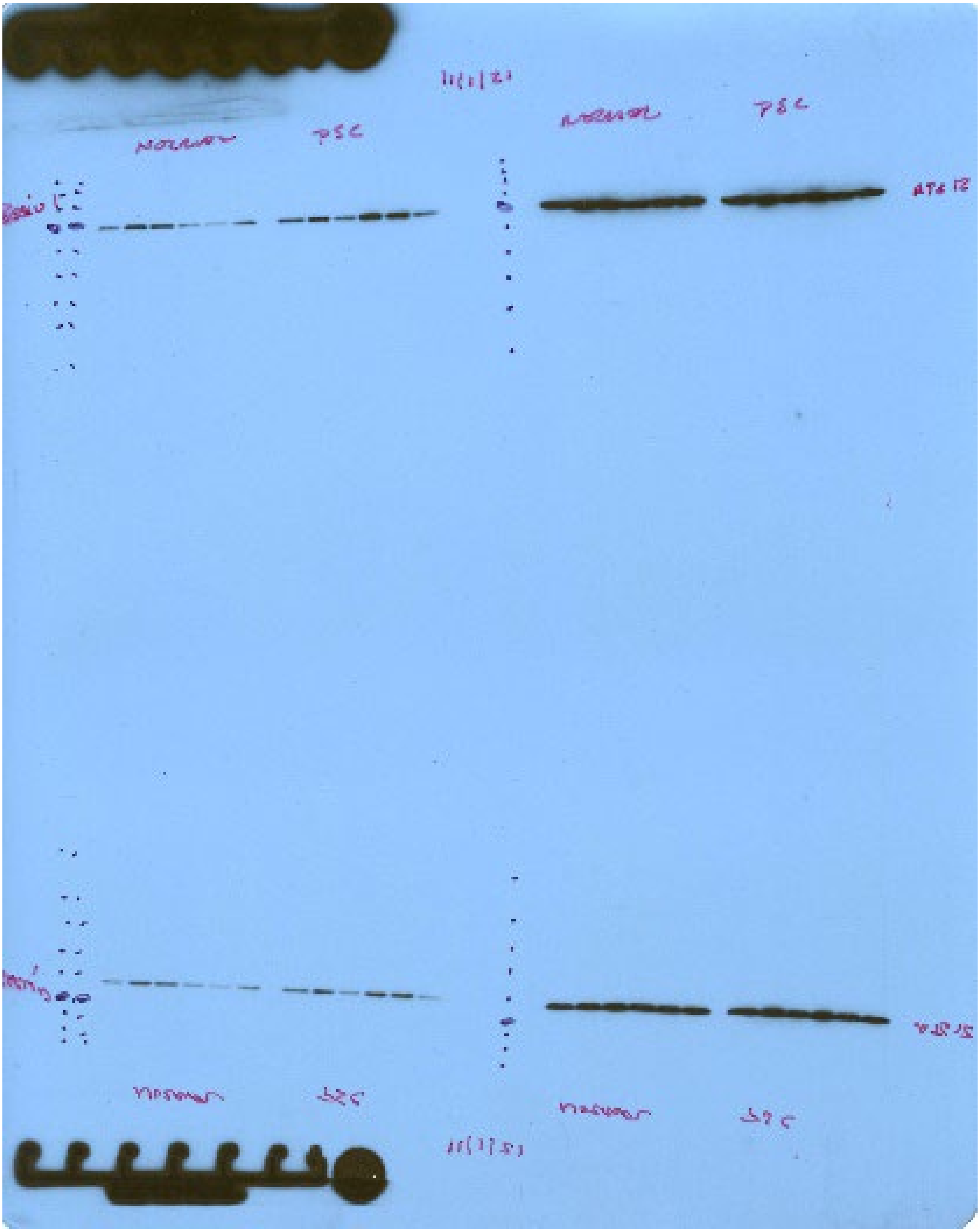
NORMAL
PSC
NORMAL
PSC
ATG 12

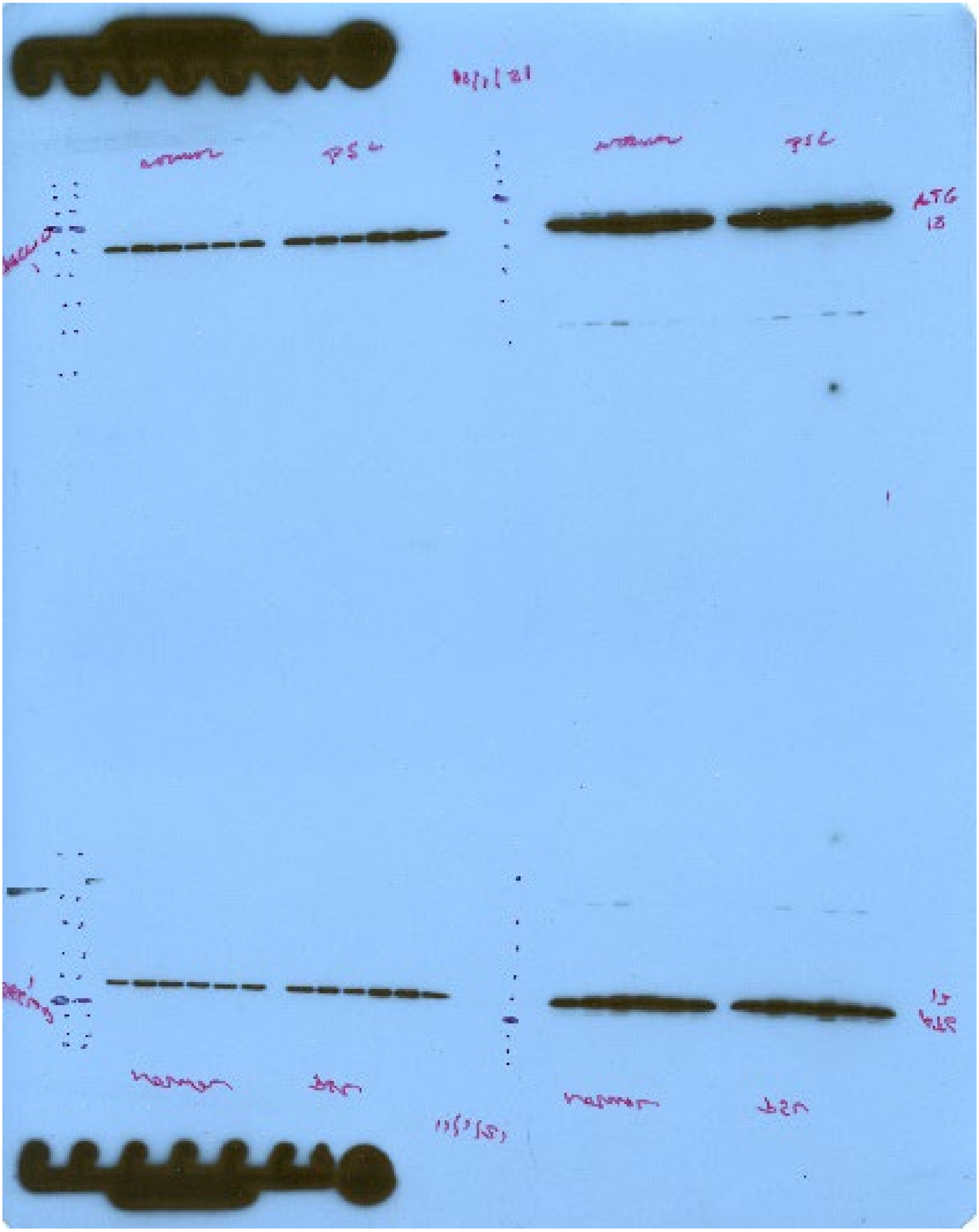
PSC
PSC
ATG
13

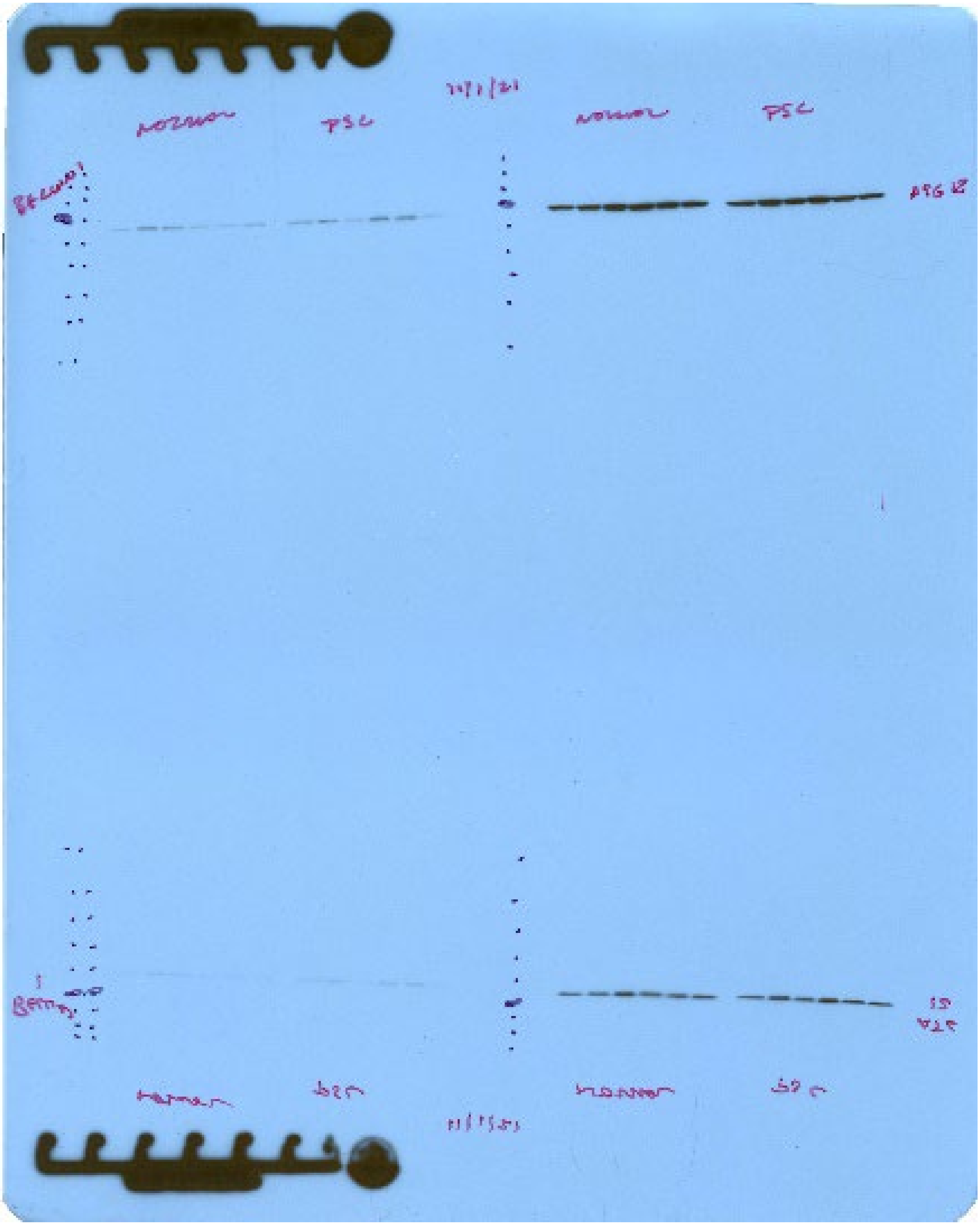
PSC
PSC

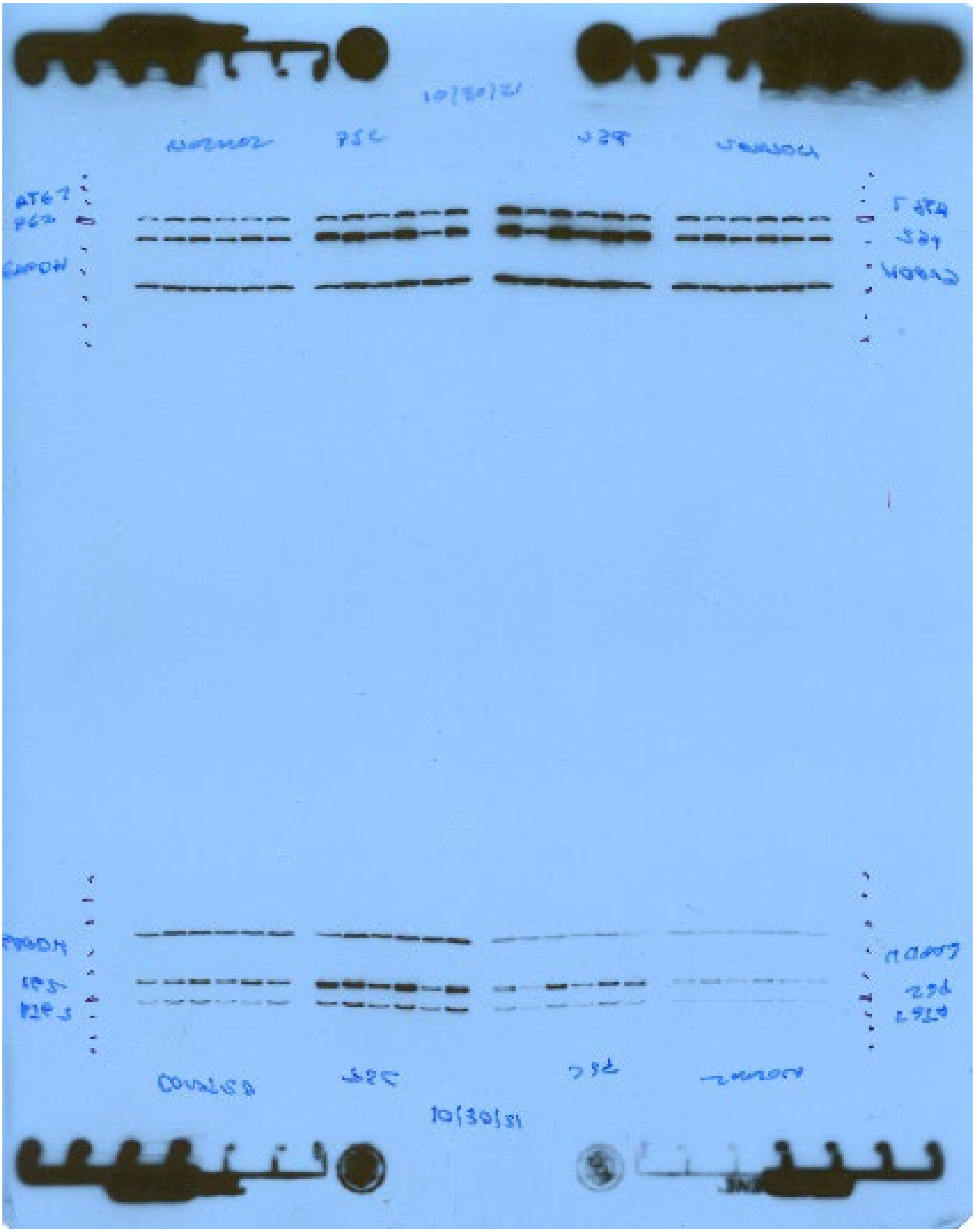
10/30/21
PSC
ATG7
P62
GAPDH
10/30/21

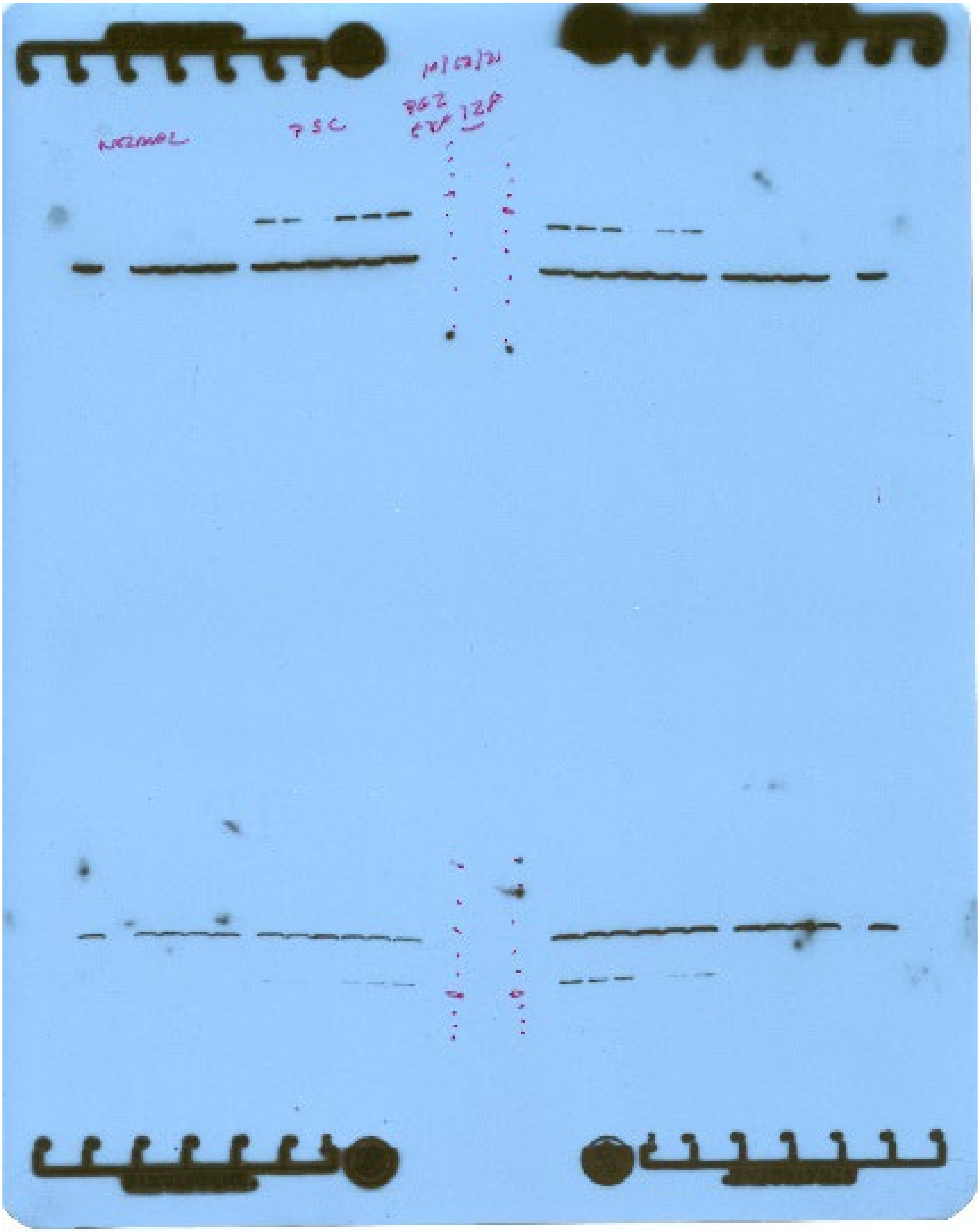
11/02/21
NORMAL
PSC

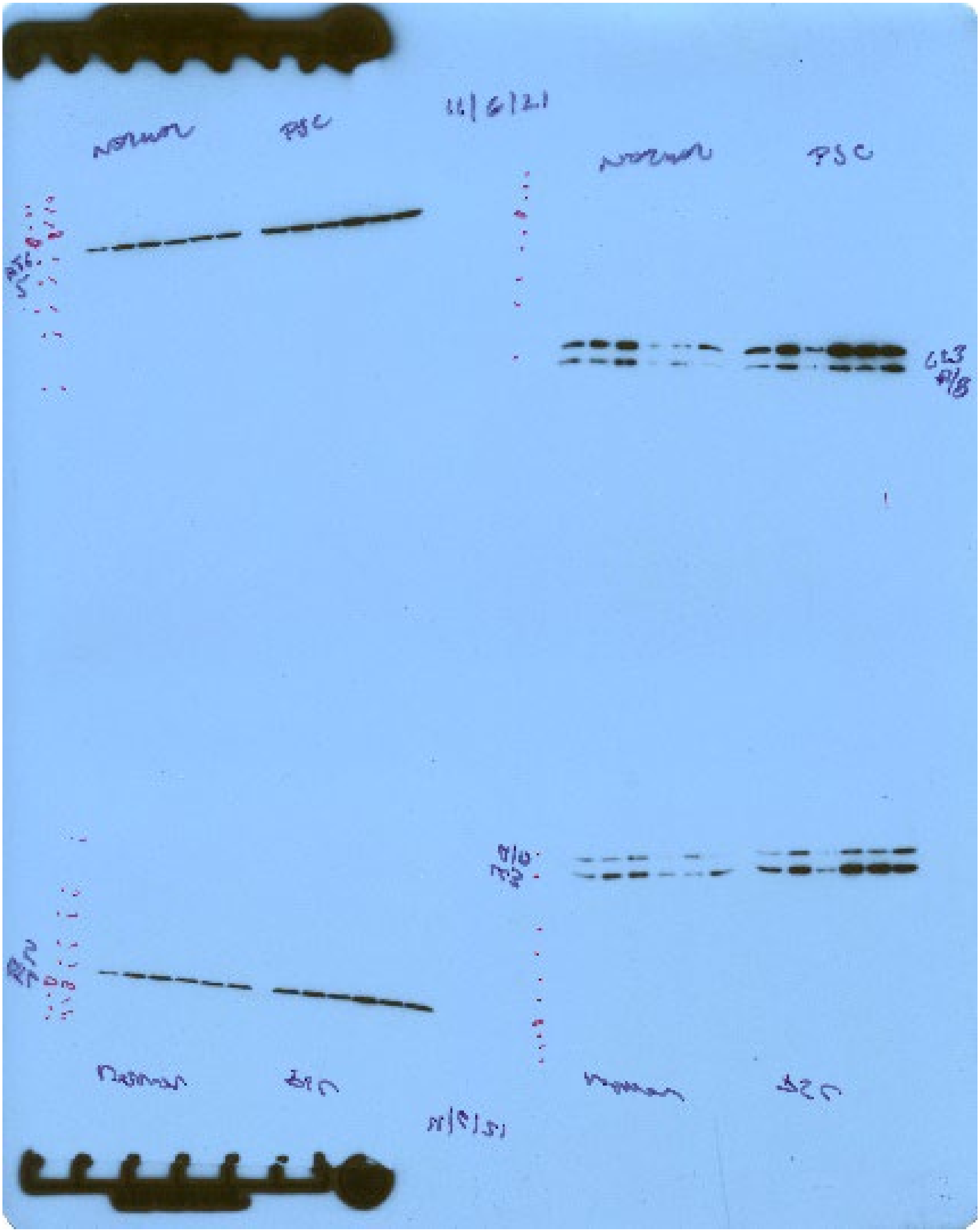
11/6/21
Normal
PSC
Normal
PSC

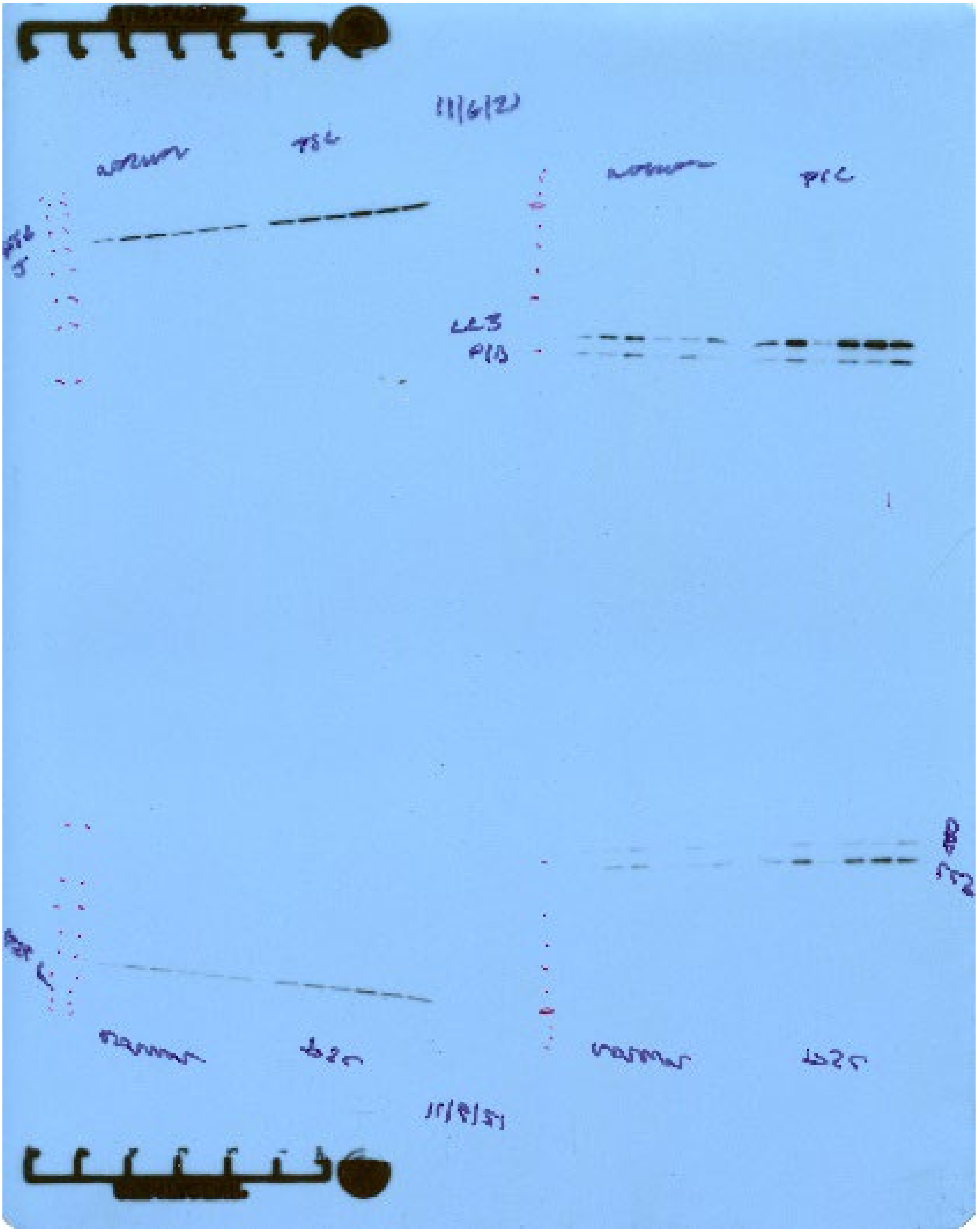
11/6/21
normal
TSC

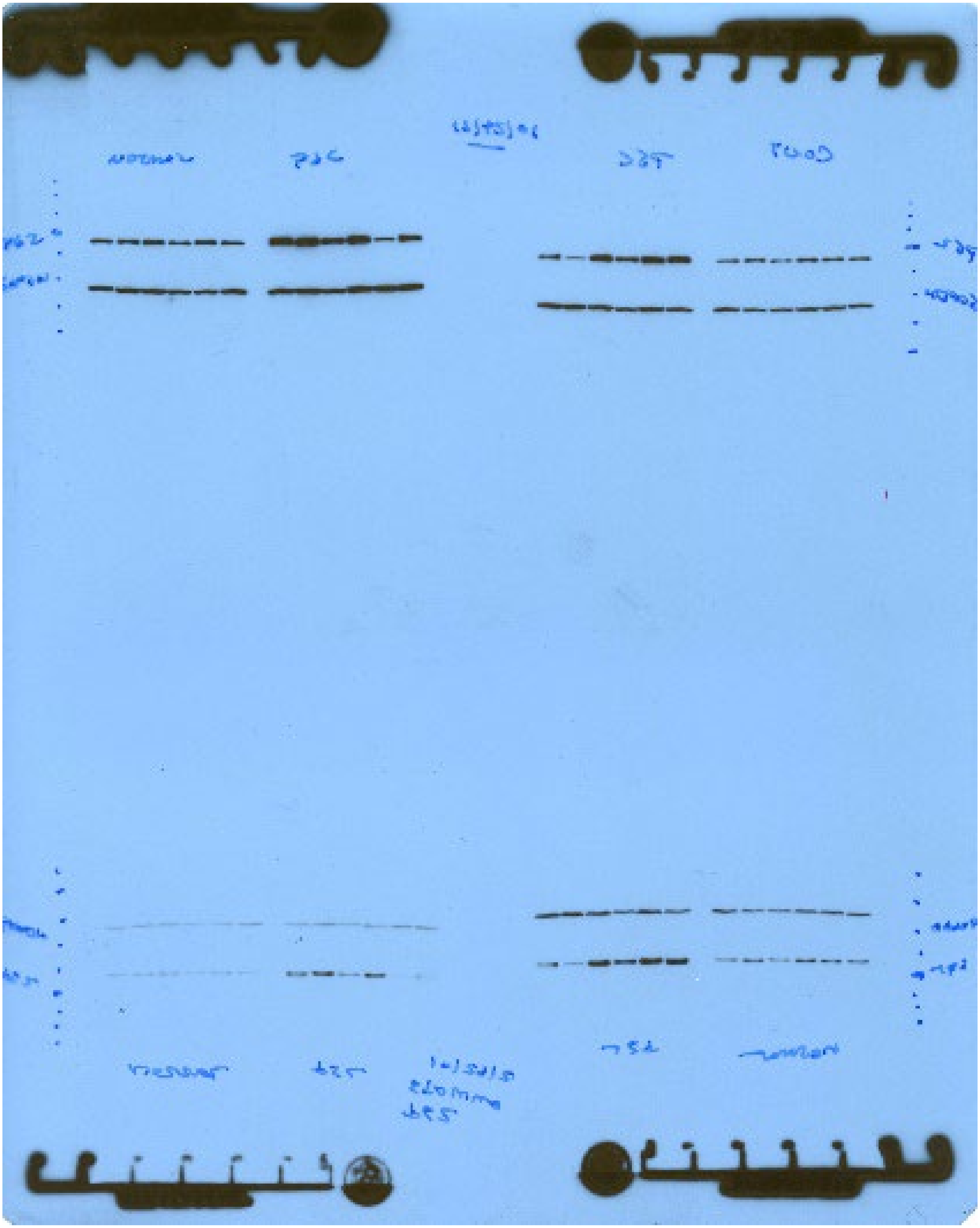

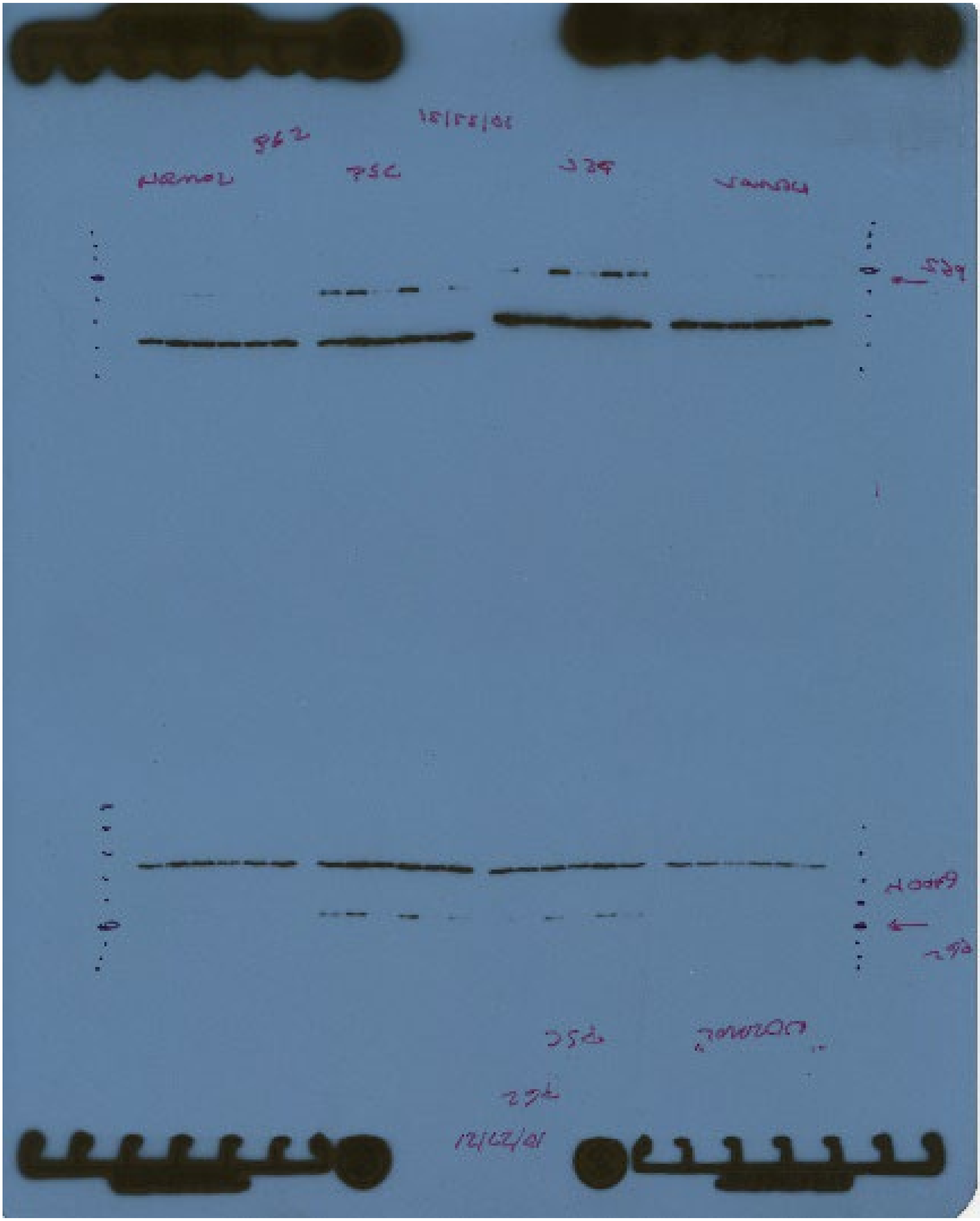

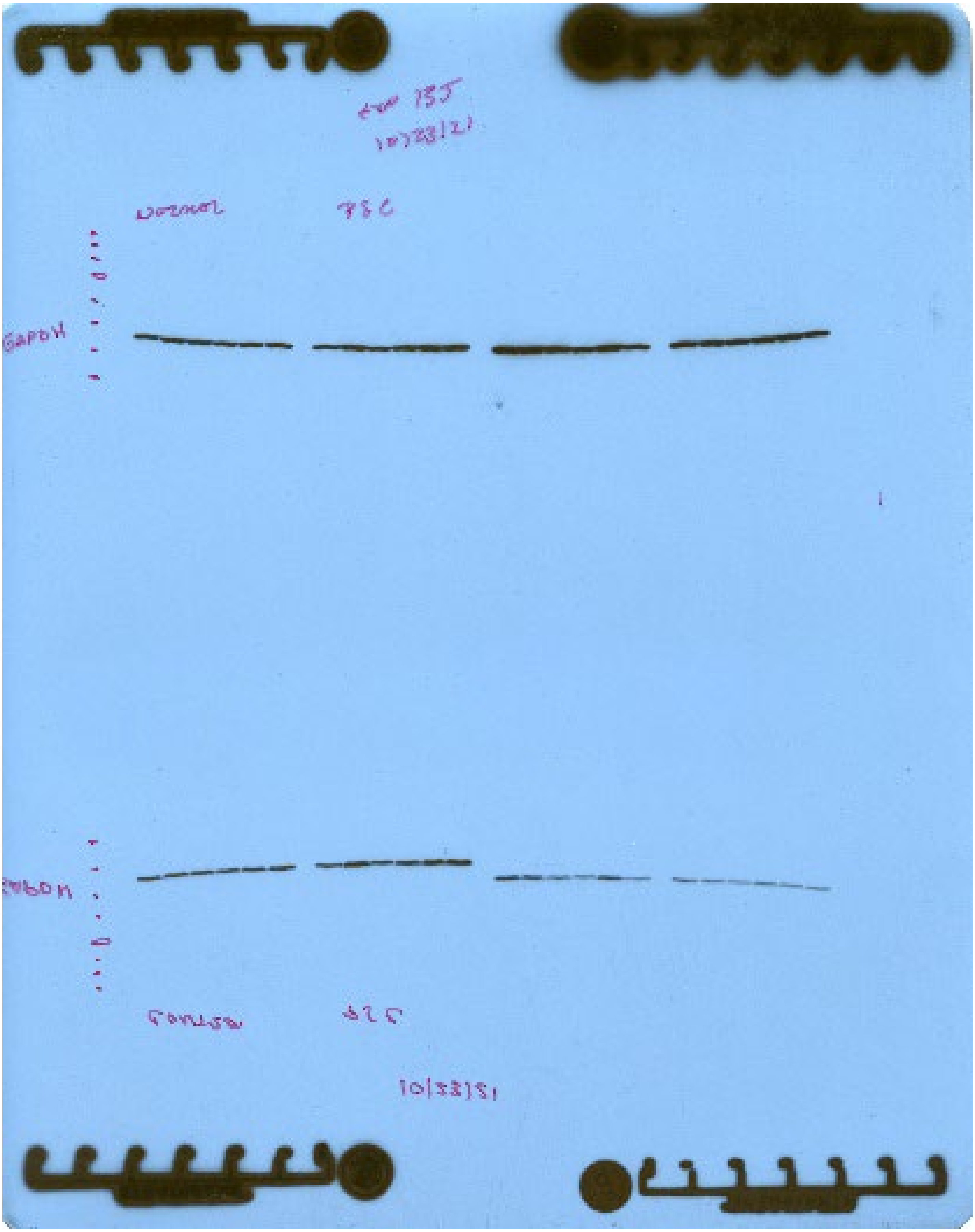
GAPDH

CS-093 **06-07-21**

Purpose: Examining the expression of autophagy markers in Sham and BDL mice. These data will round out the data for the autophagy study.

Procedures:

a. Resuspended tissue in approximately 400λ of 1mM NaCl + 1/100 mammalian protease inhibitor cocktail (P8340 Sigma) for 15 minutes.
b. Sonicated tissue for 3X15 seconds using handheld sonicator.
c. Add 100λ of 5X cell lysis buffer (500mM NaCl, 5mM EDTA, 500mM Tris pH 8.0 plus 0.5mM Triton X-100 plus protease inhibitors.
d. Sonicate an additional 1X15 seconds.
e. Spin samples for 5 minutes at 14000 RPM 4ºC.
f. Quantify by BCA, Boil in 1X SDS loading buffer and Aliquot samples.

Gel load order:

1. MW
2. space
3. mouse number 147 Sham 3 Day
4. mouse number 148 Sham 3 Day
5. mouse number 149 Sham 3 Day
6. mouse number 150 Sham 3 Day
7. mouse number 151 Sham 3 Day
8. space
9. mouse number 1 BDL 3 Day
10. mouse number 2 BDL 3 Day
11. mouse number 13 BDL 3 Day
12. mouse number 14 BDL 3 Day
13. mouse number 16 BDL 3 Day
14.

1. Western blot for expression of p62 (Sqstm1), ATG5/12, ATG7, LC3b, Beclin 1 (Becn1)-LAMP1 LAMP2

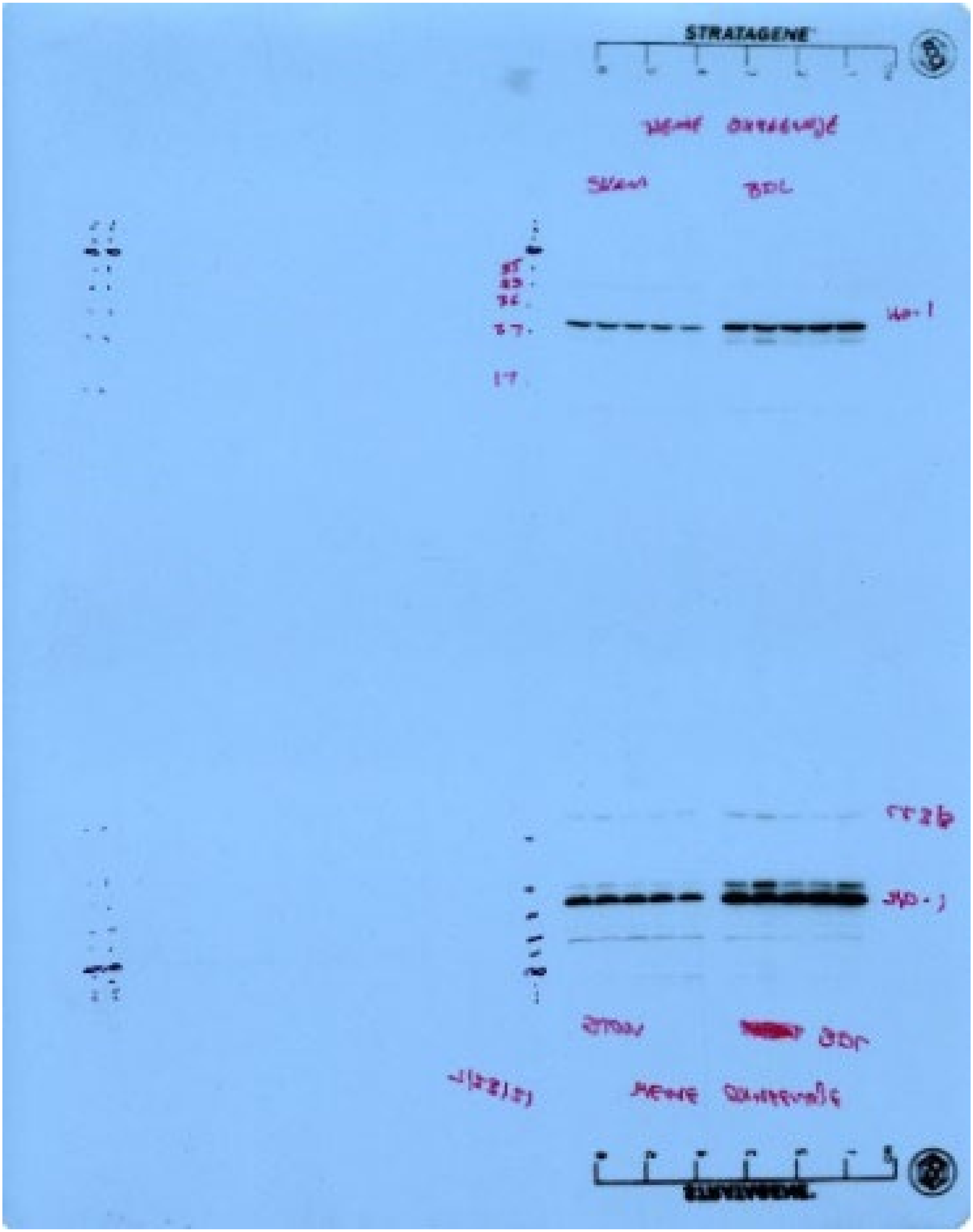
STRATAGENE

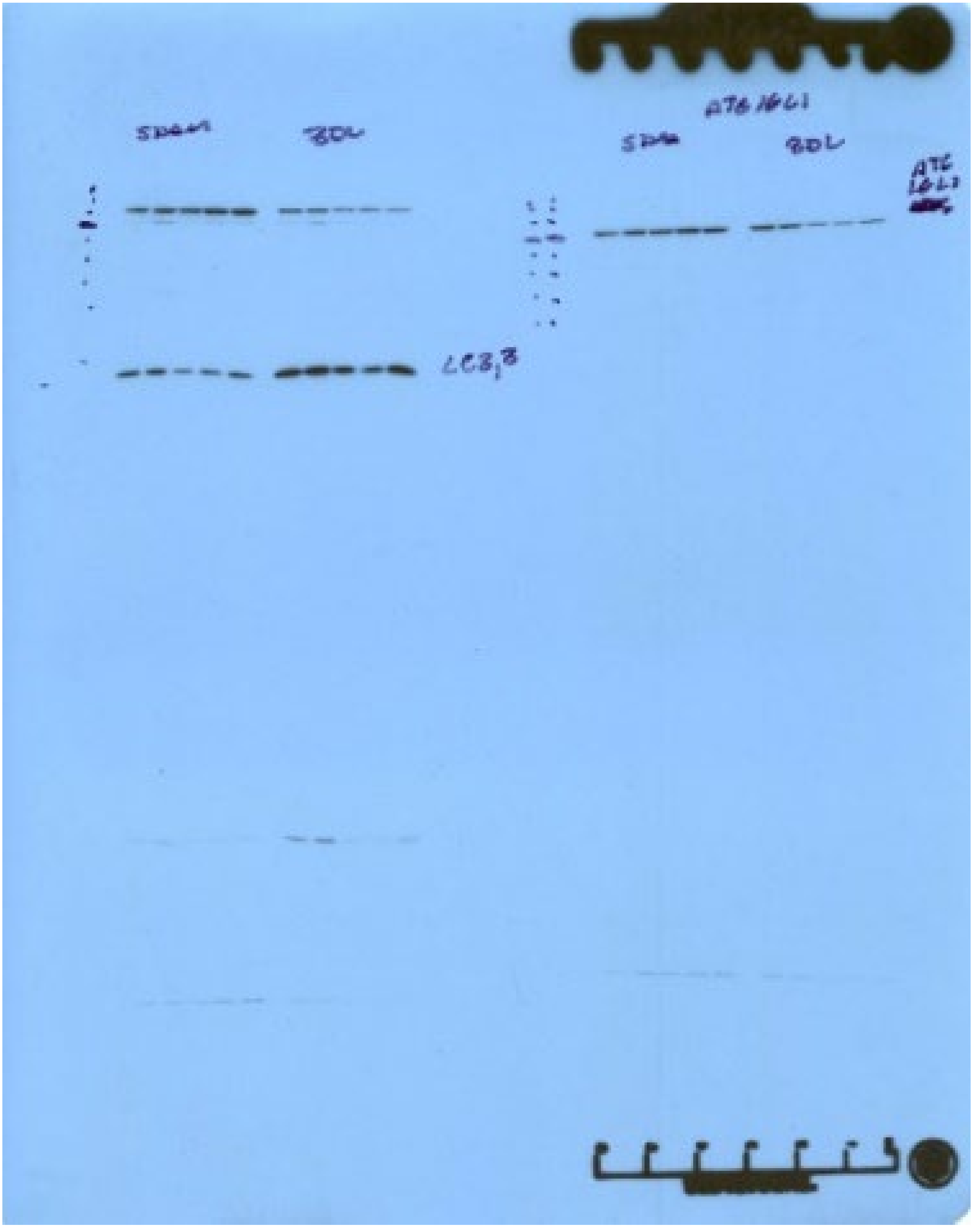

SHAM
BDL
ATG16L1
BDL
LC3B

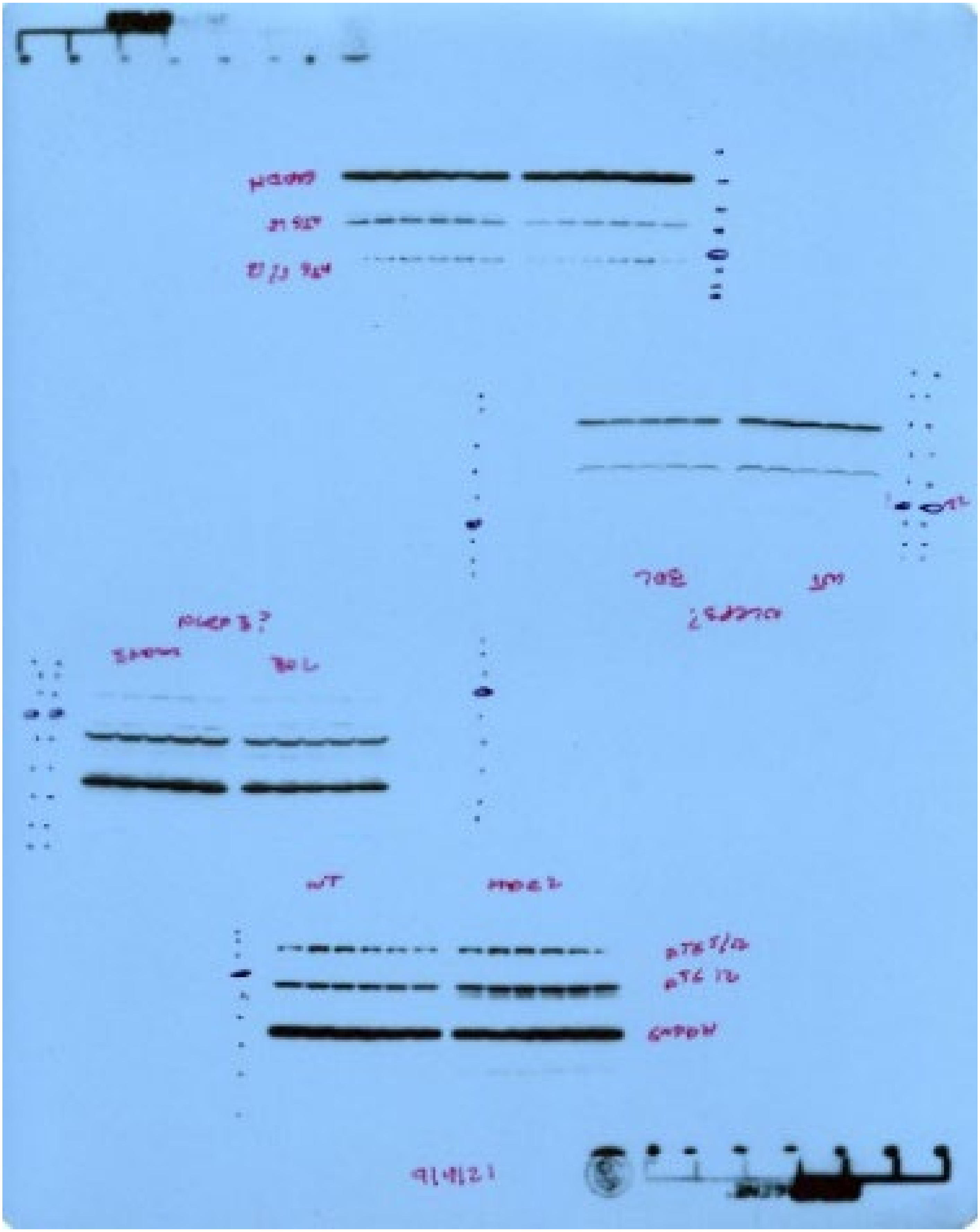
WT
GAPDH

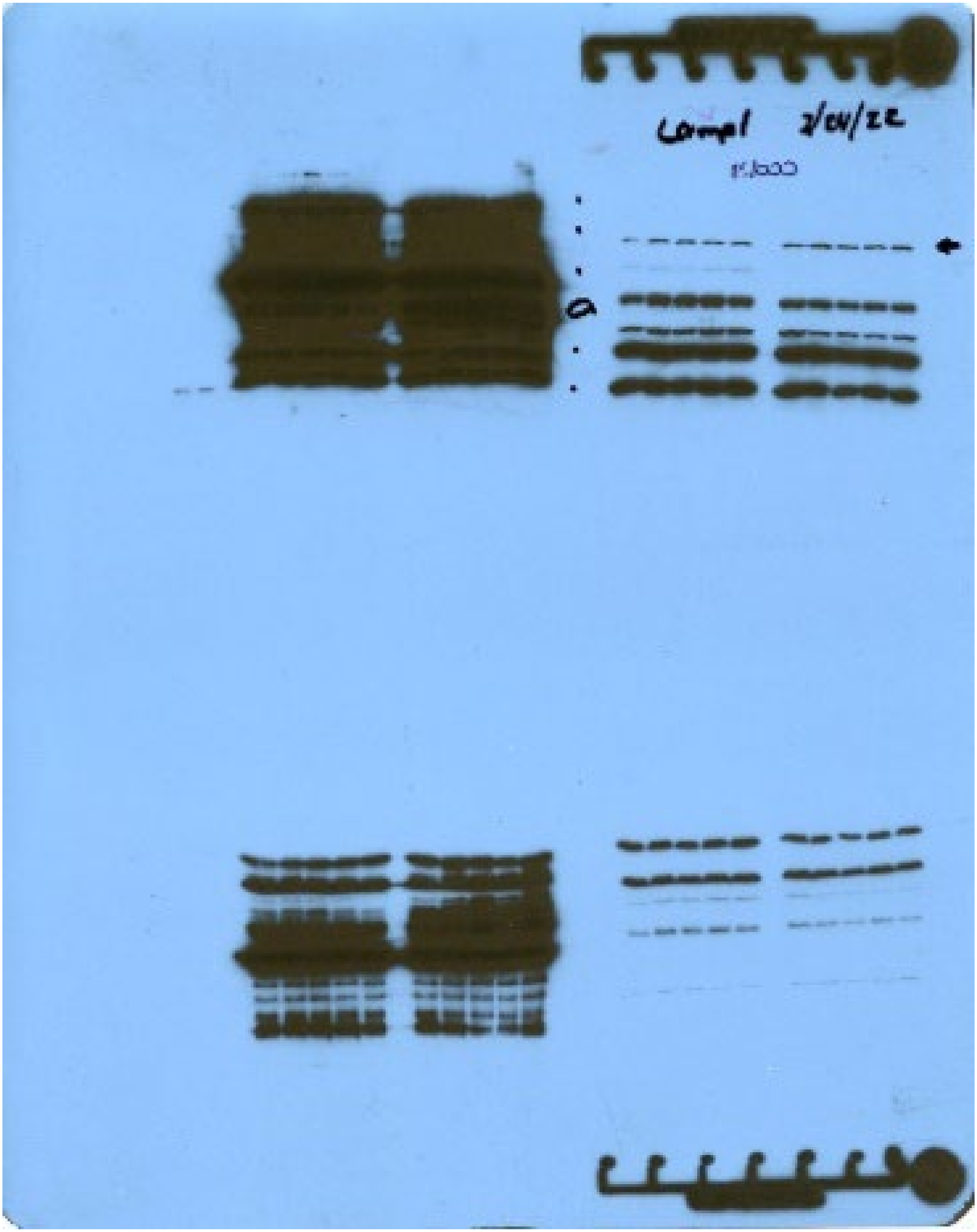
Lamp1
2/04/22

Exp 222 **07-22-22**

Purpose: Preparation of whole cell extracts from WT and MDR2KO for western blotting autophagy paper

Procedures:

a. Resuspended tissue in approximately 400λ of 1mM NaCl + 1/100 mammalian protease inhibitor cocktail (P8340 Sigma) for 15 minutes.
b. Sonicated tissue for 3X15 seconds using handheld sonicator.
c. Add 100λ of 5X cell lysis buffer (500mM NaCl, 5mM EDTA, 500mM Tris pH 8.0 plus 0.5mM Triton X-100.
d. Sonicate an additional 1X15 seconds
e. Spin samples for 5 minutes at 14000 RPM 4ºC.
f. Quantify by BCA, Boil in SDS loading buffer and Aliquot samples.
g. Western blot for expression of p62 (Sqstm1), ATG5/12, ATG7, LC3b, Beclin 1 (Becn1)-LAMP1 LAMP2

Gel load order
1 MW
2 Space
3 83 WT
4 84 WT
5 85 WT
6 86 WT
7 87 WT
8 88 WT
9 space
10 48 MDR2
11 61 MDR2
12 62 MDR2
13 63 MDR2
14 64 MDR2
15 65 MDR2

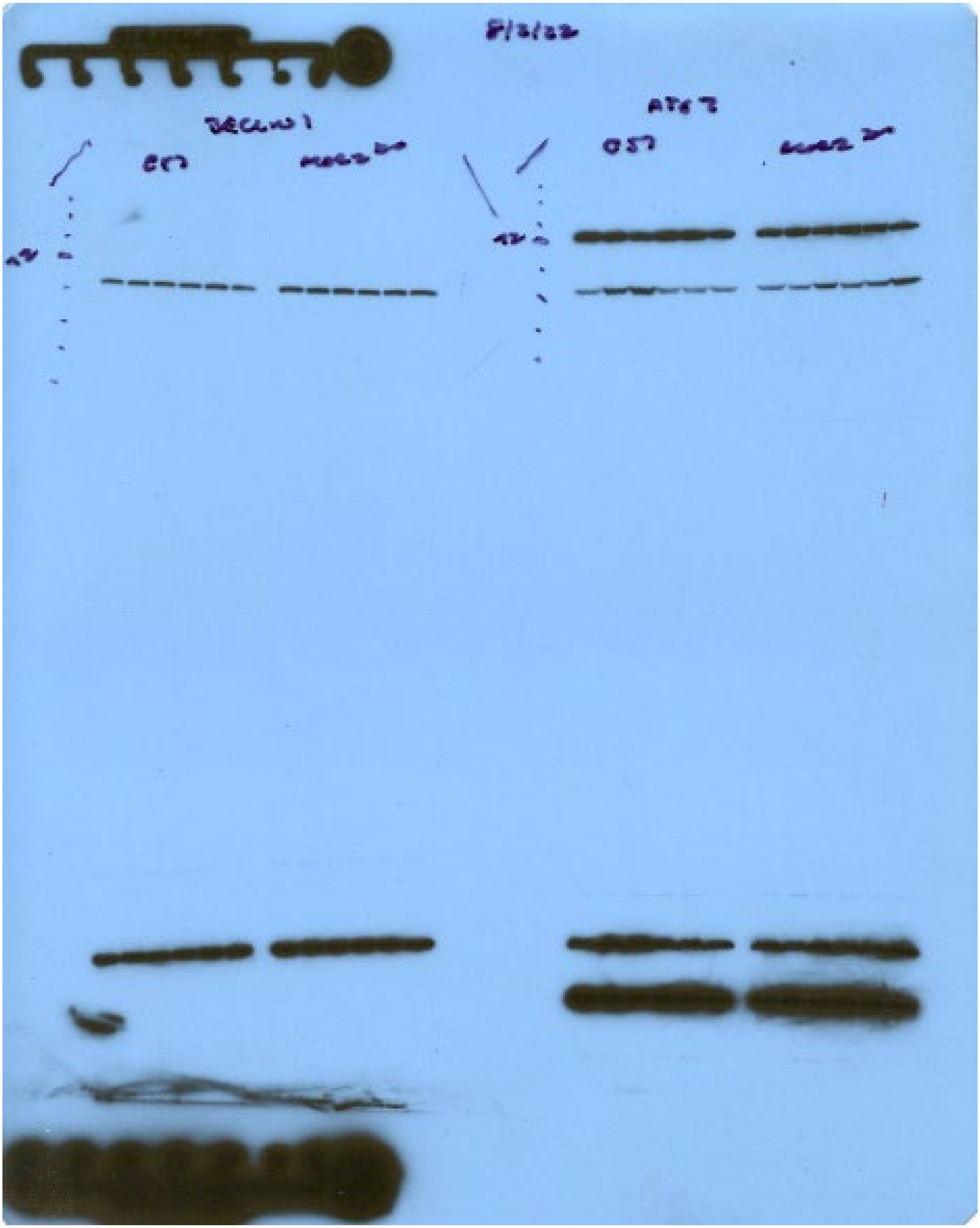
8/3/22
42
42

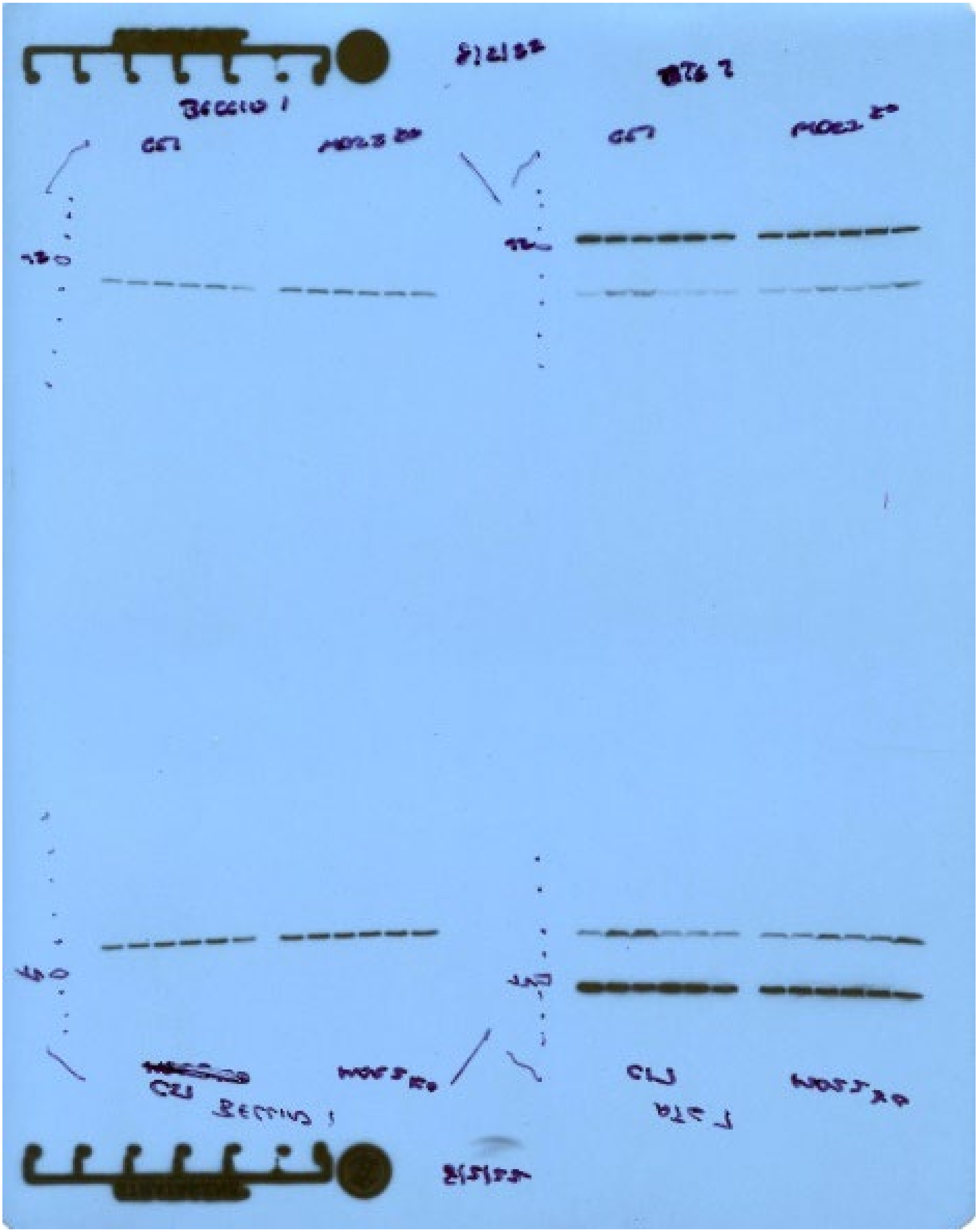

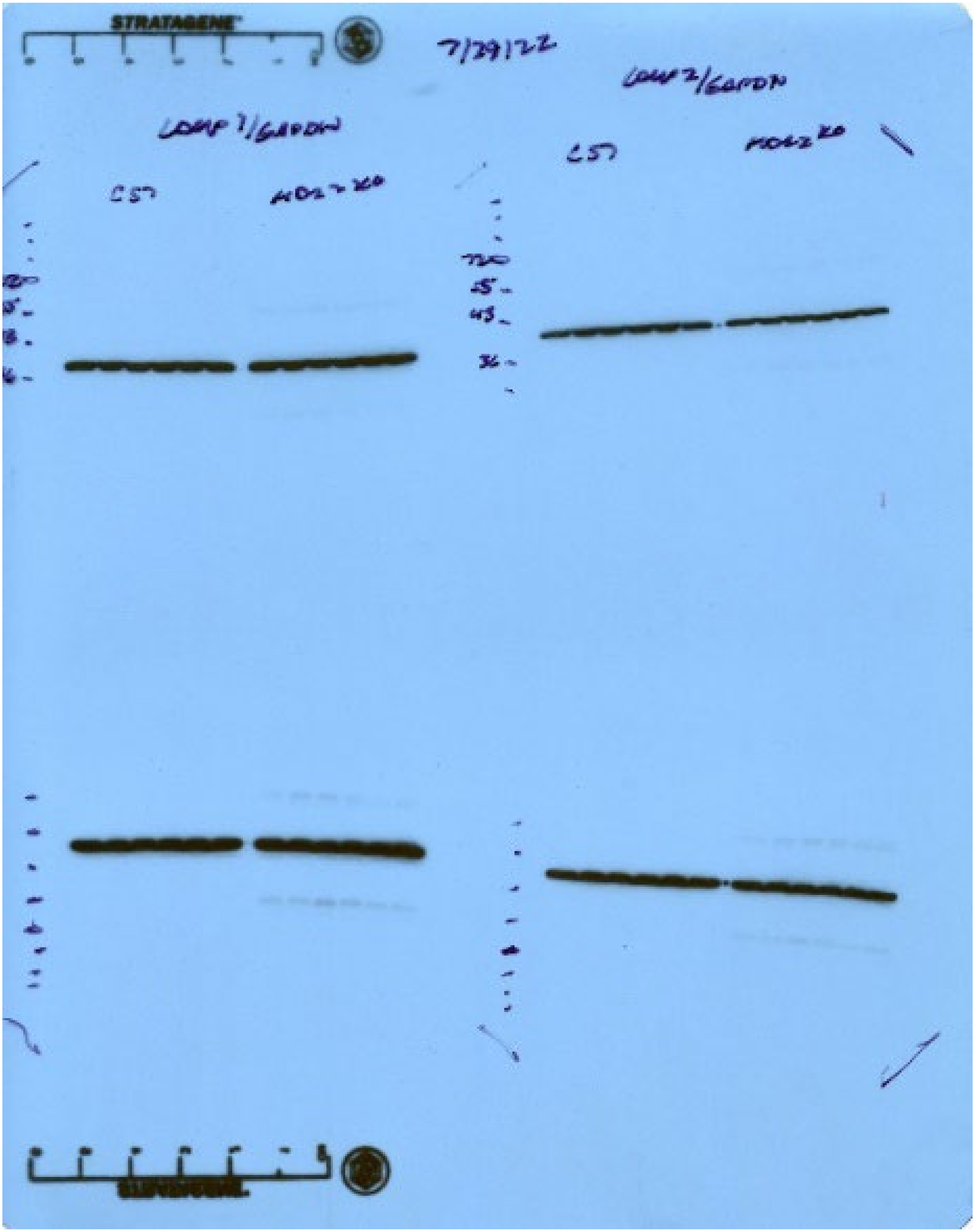
7/29/22
C57
C57
45
43
36

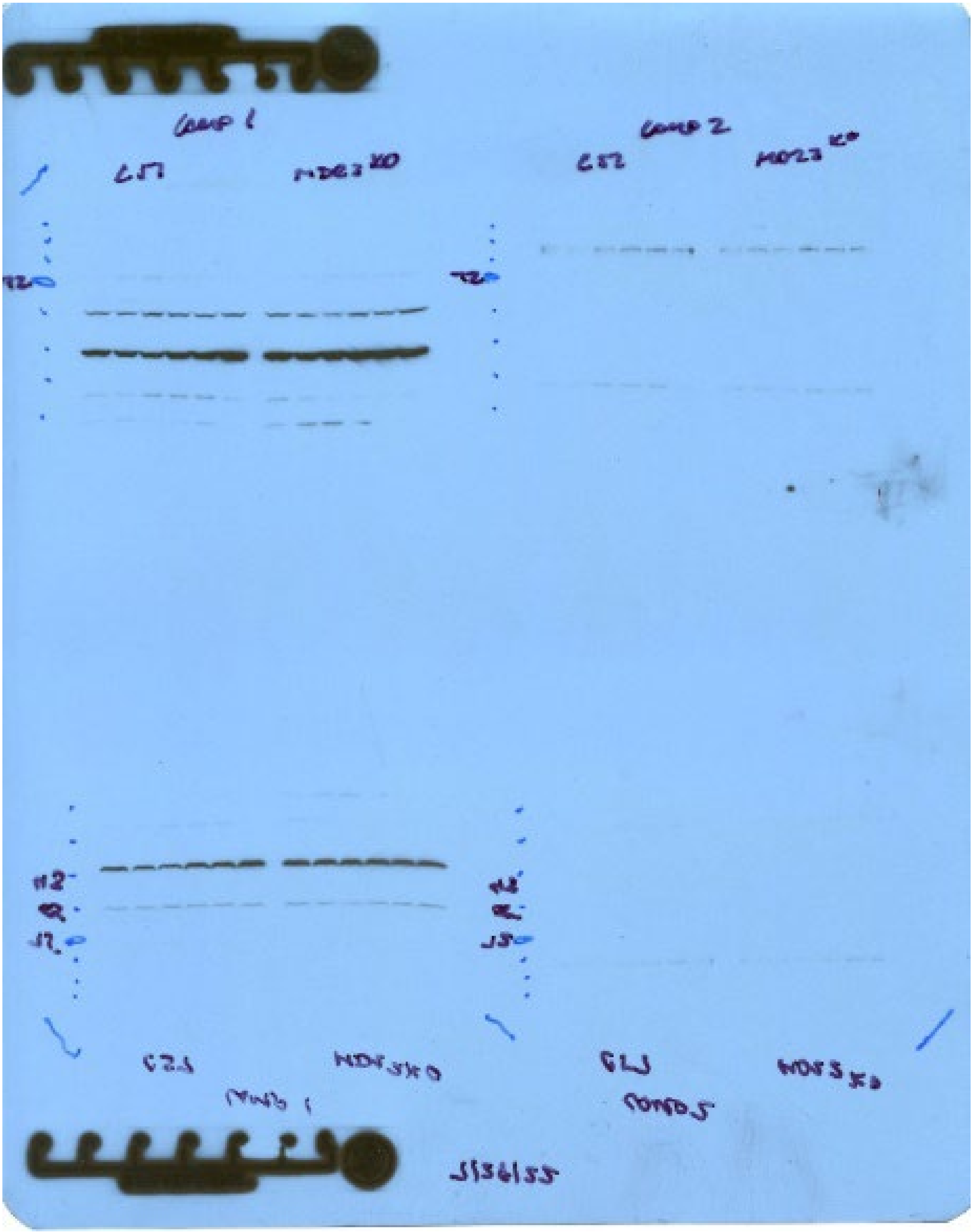

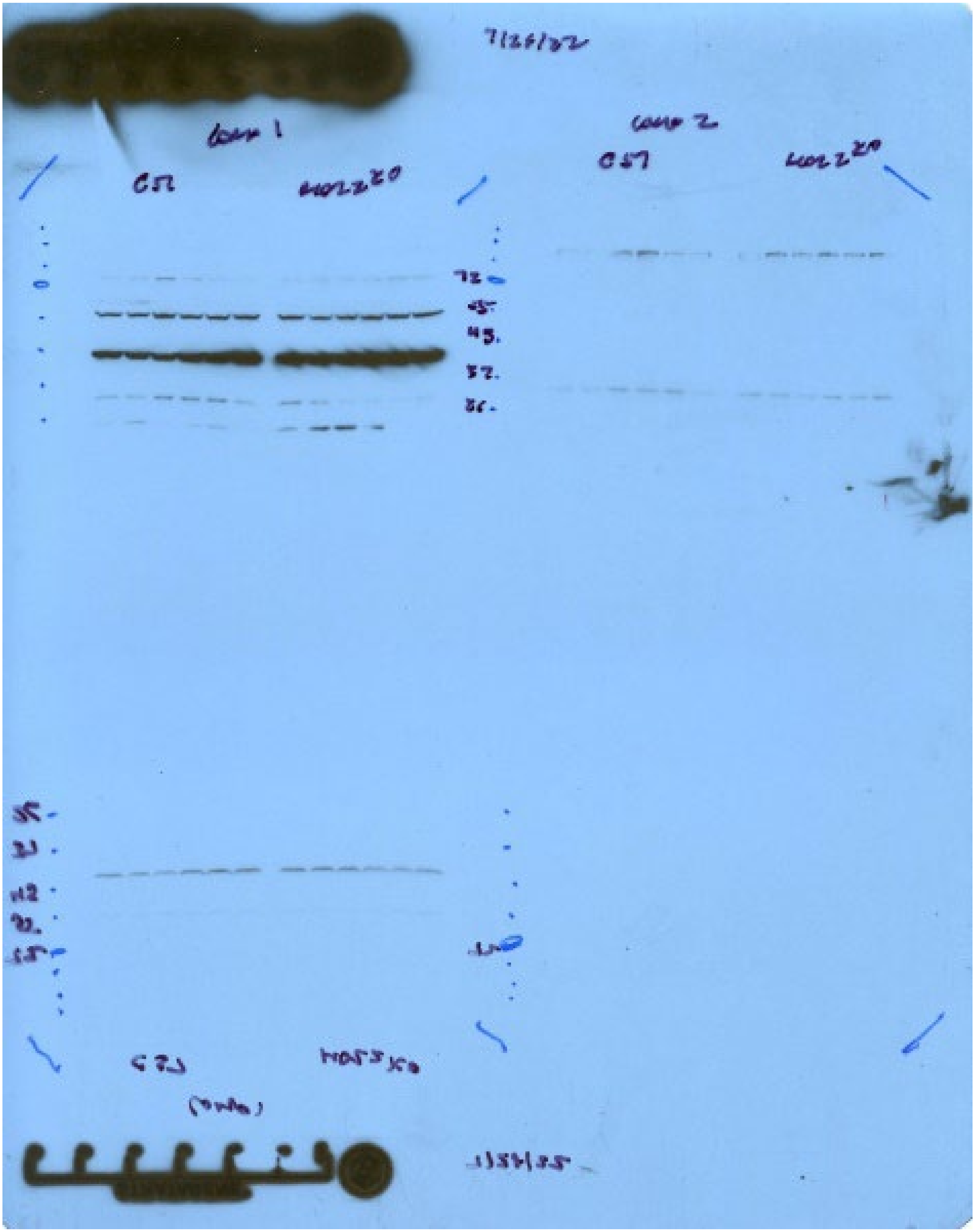

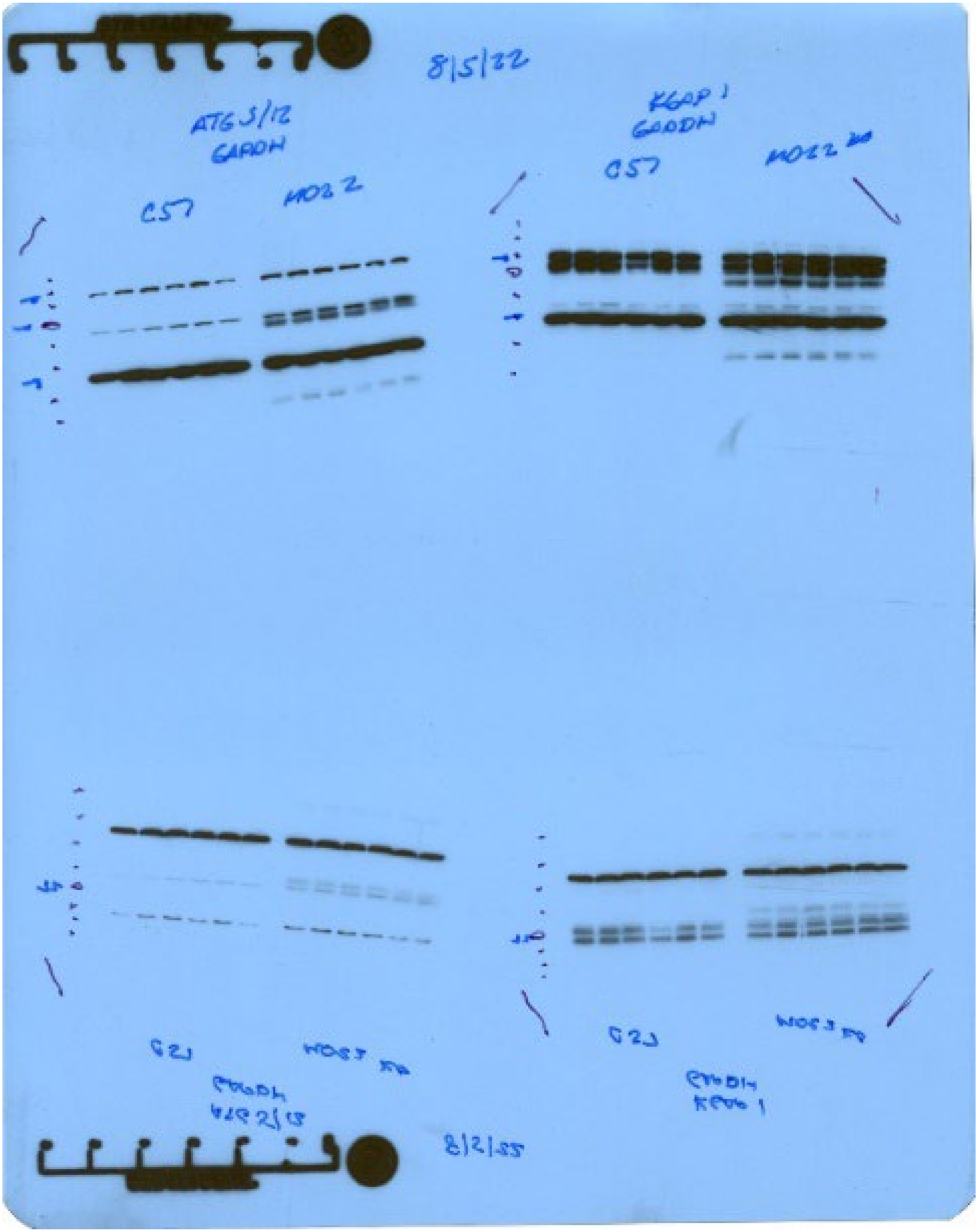

8/5/22
ATG 5/12
GAPDH
C57
MO22
KEAP 1
GAPDH
C57
MO22

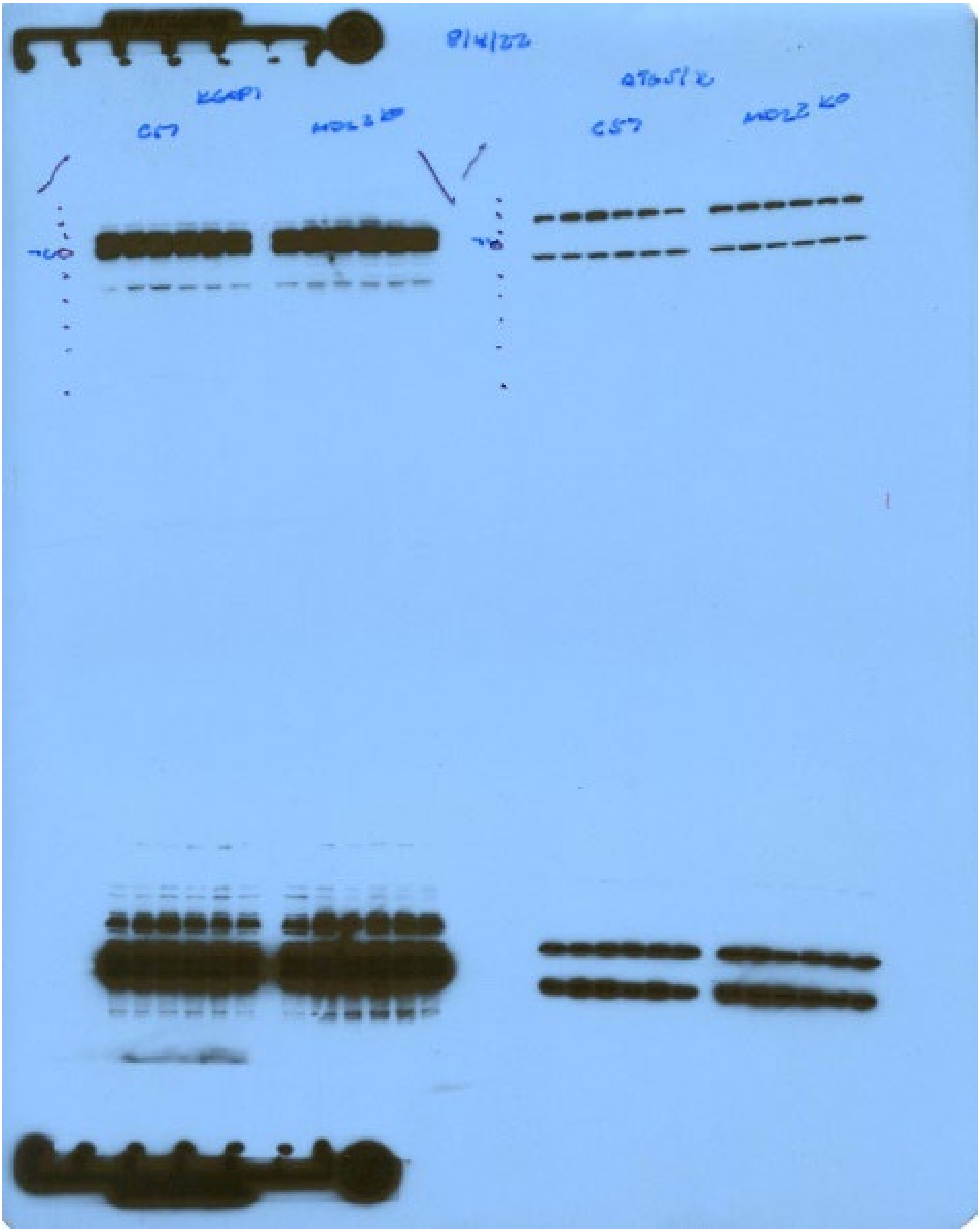
8/4/22
ATG5/12
C57
NOX2 KO
C57
NOX2 KO

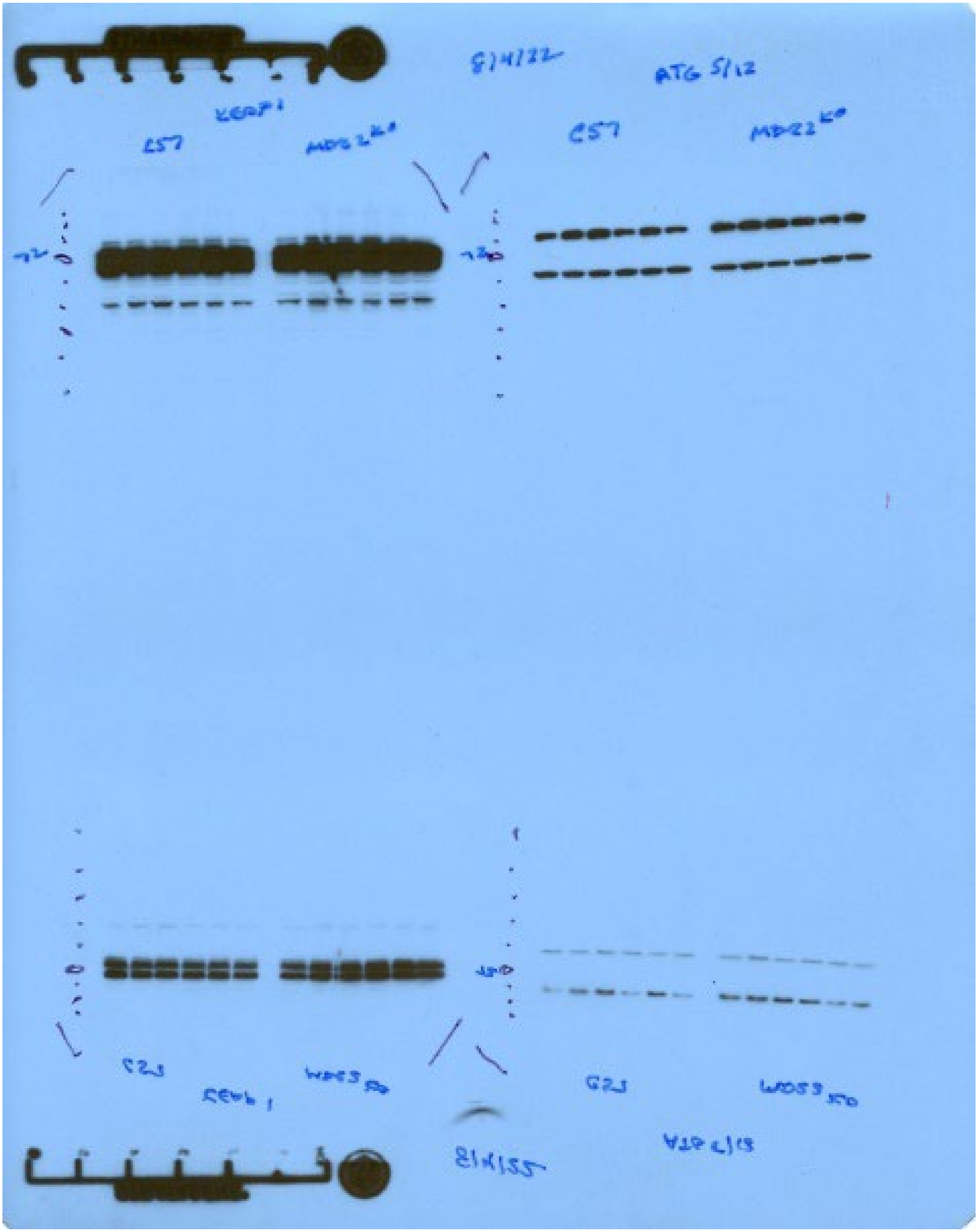
ATG 5/12
C57
C57

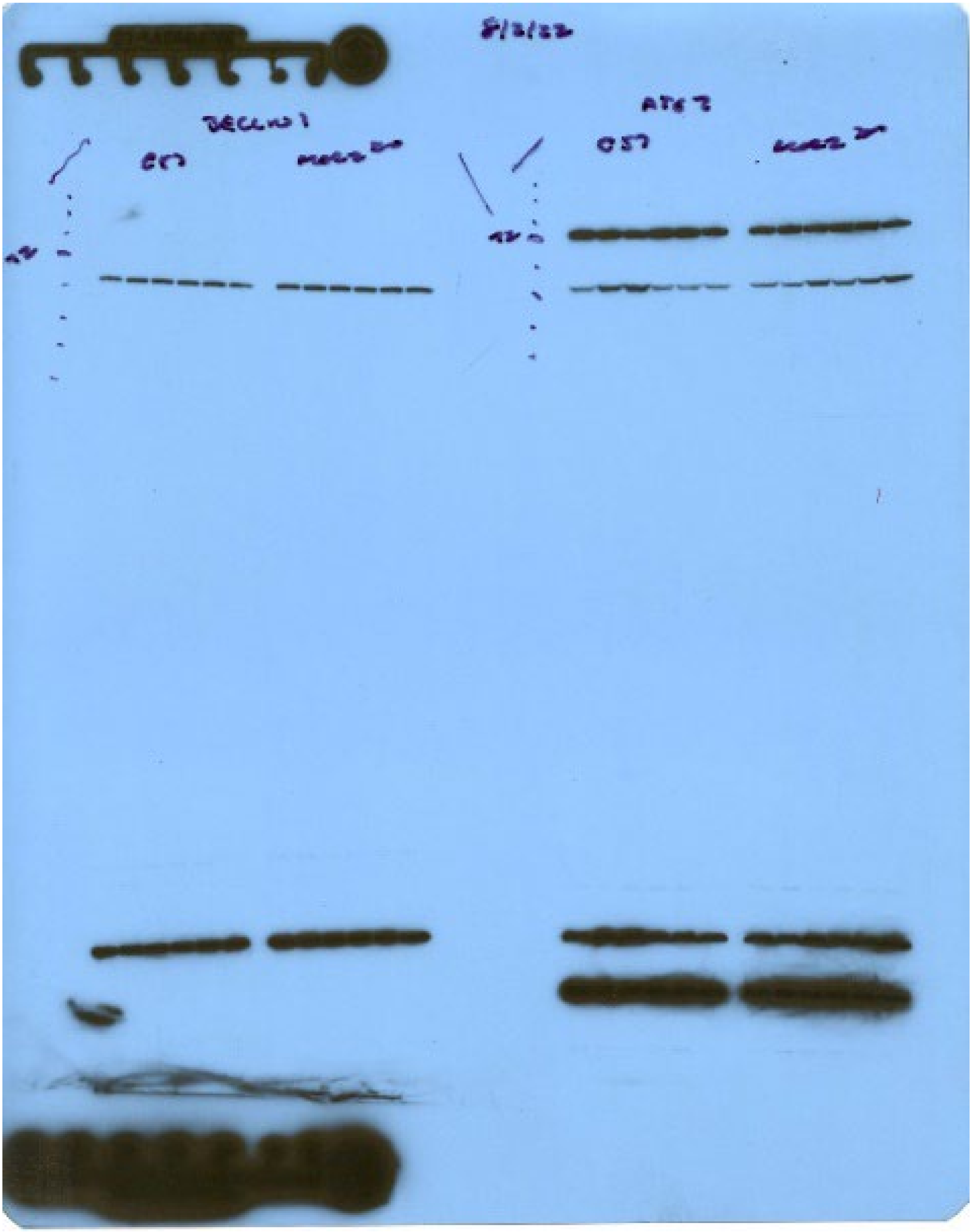
8/3/22
ATG7
C57

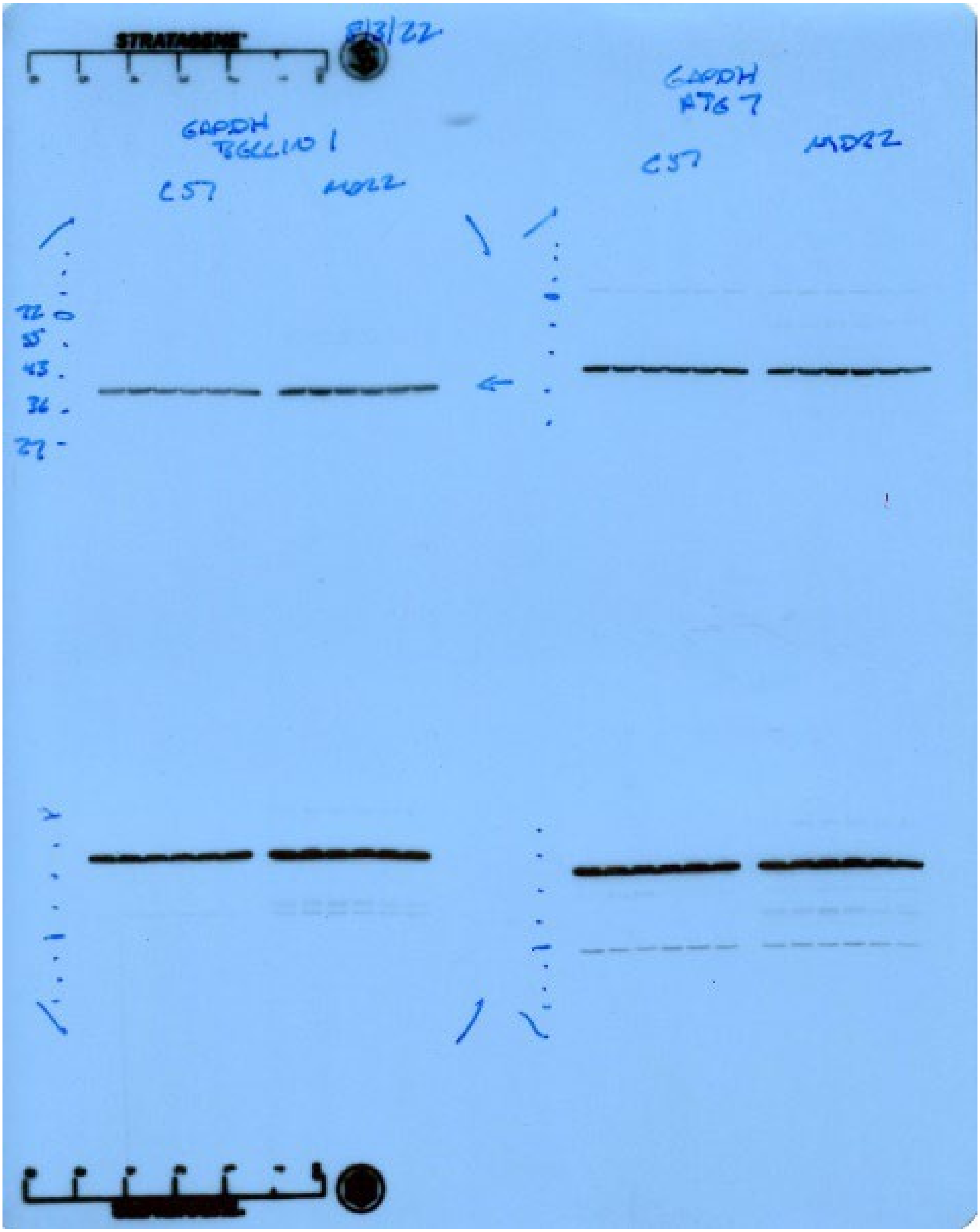

STRATAGENE
8/3/22
GAPDH
C57
GAPDH
ATG7
C57
72
55
43
36
27

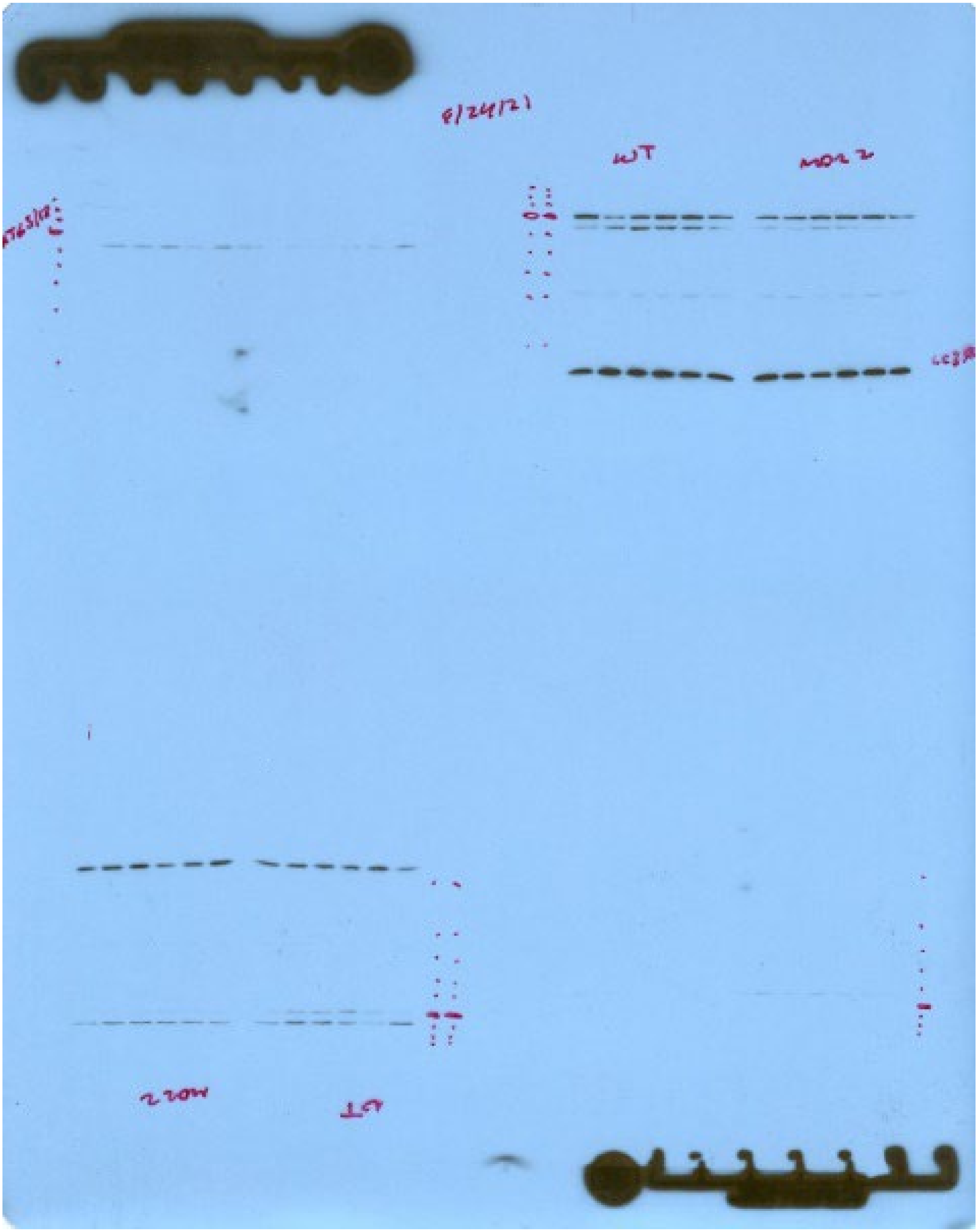
8/24/21
WT

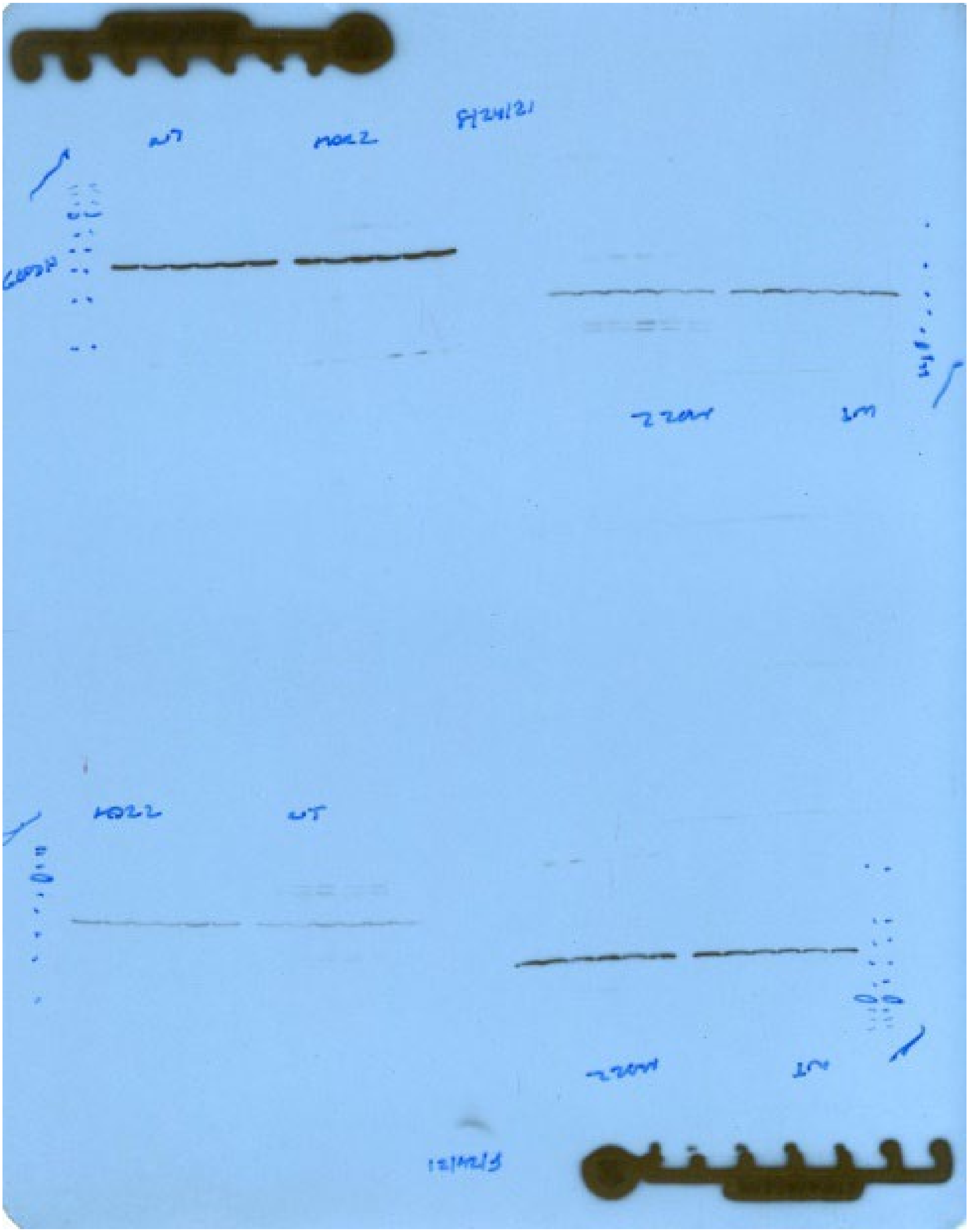

WT
8/24/21

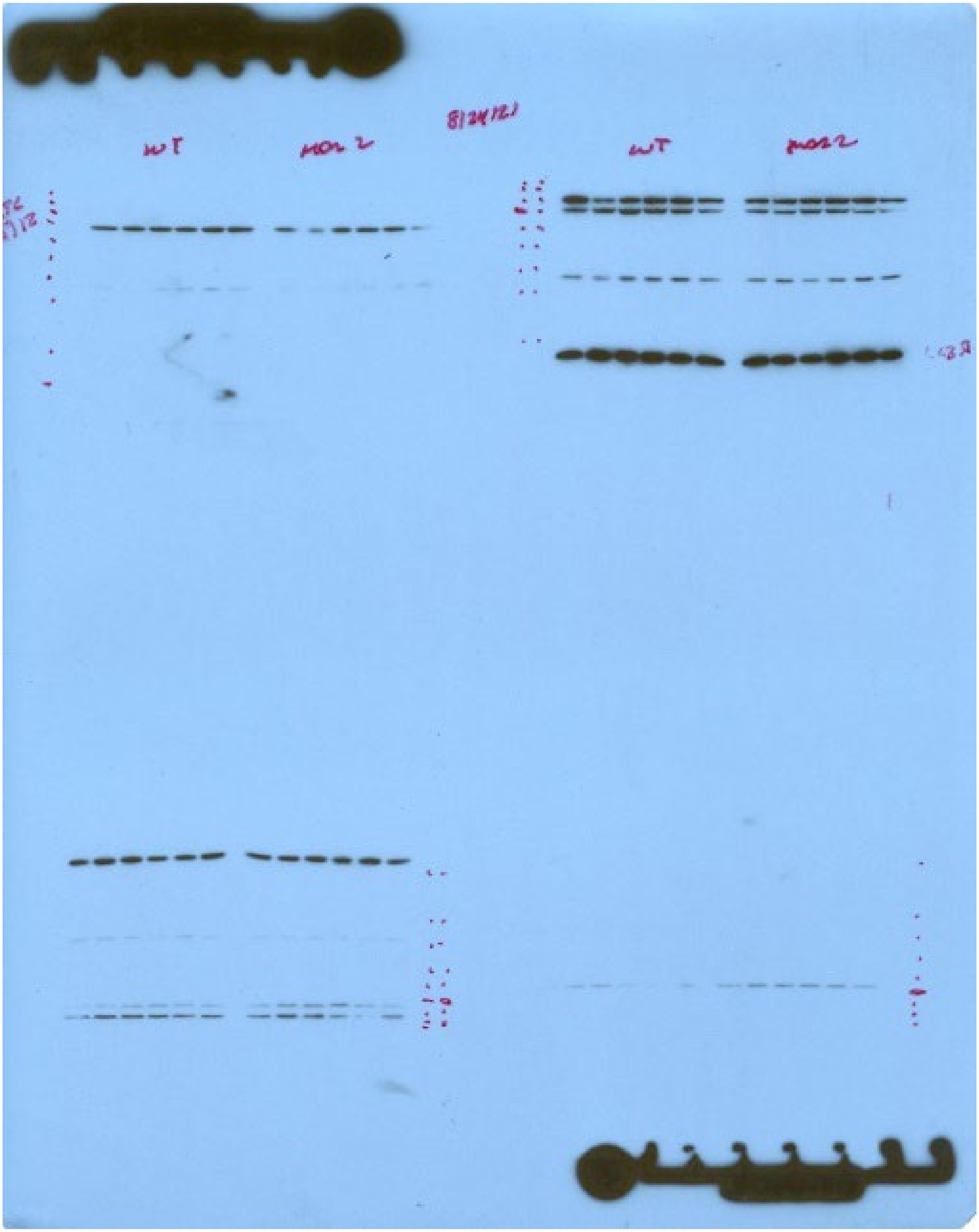
WT
WT

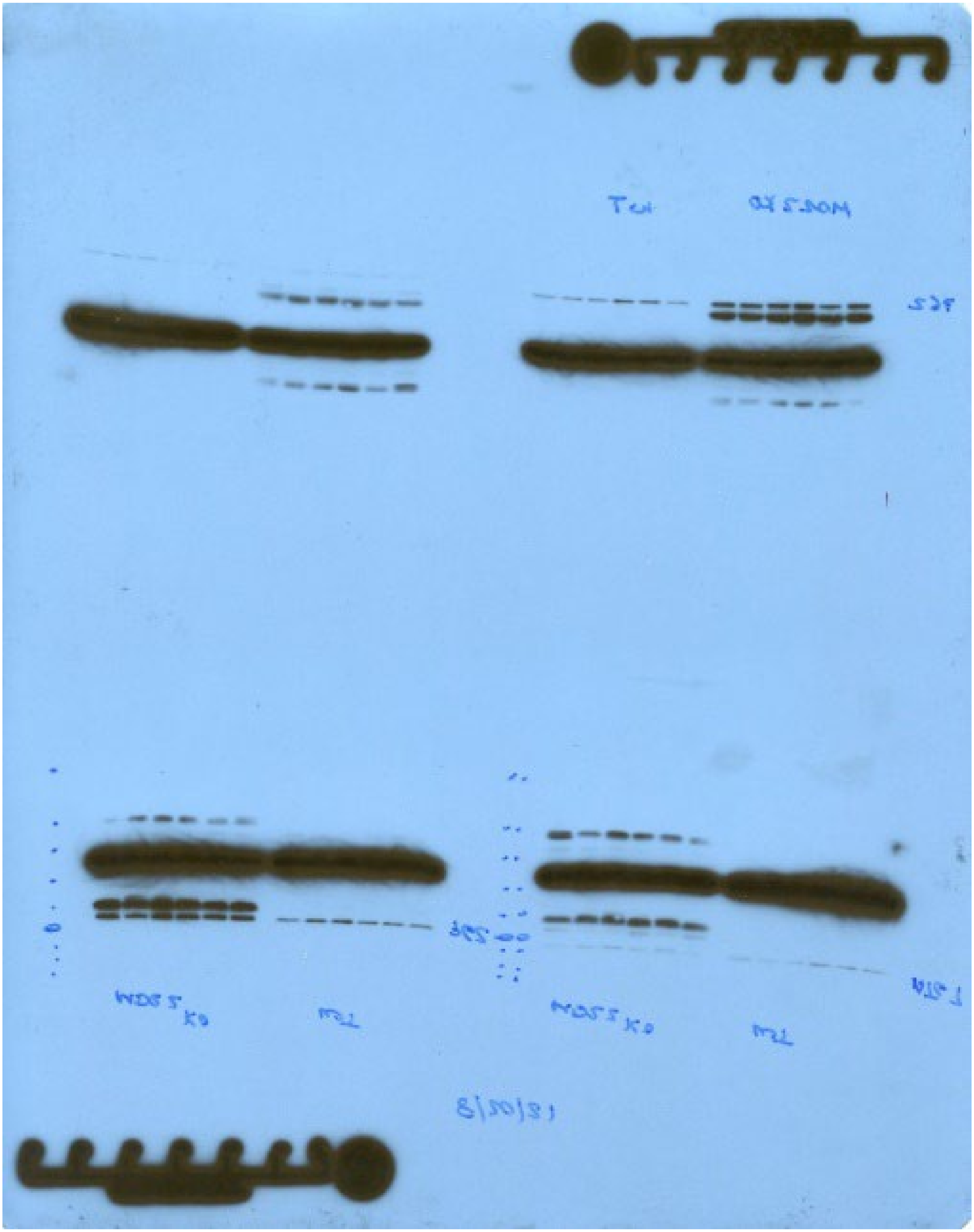

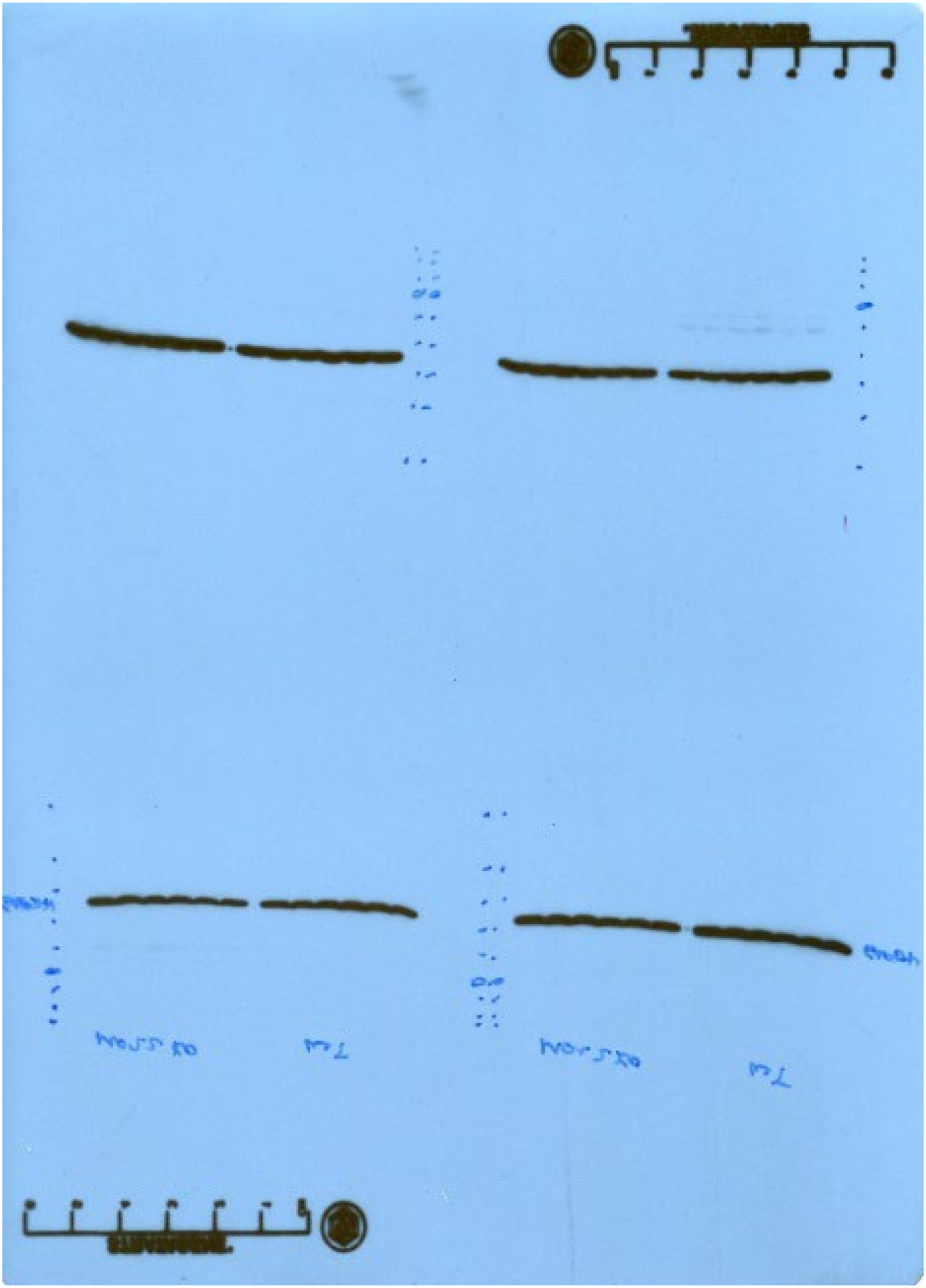

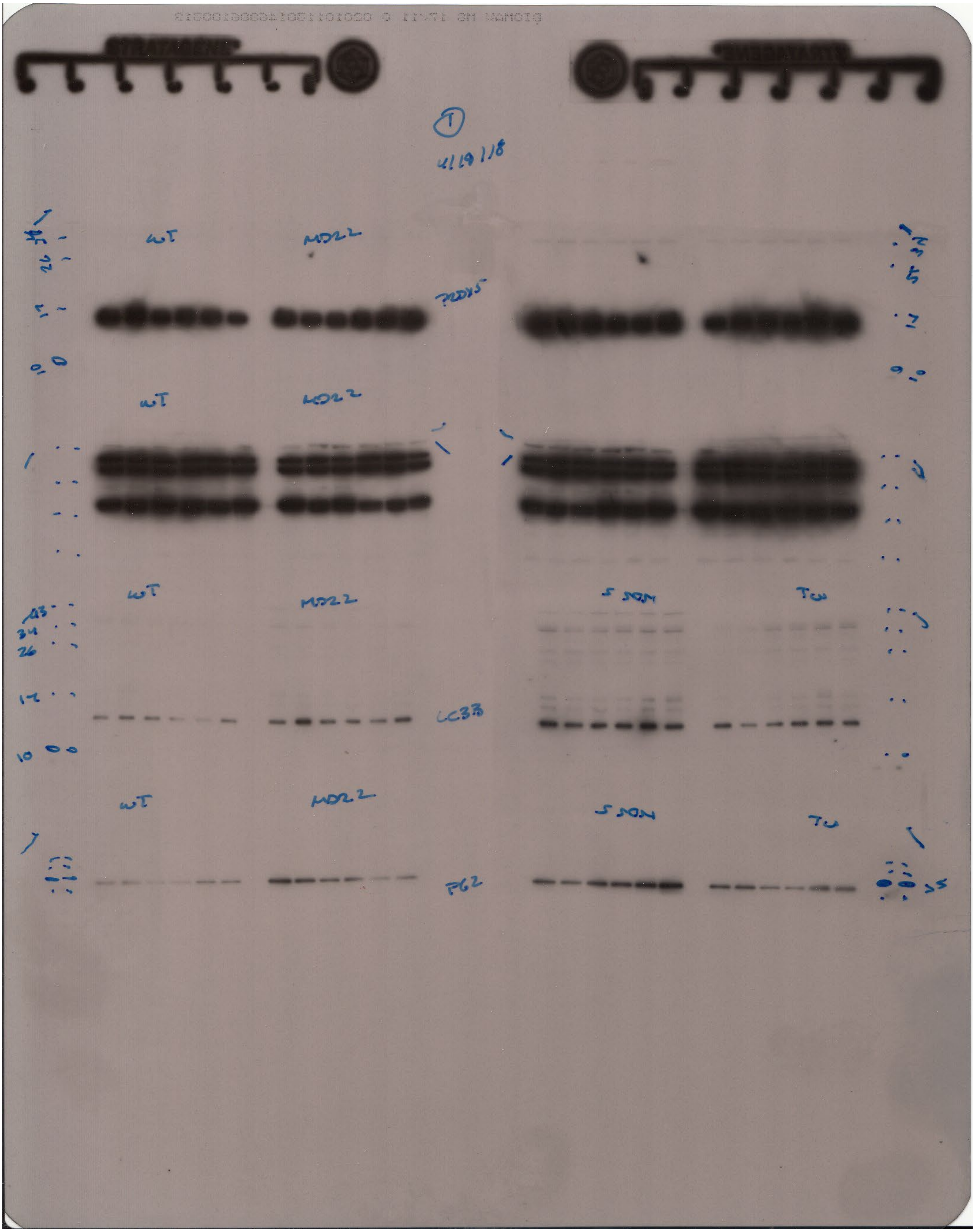

4/19/18
WT
PRDX5
WT
WT
LC3B
WT
P62

Exp 212 **06-07-22**

Purpose: Western blotting of Cbr3, GSTmu, Nqo1 and GSTpi in BDL

Procedures:

a. Resuspended tissue in approximately 400λ of 1mM NaCl + 1/100 mammalian protease inhibitor cocktail (P8340 Sigma) for 15 minutes.
b. Sonicated tissue for 3X15 seconds using handheld sonicator.
c. Add 100λ of 5X cell lysis buffer (500mM NaCl, 5mM EDTA, 500mM Tris pH 8.0 plus 0.5mM Triton X-100.
d. Sonicate an additional 1X15 seconds.
e. Spin samples for 5 minutes at 14000 RPM 4ºC.
f. Quantify by BCA and Aliquot samples.

Gel load order:

1. MW
2. space
3. mouse number 147 Sham 3 Day
4. mouse number 148 Sham 3 Day
5. mouse number 149 Sham 3 Day
6. mouse number 150 Sham 3 Day
7. mouse number 151 Sham 3 Day
8. space
9. mouse number 1 BDL 3 Day
10. mouse number 2 BDL 3 Day
11. mouse number 13 BDL 3 Day
12. mouse number 14 BDL 3 Day
13. mouse number 16 BDL 3 Day
14.

Western blot for
GSTmu
GSTpi
Nqo1
CBR3

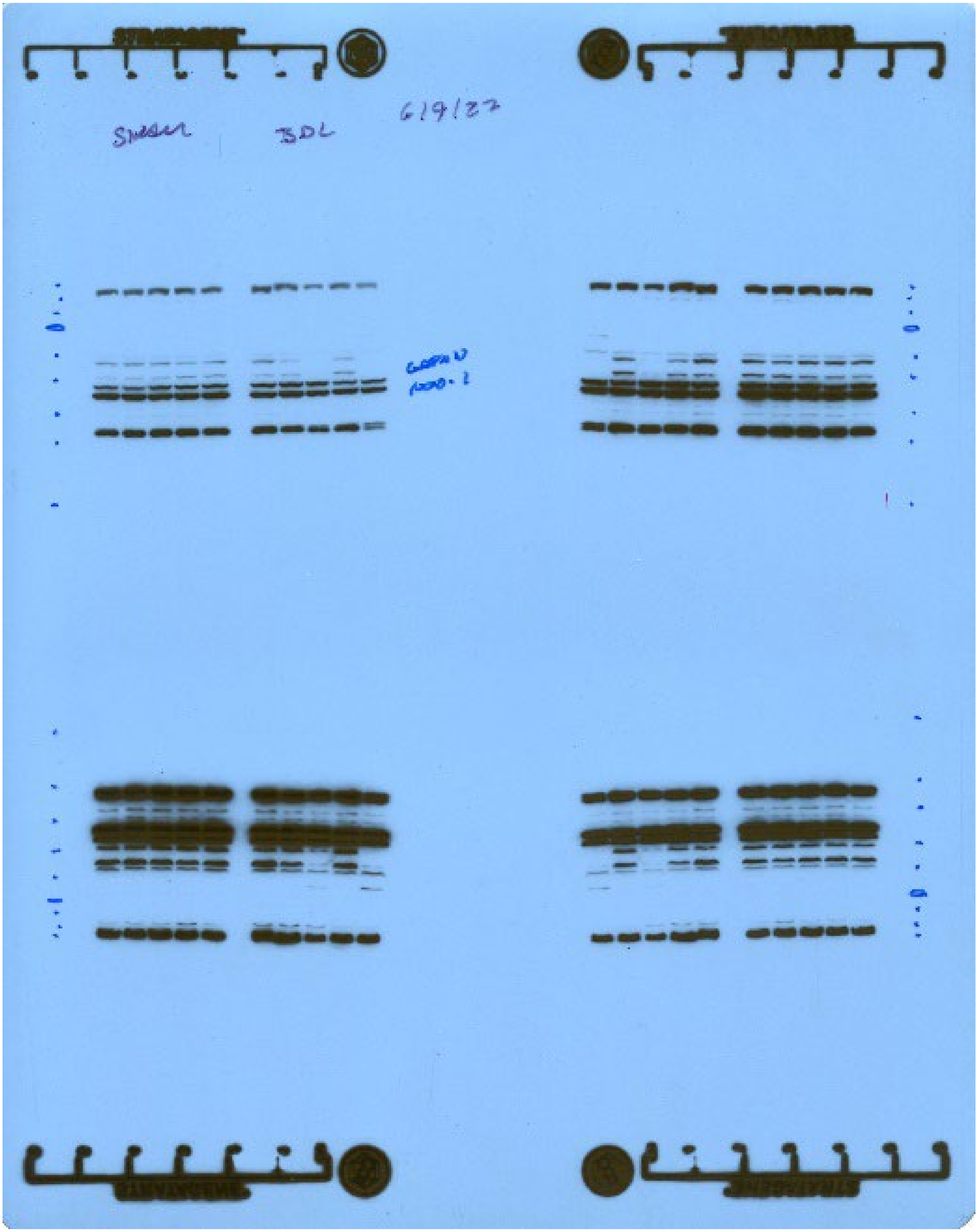
BDL
6/9/22

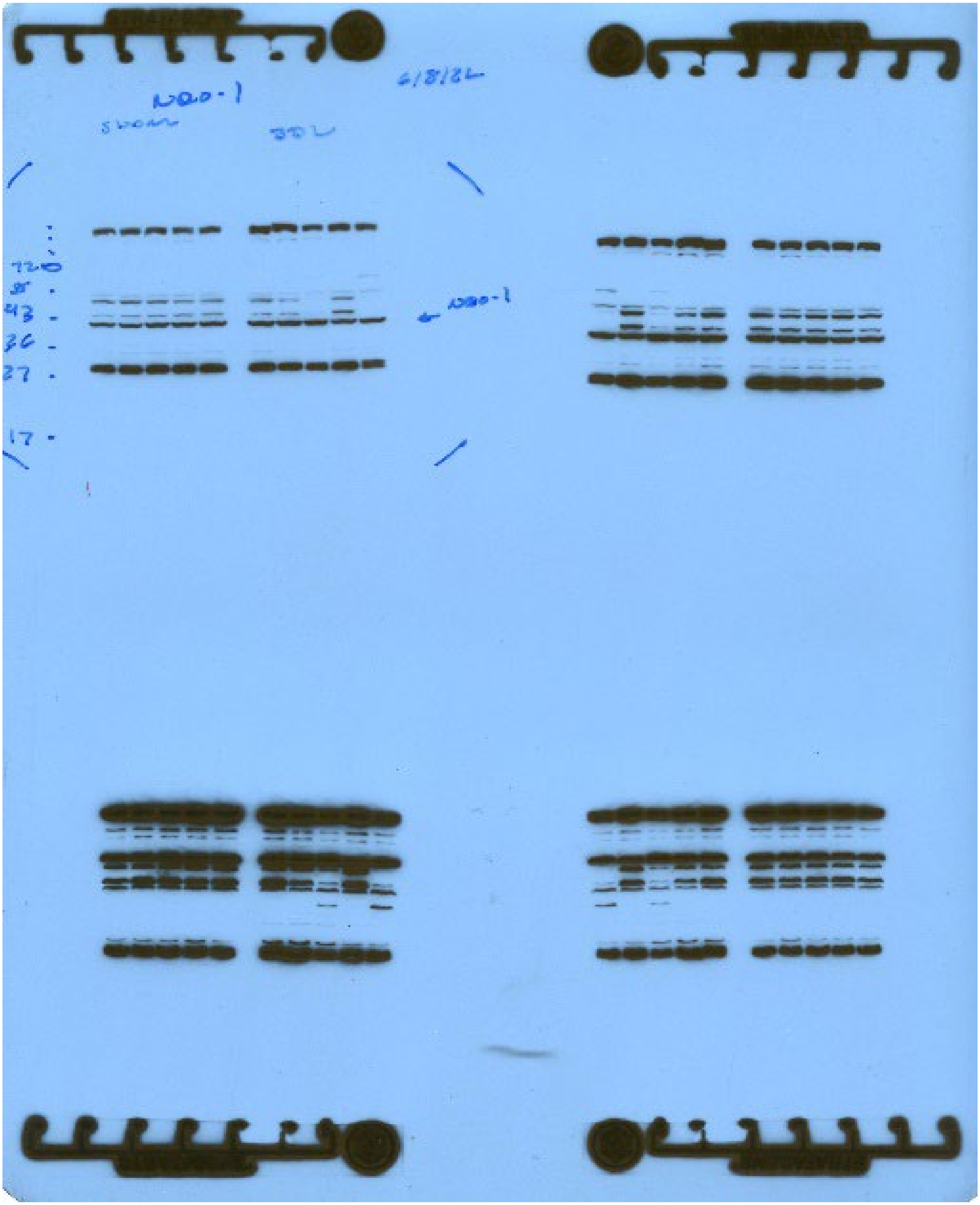

NQO-1
72
43
17
NQO-1

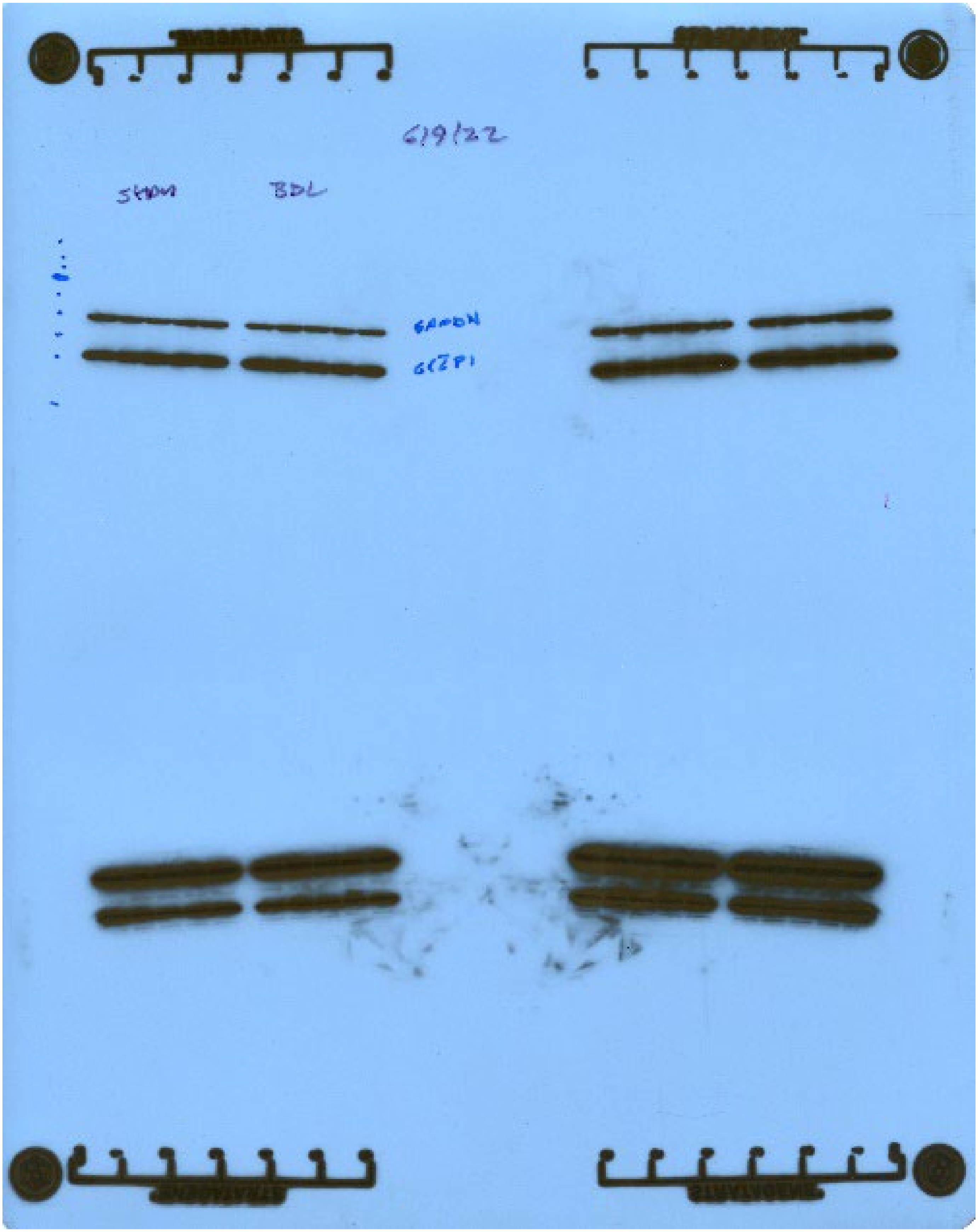
6/9/22
Sham
BDL
GAPDH

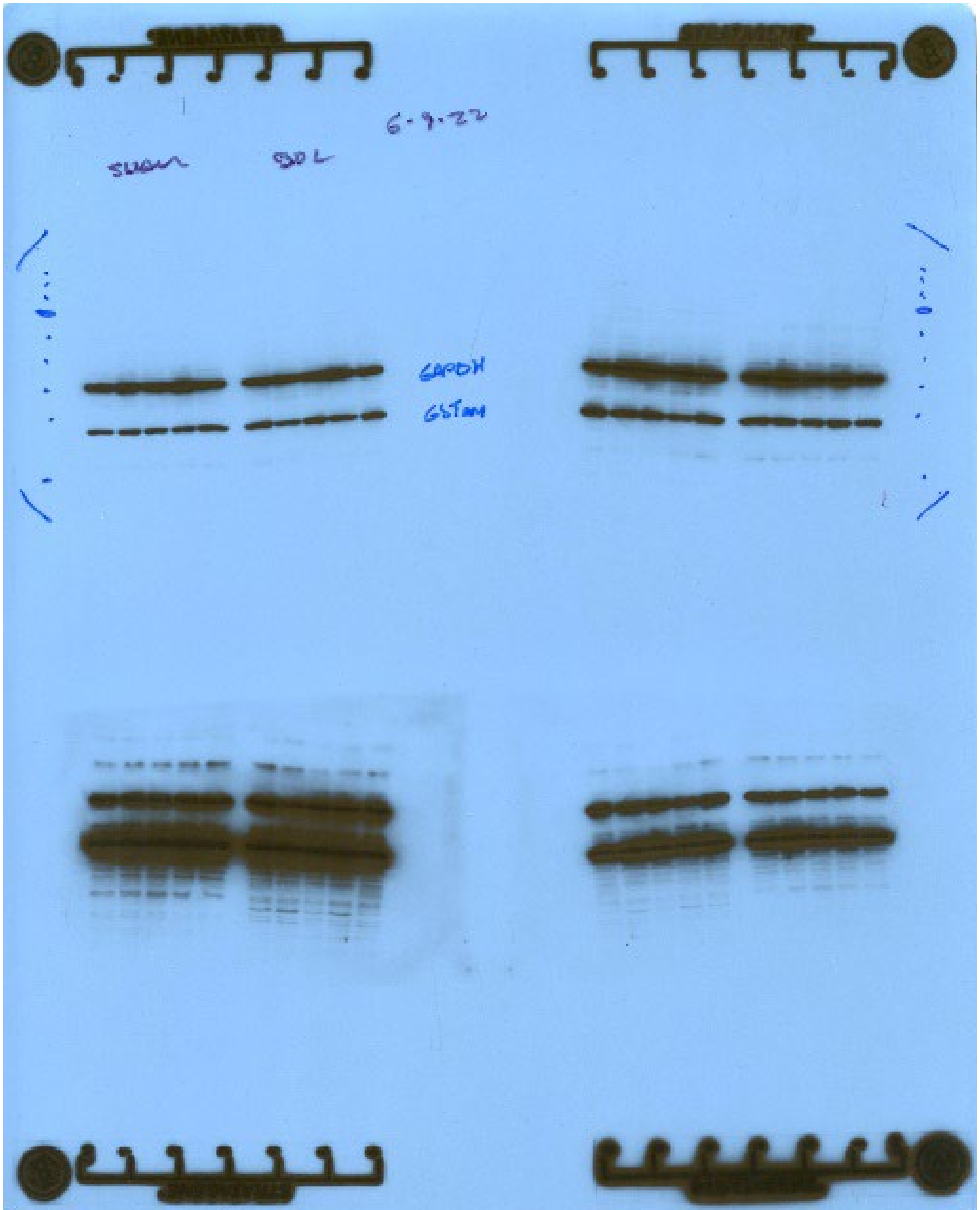
6-9-22
BDL
GAPDH

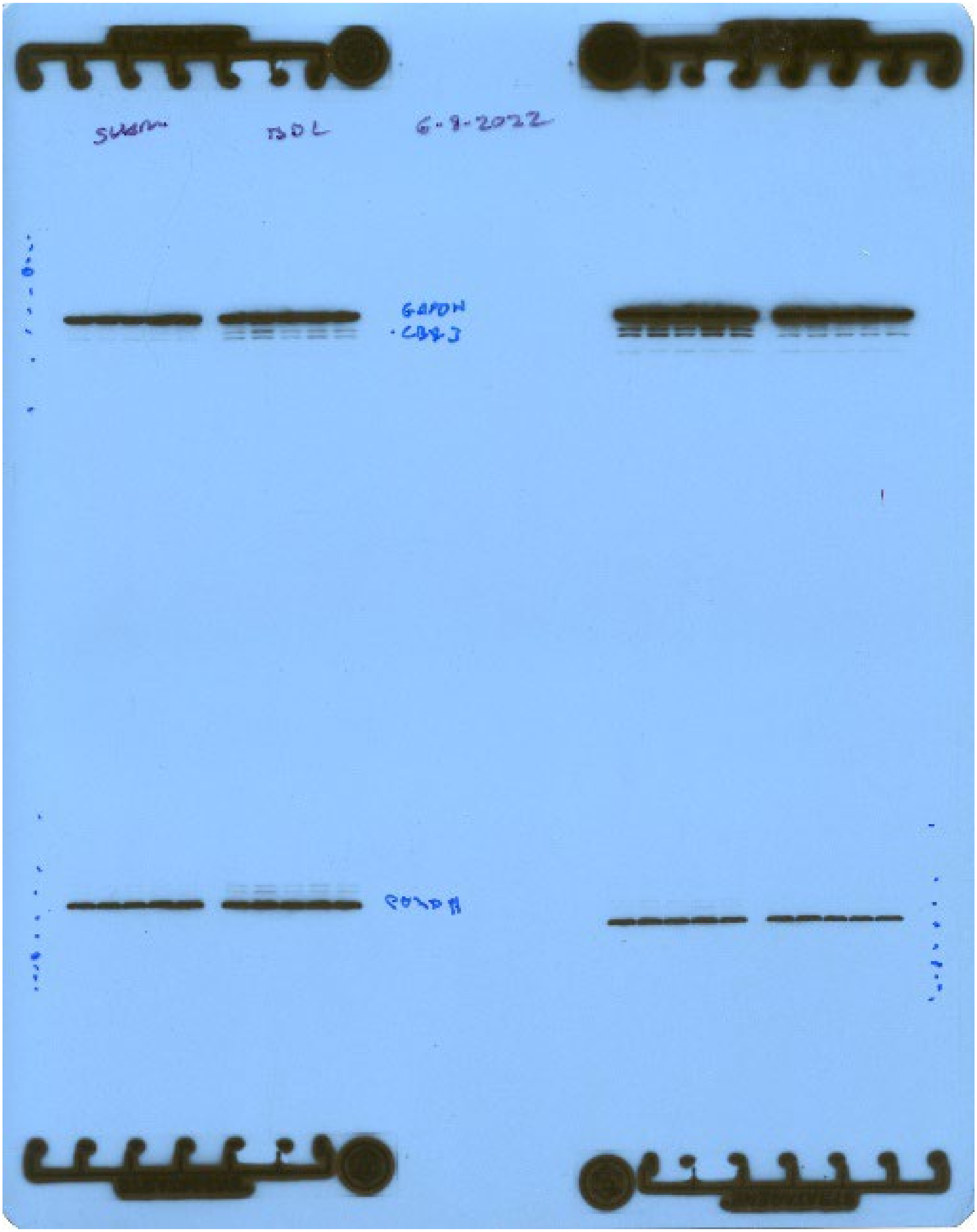
Sham
BDL
6-8-2022
GAPDH

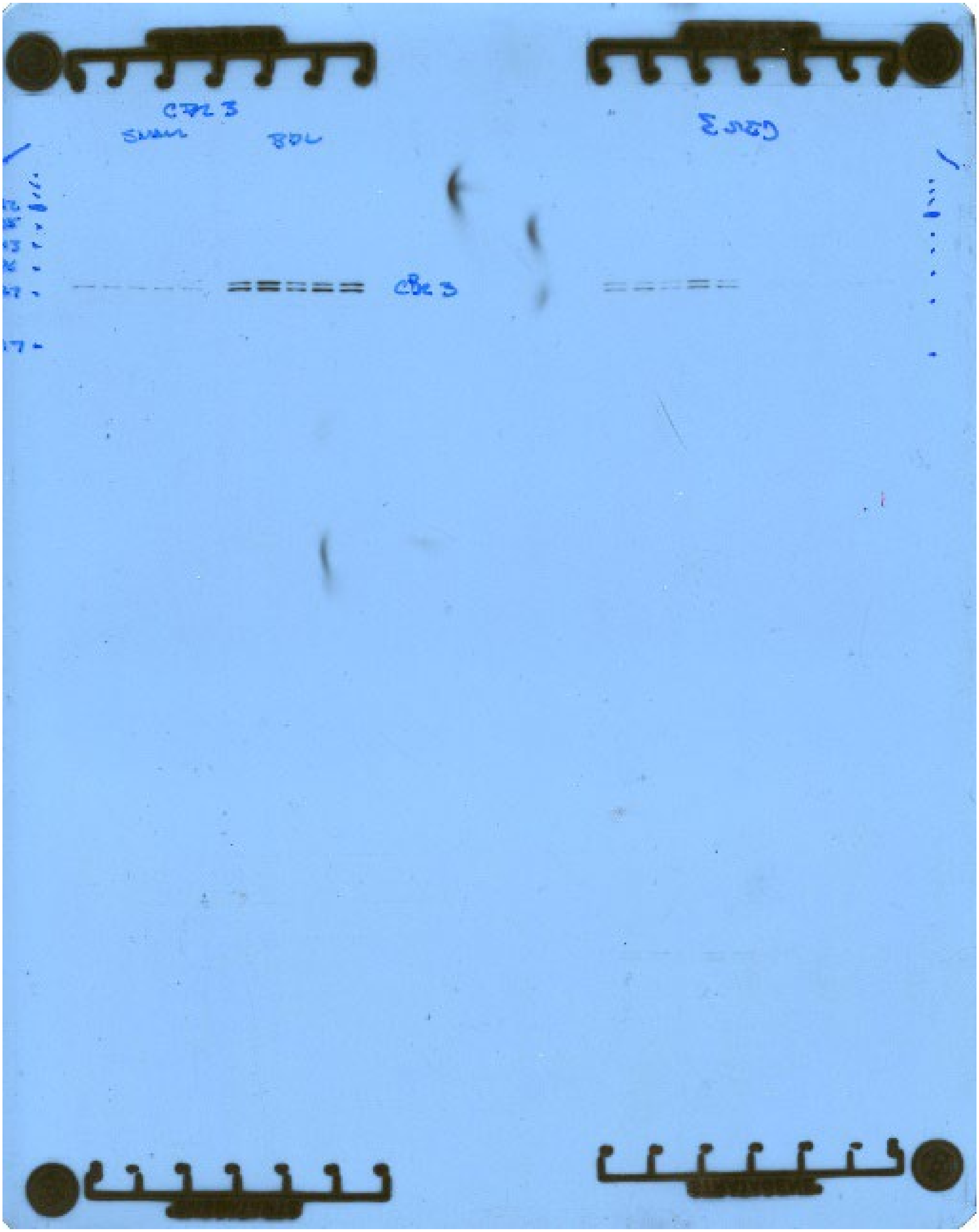

Sham
BDL

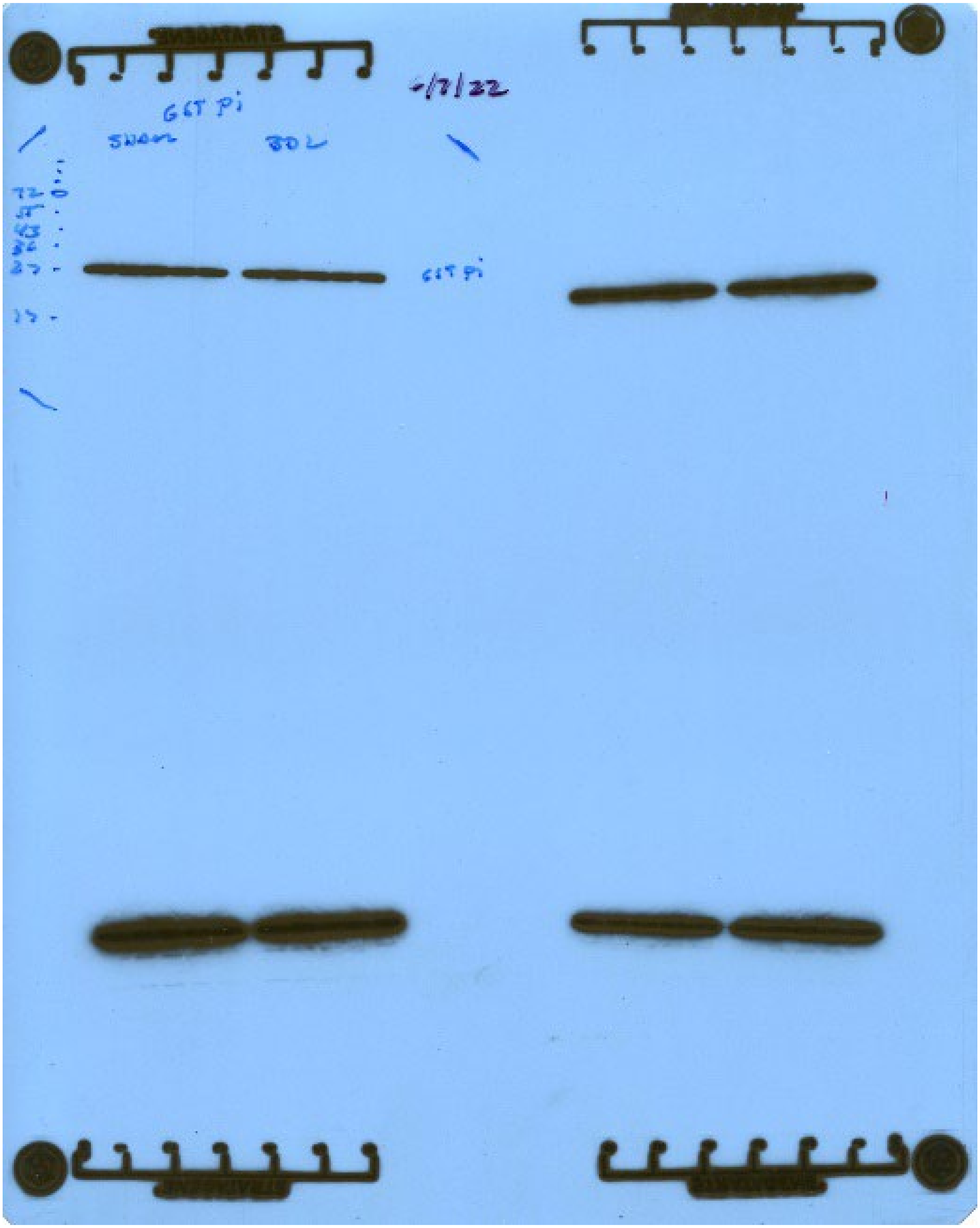
GST pi
72
55
43
36
GST pi

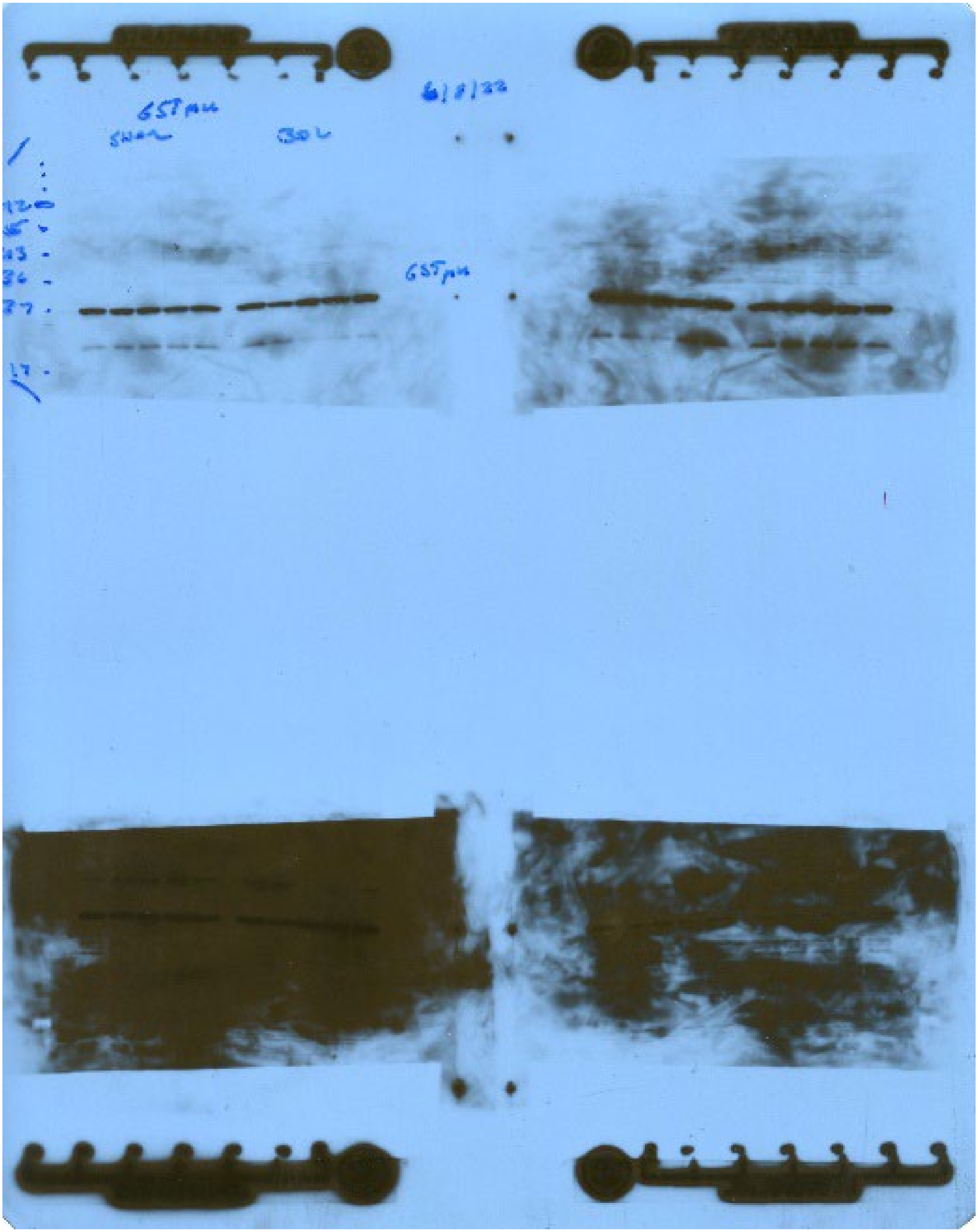
6/8/22
43
36
17

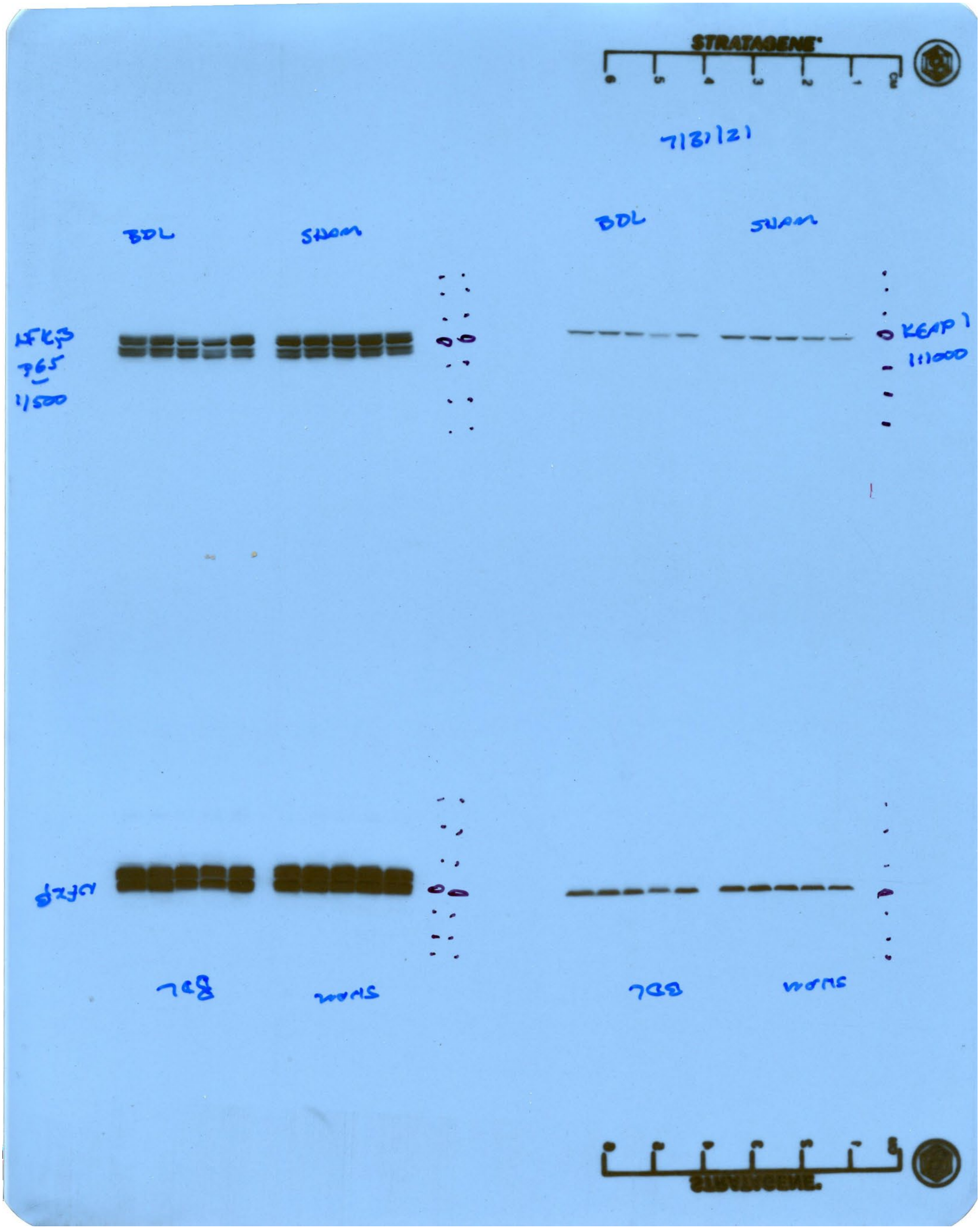
STRATAGENE
7/31/21
BDL
SHAM
NFKB
p65
1/500
BDL
SHAM
KEAP 1
1:1000

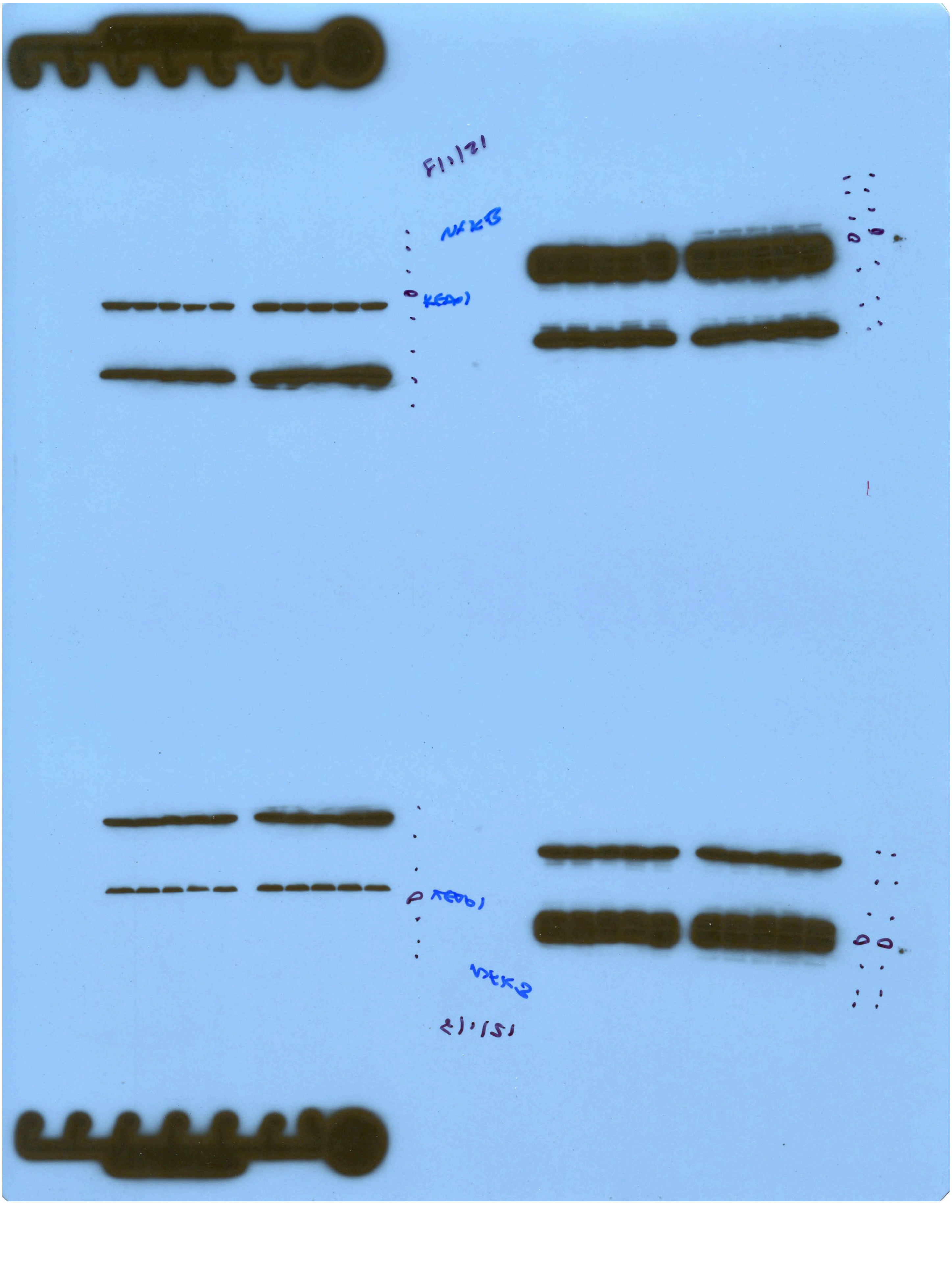
NFKB
Keap1

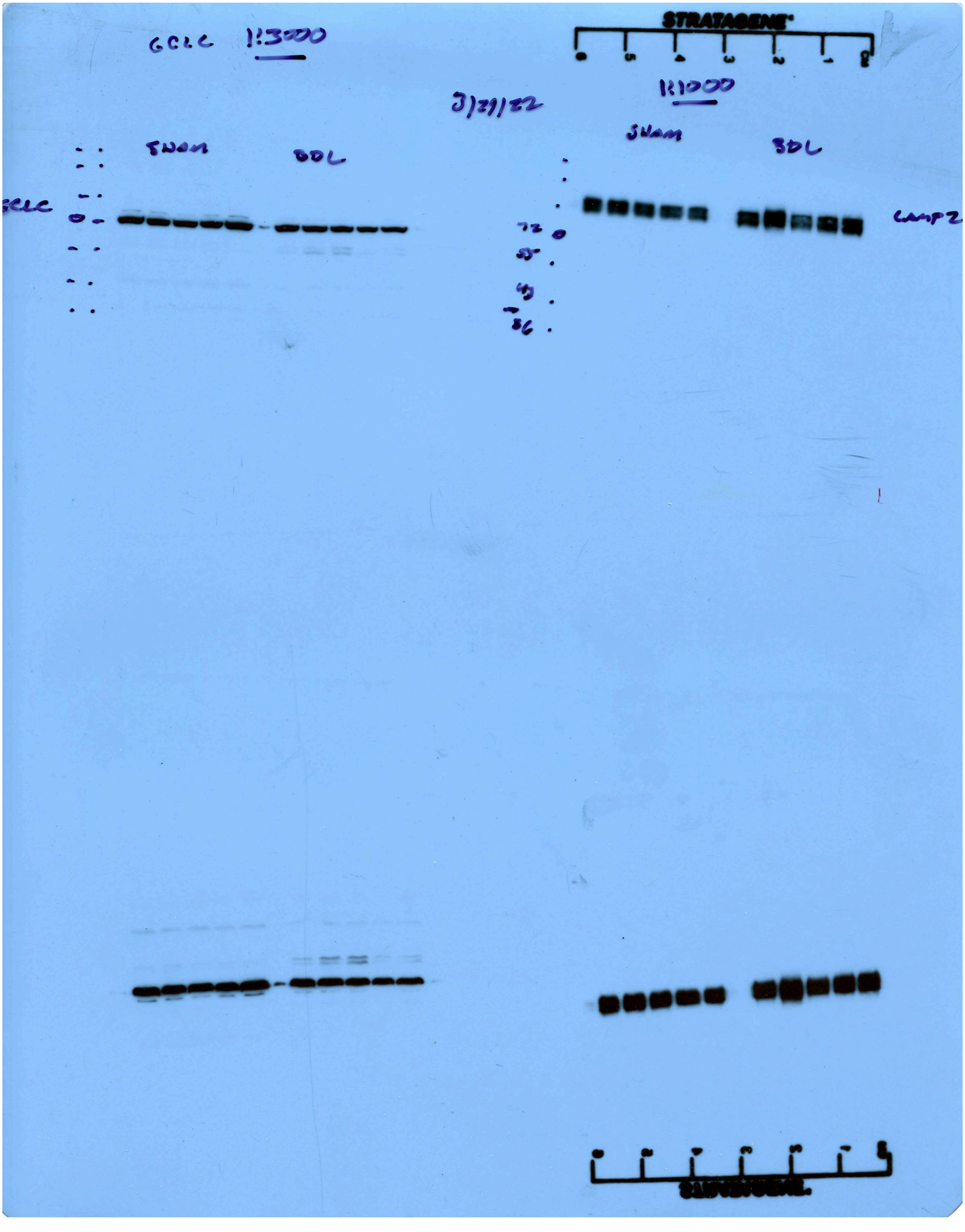
GCLC 1:3000
STRATAGENE
1:1000
SHAM
BDL
GCLC
72
55
43
36
SHAM
BDL
LAMP2

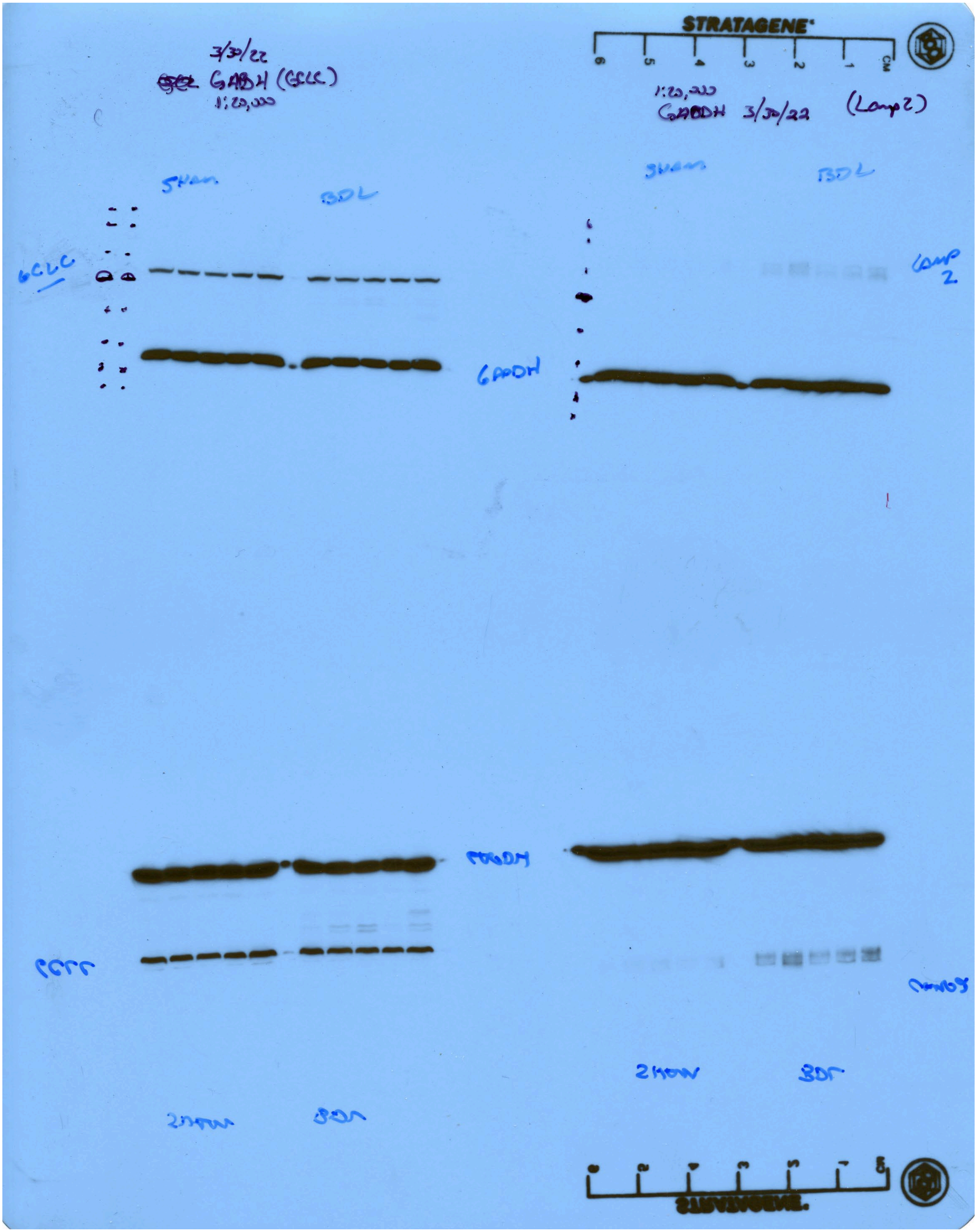
STRATAGENE
3/30/22
GAPDH (GCLC)
1:20,000
Sham
BDL
GCLC
GAPDH
1:20,000
GAPDH 3/30/22
(Lamp2)
Sham
BDL
Lamp 2

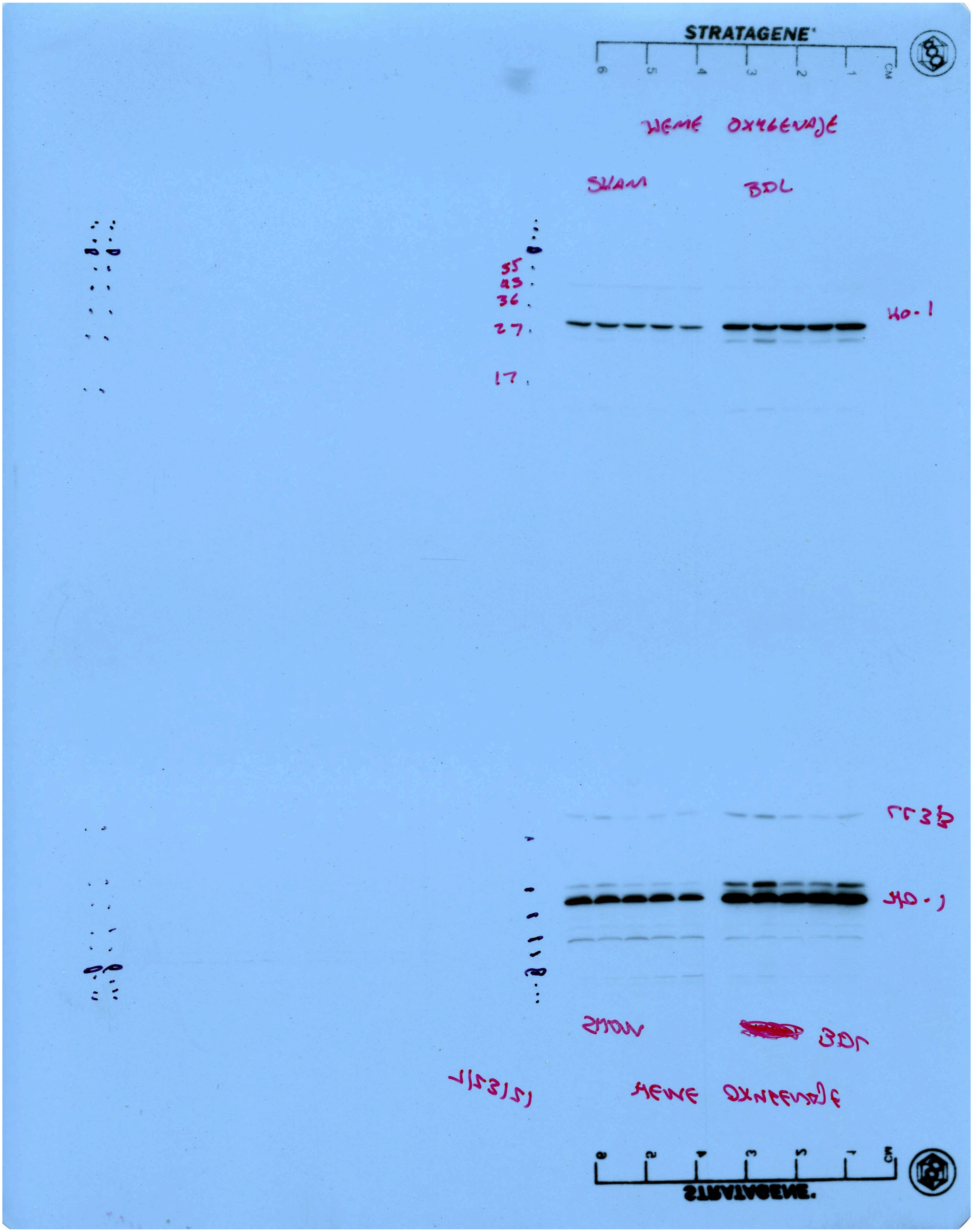
STRATAGENE
HEME OXYGENASE
SHAM
BDL
55
43
36
27
17
HO-1

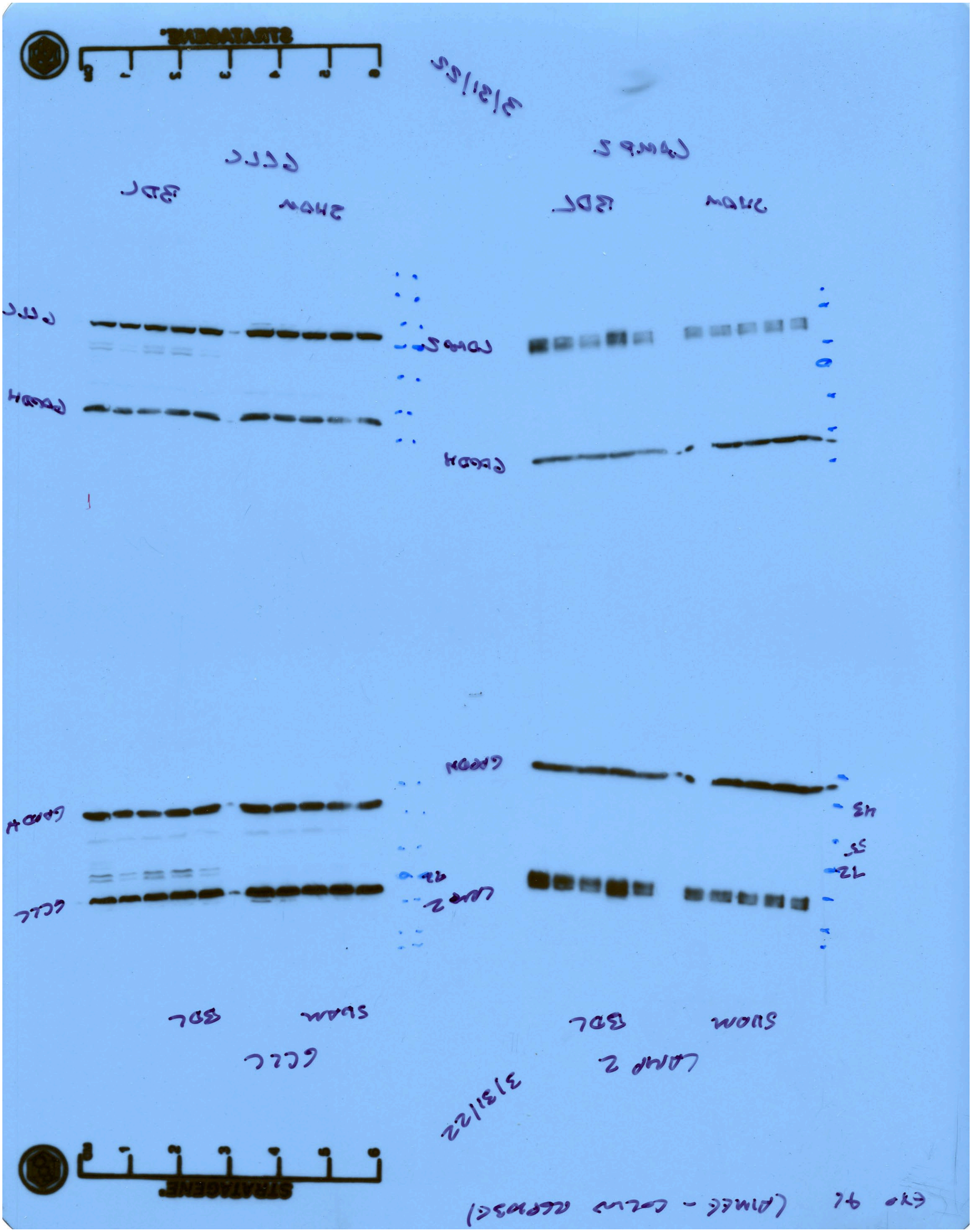
GCLC
LAMP2
BDL
SHAM
GAPDH
3/31/22
43
55
72
